# Identifying glycan motifs using a novel subtree mining approach

**DOI:** 10.1186/s12859-020-3374-4

**Published:** 2020-02-04

**Authors:** Lachlan Coff, Jeffrey Chan, Paul A. Ramsland, Andrew J. Guy

**Affiliations:** 1 School of Science, College of Science, Engineering and Health, RMIT University, 3000 Melbourne, Australia; 2 Department of Immunology, Monash University, 3004 Melbourne, Australia; 3 Department of Surgery Austin Health, University of Melbourne, 3084 Heidelberg, Australia

**Keywords:** Carbohydrate, Glycan, Microarray, Glycobiology, Motif, Machine learning, Frequent subtree mining

## Abstract

**Background:**

Glycans are complex sugar chains, crucial to many biological processes. By participating in binding interactions with proteins, glycans often play key roles in host–pathogen interactions. The specificities of glycan-binding proteins, such as lectins and antibodies, are governed by motifs within larger glycan structures, and improved characterisations of these determinants would aid research into human diseases. Identification of motifs has previously been approached as a frequent subtree mining problem, and we extend these approaches with a glycan notation that allows recognition of terminal motifs.

**Results:**

In this work, we customised a frequent subtree mining approach by altering the glycan notation to include information on terminal connections. This allows specific identification of terminal residues as potential motifs, better capturing the complexity of glycan-binding interactions. We achieved this by including additional nodes in a graph representation of the glycan structure to indicate the presence or absence of a linkage at particular backbone carbon positions. Combining this frequent subtree mining approach with a state-of-the-art feature selection algorithm termed minimum-redundancy, maximum-relevance (mRMR), we have generated a classification pipeline that is trained on data from a glycan microarray. When applied to a set of commonly used lectins, the identified motifs were consistent with known binding determinants. Furthermore, logistic regression classifiers trained using these motifs performed well across most lectins examined, with a median AUC value of 0.89.

**Conclusions:**

We present here a new subtree mining approach for the classification of glycan binding and identification of potential binding motifs. The Carbohydrate Classification Accounting for Restricted Linkages (CCARL) method will assist in the interpretation of glycan microarray experiments and will aid in the discovery of novel binding motifs for further experimental characterisation.

## Background

As one of the four major classes of biomolecules, carbohydrates are present in all organisms and play crucial roles in biomolecular interactions. Organisms polymerise simple sugars to yield oligo- and polysaccharides, which are typically termed glycans when attached to proteins and lipids. Glycans may be composed of several sugar residues with various glycosidic linkages, often forming branched structures. Consequently, there are a myriad of glycan structures that have arisen in organisms, with distinct glycosylation patterns observed between evolutionary clades. Glycoforms can even differ between individuals. Aberrant glycosylation is a hallmark of cancer, and a body of research has focused on the identification of glycan biomarkers as diagnostic and prognostic tools for use in oncology [1, 2]. Additionally, carbohydrate determinants are frequently involved in host–pathogen interactions. Notable examples of this include the attachment of influenza virions to host sialic acid residues and the recognition of pathogens by mannose receptors and anti-carbohydrate antibodies [3, 4]. The mannose receptor, along with DC-SIGN, is an example of a C-type lectin present on the surface of immune cells. Lectins can be defined as ‘proteins that possess at least one noncatalytic domain that binds reversibly to a specific mono- or oligosaccharide’, excluding enzymes (e.g. glycosyltransferases) and carrier proteins [5]. Due to their broad selectivities, lectins are also distinct from other glycan-binding proteins that recognise specific carbohydrate antigens, such as antibodies and T-cell receptors. The carbohydrate-binding properties of plant lectins have been exploited by scientists for a number of laboratory techniques, including histochemical staining, affinity chromatography, and identification of biomarkers. For example, *Lens culinaris* agglutinin (LCA)-reactive *α*-fetoprotein (a glycoform termed ‘AFP-L3’) is an FDA-approved biomarker for the risk assessment of hepatocellular carcinoma [6, 7]. However, the selectivities of lectins for glycan motifs are often poorly defined, which undermines confidence in glycan profiling.

As complex structures, carbohydrates are often best suited to computational analyses. Several open-access resources exist for structural analysis of carbohydrates [8], but relatively few for analysis of glycan motifs. Importantly, it is the branched nature of glycans that renders them unsuitable for motif analysis techniques developed for linear nucleic acid and protein sequences. Instead, methods developed for analysis of graph structures are typically used, included tree kernel methods and subtree mining approaches. For example, a tree kernel method was shown to reliably classify human blood glycans into different human blood components [9]. In addition to using glycan structures to classify cell or tissue origin, understanding the interactions between glycan-binding proteins and their ligands can be fundamental to a variety of scientific inquiries, including human health and disease. The Consortium for Functional Glycomics (CFG) conducted thousands of experiments with standardised glycan microarrays and has made these data publicly available online [10]. However, few attempts have been made to conduct meaningful analyses across these large datasets, and glycan-specific data mining tools would aid such work. To this end, GlycoSearch (later MotifFinder) was developed to allow glycans from CFG datasets to be mined for predefined motifs [11]. While this algorithm has been applied to a global analysis of the CFG glycan microarray data [12], it does not allow for the discovery of new motifs.

For the detection of characteristic binding motifs within a set of glycans, frequent subtree mining approaches have been employed by other researchers [13, 14]. Frequent subtree mining is a technique that is used to find a set of characteristic motifs (or subtrees) that are present at a defined frequency within a set of glycans (or other graph-like structures). Ideally, identified motifs should be present at high frequency within a set of positive binding glycans but relatively absent within negative binders. Hashimoto et al. developed the first frequent subtree mining algorithm for glycans in 2008 [13], which was later made available at the Resource for Informatics of Glycomes at Soka (RINGS) and used to discover sulfated structures as novel binding determinants of influenza virions from CFG glycan microarray data [15]. The GlycanMotifMiner (GLYMMR) followed in 2012, which incorporates a statistical method of distinguishing binding glycans from non-binding glycans and considers both binding and non-binding glycans when predicting motifs [14]. Using a different approach, the Multiple Carbohydrate Alignment with Weights (MCAW) tool aligns glycans in a analogous manner to multiple alignments of DNA or protein sequences and has been used to identify patterns in binding glycans from the CFG glycan microarray data [16, 17]. Ultimately, these algorithms aim to define the selectivities of lectins and other glycan-binding proteins using existing experimental data.

In this work, we introduce a novel frequent subtree mining approach for identifying binding motifs, Carbohydrate Classification Accounting for Restricted Linkages (CCARL), which we have tested on glycan microarray data from the CFG. This approach incorporates a new method for distinguishing binding and non-binding glycans, as well as an adapted glycan notation, which includes restrictions on connecting residues. For example, a mannose residue may form glycosidic linkages from -OH groups on its carbon 2, 3, 4, or 6, and so any of these non-existent linkages are denoted by a cross in place of a residue symbol and the corresponding carbon numbers in our modified Symbol Nomenclature for Glycans (SNFG). Klamer et al. 2017 employed a ‘free’ modifier in their glycan motif syntax [18], but we refer to these as ‘restricted linkages’ in the context of specifying motifs that do not form particular chemically possible glycosidic linkages. This representation allows the discrimination of terminal and non-terminal motifs. As motifs are often only binding determinants if present at the non-reducing terminal of glycans, this notation enhances the performance of prediction tools trained using these motifs. We tested this method across a range of commonly used lectins and were able to both identify key binding motifs as well as accurately predict binding of a test set of glycans.

## Methods

### Overview

**Fig. 1:**
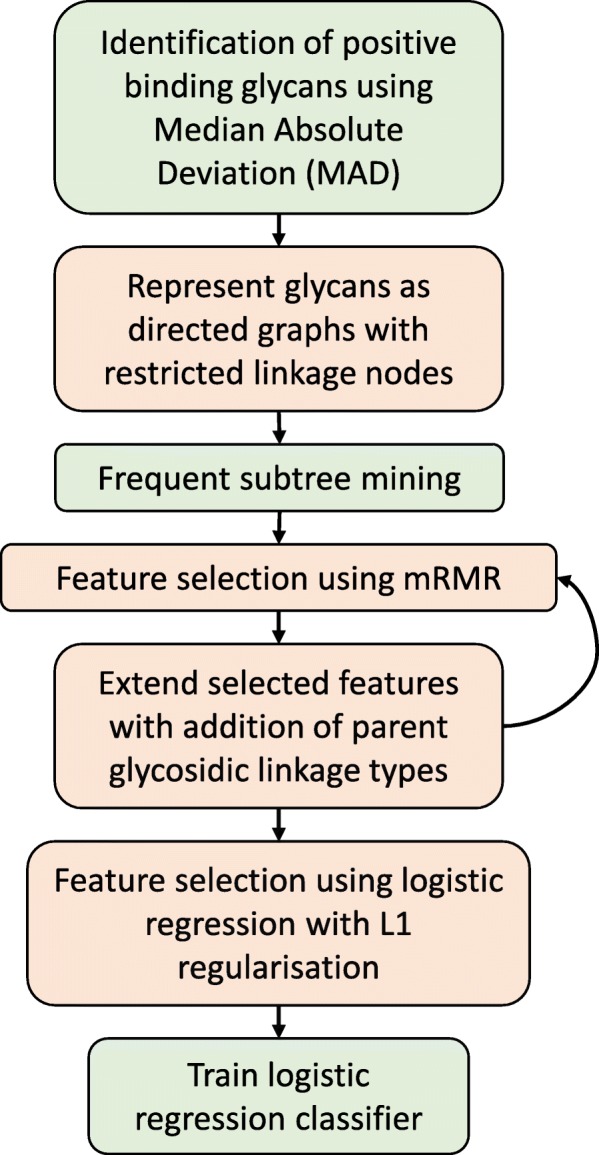
Workflow for identification of key binding motifs from glycan microarray data and construction of predictive classifier

To identify key binding motifs from a glycan microarray experiment, we represented glycans as directed acyclic graphs with additional nodes to indicate the absence of a linkage at particular backbone carbon positions. These additional nodes are termed ‘restricted linkages’. Using this directed graph representation, we extracted a large set of possible motifs using a frequent subtree mining approach, followed by feature selection to identify a smaller set of likely motifs. Identified motifs were augmented by adding information on parent edge type, meaning the anomeric descriptor (*α* or *β*) at the reducing end of the motif, and the process of feature selection repeated using this augmented set of features. An additional round of feature selection was performed using logistic regression with L1 regularisation. Finally, we trained an unregularised logistic regression model to predict the probability of an unknown glycan binding to a particular protein (Fig. 1).

### Data sources

Glycan microarray data were obtained from the Consortium for Functional Glycomics (CFG) (http://www.functionalglycomics.org/; accessed 27/11/2018), with all glycan microarray data downloaded using custom Python scripts. CFG microarray data were only available in Excel format, so additional data cleaning was required to extract relevant data into a format amenable to large-scale analysis. All scripts are available at https://github.com/andrewguy/CCARL.

### Determination of positive binding glycans from glycan microarray data

To identify positive binding glycans from a glycan microarray experiment, we made two key observations from CFG microarray data. Firstly, that the Relative Fluorescence Unit (RFU) values for non-binding glycans are usually approximately log-normally distributed. Secondly, that there are usually considerably more non-binding glycans than positive binders, such that the median RFU value is close to the median for the background distribution. Based on these observations, we use an outlier detection technique based on Median Absolute Deviation (MAD) scores to identify values that fall outside of the background distribution [19]. MAD is a robust measure of dispersion, being unaffected by a small number of large outliers. This makes it suitable for identifying outliers/positive binders, as the large RFU values for positive binders will have little to no effect on the MAD calculated for a set of data.

We firstly transformed RFU values according to:

$$ x_{i} = \log_{10}[RFU_{i} - \min(RFU) + 1] $$

where min(*RFU*) is the minimum RFU value observed in that particular glycan microarray experiment. Median Absolute Deviation was then calculated using

$$ MAD = \text{median}(|x_{i} - \tilde{x} |) $$

where $\tilde {x}$ is the median of the transformed data. A modified *z*-score is then calculated for each point *x*:

$$ M_{i} = \frac{0.6745(x_{i} - \tilde{x})}{MAD} $$

where the factor of 0.6745 is the approximate *z*-score at the 75th percentile.

This modified *z*-score is analagous to a standard *z*-score, except that it is calculated using the median and MAD value rather than the mean and standard deviation.

Data points with modified *z*-scores above a threshold value are assigned as outliers (i.e. positive binders). For data arising from CFG glycan microarrays, we have used a threshold of *M*_*i*_>3.5 to assign positive binders, and 1.5<*M*_*i*_<3.5 to assign intermediate binders. All intermediate binders were ignored for the purposes of motif identification and classifier training, as it is unclear if these belong to the negative or positive class and we wished to avoid contaminating either the positive or negative binding classes.

Importantly, we note that MAD is relatively insensitive to large numbers of outliers, making it suitable for this sort of task. In practice, this method peforms well for most CFG glycan microarrays, with the only exceptions being cases in which the positive class contains roughly half (or more) of the data points. These occur infrequently enough that we suggest a manual assignment of binding thresholds (using domain-specific knowledge), if these situations arise.

### Generation of training and test datasets

For each glycan binding microarray being examined, positive, negative, and intermediate binding classes were assigned using the MAD outlier detection method. Data points with intermediate binding were discarded, and the remaining data points split into training and test datasets (80%/20% split). This ratio was chosen to maximise the amount of training data while ensuring sufficient positive data points were present in the test set for effective method evaluation. Data were stratified during this process to ensure a consistent ratio of positive to negative binders in each dataset. The training dataset was used for selection of motifs and training of a final classifier. The test dataset was only used for evaluation of the final classification model. The test and training datasets used for this study are provided in Additional file 6.

### Representing glycans as directed graphs with restricted linkage nodes

Standard approaches to motif detection from glycan microarray data usually involve finding some frequent subtrees that are present at high frequency in a positive binding set but are relatively absent in a negative binding set. Within these approaches, glycans are typically represented as directed graphs (or rooted trees) with sugar residues represented as nodes and linkage types represented by edges. We propose a modification to this approach in which additional information on the presence/absence of connecting residues is included (see Additional file 2 for an example). This is indicated by the presence of a restricted linkage node at any position that doesn’t have a connecting residue, but is capable of supporting a connection (i.e. there are other glycans in the dataset that contain that linkage). This allows identification of motifs that are dependent on subtree location (e.g. at a terminal position).

**Fig. 2:**
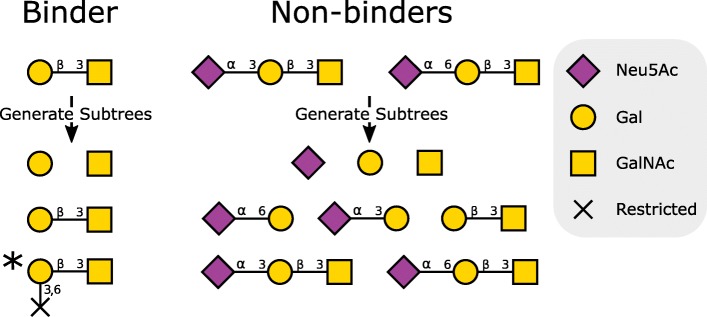
Addition of restricted linkage nodes improves selection of candidate motifs for glycan binding data. In this illustrative example, there is a single glycan (Gal *β*1-3GalNAc) capable of binding to a candidate lectin (e.g. PNA), while sialylation of the galactose residue (Neu5Ac *α*2-3Gal *β*1-3GalNAc and Neu5Ac *α*2-6Gal *β*1-3GalNAc) restricts binding. Generation of subtrees from these three glycans yields a set of potential motifs that could be used to discriminate between binders and non-binders. Note that one of these subtrees contains a ’restricted linkage’ node, to indicate the absence of a connection at positions 3 and 6 on the terminal galactose; there are connections at these positions within the non-binding set. This restricted linkage node is indicated by an X. Without consideration of restricted linkage nodes, there are no subtrees that are unique to the binding set. However, with addition of restricted linkage nodes, there is a single subtree from the binding set that adequately discriminates between binding and non-binding glycans. This candidate motif is marked with an asterisk. All glycan motif structures are shown in SNFG [51], modified with restricted linkages. Each restricted linkage, with corresponding carbon numbers, terminates in a cross in place of a residue symbol, according to the key

As a motivating example, we consider the peanut lectin (PNA), which binds to the T antigen disaccharide (terminal Gal *β*1-3GalNAc). This lectin does not bind when the disaccharide is sialylated on the galactose residue, as in the case of the sialyl T antigen (Neu5Ac *α*2-3Gal *β*1-3GalNAc). A standard motif finding approach has difficulty identifying a subtree which is present in Gal *β*1-3GalNAc but not its sialylated form (Fig. 2). Addition of restricted linkage nodes to indicate the absence of a connection at particular backbone positions allows easy discrimination between sialylated and asialylated forms of the T antigen disaccharide.

### Frequent subtree mining to generate a pool of possible motifs

Each glycan within a microarray was represented as a directed graph, with additional restricted linkage nodes to indicate lack of a connection at a particular backbone position. To minimise computational complexity, if several restricted linkage nodes are present on a single residue, these were merged into a single restricted linkage node whose edge value contains all empty connection positions (e.g. Fig. 2). Using a frequent subtree mining approach, we then generated all possible subtrees that meet a minimum support threshold of 5% for a given set of glycans. In the context of frequent subtree mining, the support for a particular subtree refers to the overall percentage of graphs which contain that subtree. Accordingly, the minimum support threshold is the threshold above which a subtree will be considered to be frequent. While a number of algorithms exist to extract frequent subtrees from a set of graphs, gSpan is one approach that is both efficient and deterministic [20]. We have used an implentation of gSpan called gBolt that is both faster and more memory efficient compared to the original gSpan implementation (https://github.com/Jokeren/gBolt) [21]. During method development, it was noted that some motifs occured at high frequencies within the positive binding set, but below the minimum support threshold of 5% used for selecting subtrees from the entire set of glycans. As such, we also selected additional frequent subtrees from the positive binding set, using a relatively high minimum support threshold of 40%. A higher threshold is used when selecting frequent subtrees from the positive binding set as there tends to be more commonality between glycans within the positive binding set. It is noted that these thresholds have been chosen as a tradeoff between computational run-time and ability to retrieve low-frequency motifs. These thresholds may need to be optimised for other glycan microarray systems, however the above thresholds were used for all microarrays analysed in this manuscript.

### Motif identification

#### Feature selection using mRMR

Generation of frequent subtrees yielded a large set of subtrees (e.g. there are 4121 subtrees for CFG microarray version 5.0 at a 5% minimum support threshold). To reduce this to a small set of distinguishing motifs, we performed feature selection using a state-of-the-art algorithm termed minimum-redundancy, maximum-relevance (mRMR) [22]. The mRMR algorithm selects features which both maximise mutual information between class labels and selected features (maximum relevance), while also minimising mutual information between selected features (minimum redundancy). We have used an implementation of mRMR called fast-mRMR [23], accessed at https://github.com/sramirez/fast-mRMR. For mRMR, input features were derived from the frequent subtrees identified in the previous step (i.e. each subtree is an individual feature). The mRMR algorithm also uses the class labels from the training dataset to determine the final set of selected features. mRMR is a filter method for feature selection, and hence requires the user to select the total number of features to be extracted. For this work, a total of 10 features were selected using fast-mRMR, as this was considered an adequate number of features to describe glycan binding properties.

#### Motif augmentation

Following generation of candidate motifs using mRMR, the set of potential motifs was extended by adding new motifs that include information on parent edge type (i.e. the anomeric descriptors at the reducing end of the motif, either *α* or *β*). This was motivated by the observation that some glycan binding motifs are dependent on the type of glycosidic linkage present on the reducing end of the motif (e.g. ABA lectin recognises Gal *β*1-3GalNAc *α*). Importantly, the residue at the reducing end of the motif may or may not include the anomeric desciptor, depending on the motif in question. For example, a motif may specify that a particular residue is *α*-linked but that the linked residue does not determine binding. Likewise, a parent edge type that is either *α*- or *β*-linked (*α*/ *β*) simply indicates that a linked residue is required for binding, and that the glycosidic linkage does not determine binding. Following generation of these new features, another round of feature selection with mRMR was performed using both the original set of motifs and motifs with information on anomer type at the reducing end of the motif. This process allows identification of motifs with finer specificity.

#### Feature selection with logistic regression with L1 regularisation

As the mRMR algorithm selects a defined number of features, it is possible that some of these selected features are uninformative. We therefore performed an additional round of feature selection using logistic regression with L1 regularisation, which encourages sparsity in model coefficients. Additionally, because of the imbalanced nature of the dataset, we incorporated class weights proportional to the number of samples in each class. The final cost function to be minimised is:

$$ {}cost(\mathbf{w}) = -C\sum_{n=1}^{N}\{\alpha_{1}t_{n}\ln{y_{n}} + \alpha_{0}(1-t_{n})\ln{(1 - y_{n})}\} + \lVert{\mathbf{w}}\rVert_{1} $$

where *α*_0_ and *α*_1_ are class weights inversely proportional to the number of samples in each class, *t*_*n*_=1/(1+ exp(−**w**^*T*^**x**_*n*_)), **w** is the vector of model coefficients, *y*_*n*_∈(0,1), and **x**_*n*_ is the feature vector for sample *n*. The regularisation parameter *C* was selected using 5-fold cross validation, with *C* selected to maximise average Matthews Correlation Coefficient (MCC) across all folds. *C* was selected from a set of 100 evenly spaced (in the log domain) values between 10^−4^ and 10^4^. Features with non-zero coefficients were selected for inclusion in a final logistic regression model with L2 regularisation.

Additionally, to remove features with perfect colinearity, we calculated variance inflation factors (VIF) for each feature in the model. Features with infinite VIFs were removed in a step-wise manner, recalculating VIFs for remaining features at each step.

### Logistic regression model

For classification of glycan binding, we chose a logistic regression model, both to minimise the likelihood of overfitting and to enable straightforward interpretation of model coefficients (as compared to a neural network, for example). A logistic regression model was trained using the final set of features, with a small amount of L2 regularisation and class weights inversely proportional to the number of samples in each class, with a cost function:

$$ {}cost(\mathbf{w}) = -C\sum_{n=1}^{N}\{\alpha_{1}t_{n}\ln{y_{n}} + \alpha_{0}(1-t_{n})\ln{(1 - y_{n})}\} + \lVert{\mathbf{w}}\rVert_{2} $$

where C = 100.

Model performance was assessed using the test set. Receiver operating characteristic (ROC) curves were generated for each glycan microarray (for both test and training sets), and final model performance assessed using the area under the curve (AUC) value.

### Software and analysis tools

The Python PyParsing package was used to build a parser to turn CFG glycan strings into a graph format. All graph manipulation was performed using the Python NetworkX package. Additional Python libraries used include Scikit-learn, Pandas, Numpy and Scipy. The Statsmodels Python library was used for calculation of variance inflation factors [24]. All frequent subtree mining was performed with gBolt. All code and methods are available at https://github.com/andrewguy/CCARL.

### Method comparison

To further validate our method, we compared components of our pipeline with pre-existing methods. The first aspect of our pipeline involves determining positive and negative binding glycans from a microarray experiment. We compared the MAD-based method used here for distinguishing binding from non-binding glycans with the ‘Universal Threshold’ described by Wang et al. [25], and the *z*-score (*p*-value <0.15) method incorporated into the GLYMMR algorithm [14]. All available concentrations in the CFG database were collated for each of the lectins examined, as both the methods of Wang et al. and Cholleti et al. use information from a range of lectin concentrations.

Secondly, we compared our motif identification pipeline to existing motif identification tools, including GLYMMR [14], the Glycan Miner Tool [13] hosted on RINGS (https://rings.glycoinfo.org/), and MotifFinder [18]. We assessed GLYMMR at a range of minimum support thresholds (20%, 30%, 40% and 50%), reporting both the mean AUC value across all thresholds and the best AUC for each sample. All other parameters were set to the defaults described in Cholleti et al. [14] (m = 3, no negative threshold, no additional filtering of substructures). The Glycan Miner Tool was run with parameters alpha = 0.8 and a minimum support of 20%. Motifs obtained from both GLYMMR and the Glycan Miner Tool were used to generate a classification model using L2-regularised logistic regression (using the same parameters as those used for the CCARL method). Performance was assessed using stratified 5-fold cross validation, with mean AUC values calculated across all folds. To ensure consistent evaluation between tools, we used the same assignment of positive and negative binding glycans for all tools (using the MAD-based method described earlier).

For a comparison to MotifFinder, we used the training datasets generated previously to generate contiguous motifs (one to four nodes in length) with MotifFinder. We then built a lectin model with the MotifFinder tool using the training dataset, before predicting glycan RFU values on the test dataset. Predicted RFU values were then used to calculate AUC values for MotifFinder. Note that only a single test-training split was used to assess MotifFinder as this tool does not support programmatic access.

## Results

To assess the performance of our motif identification and glycan classification method, we selected a number of plant- and fungi-derived lectins with well characterised binding motifs that are commonly used in experimental settings. These include peanut agglutinin (PNA), concanavalin A (Con A) and *Ricinus communis* agglutinin I (RCA I/RCA_120_). We also selected three examples relevant to host–pathogen interactions, namely haemagglutinins (HA) from two strains of influenza, and human DC-SIGN (see Table 1 for a full list). To ensure consistency between datasets and to maintain underlying data quality, we used glycan microarray data from experiments with Lara Mahal as the principal investigator [25] and lectins sourced from Vector Laboratories, wherever possible. As each lectin was typically analysed at a range of concentrations, we selected data from 10 *μ*g/ml of lectin, except when there was clearly better separation between positive and negative classes at a different concentration (as judged from a histogram of RFUs), or when experimental data was not available at 10 *μ*g/ml.

**Table 1:** Classification performance and identified motifs for common lectins

Lectin	Conc. (*μ*g/ml)	AUC (Validation)	AUC (Train)	Top Motif*	
*Agaricus bisporus* agglutinin (ABA)	100	0.934 (0.034)	0.947 (0.006)	(*3,4,6)GlcNAc *α*	
Concanavalin A (Con A)	10	0.971 (0.031)	0.982 (0.015)	Man *α*1-3(*2,4)Man	
*Dolichos biflorus* agglutinin (DBA)	100	0.839 (0.069)	0.897 (0.042)	(*3,4,6)GalNAc	
Human DC-SIGN tetramer	200	0.841 (0.062)	0.955 (0.026)	Man *α*1-3(Man *α*1-6)(*2,4)Man *α*	
*Griffonia simplicifolia* Lectin I isolectin B_4_ (GSL I-B_4_)	10	0.867 (0.061)	0.953 (0.014)	(*2,3,4,6)Gal *α*1-3Gal *β*	
Influenza hemagglutinin (HA) (A/Puerto Rico/8/34) (H1N1)	200	0.913 (0.105)	0.973 (0.023)	(*8,9)Neu5Ac *α*	
Influenza HA (A/harbor seal/Massachusetts/1/2011) (H3N8)	200	0.959 (0.028)	0.958 (0.007)	(*8,9)Neu5Ac *α*2-3(*2,4,6)Gal	
Jacalin	1	0.882 (0.055)	0.896 (0.009)	(*4,6)GalNAc *α*/ *β*	
*Lens culinaris* agglutinin (LCA)	10	0.964 (0.032)	0.976 (0.008)	Man *α*1-3Man *α*	
*Maackia amurensis* lectin I (MAL-I)	10	0.833 (0.035)	0.848 (0.053)	(*2,4,6)Gal *β*1-4(*3,6)GlcNAc *α*/ *β*	
*Maackia amurensis* lectin II (MAL-II)	10	0.718 (0.078)	0.814 (0.074)	Gal *β*1-3GalNAc *α*	
*Phaseolus vulgaris* erythroagglutinin (PHA-E)	10	0.959 (0.018)	0.975 (0.009)	(*2,4,6)Gal *β*1-4(*3,6)GlcNAc *β*1-2Man *α*1-3(Man *α*1-6)Man	
*Phaseolus vulgaris* leucoagglutinin (PHA-L)	10	0.914 (0.126)	0.967 (0.030)	GlcNAc *β*1-6(*3,4)Man	
Peanut agglutinin (PNA)	10	0.914 (0.048)	0.943 (0.021)	(*2,3,4,6)Gal *β*1-3GalNAc	
*Pisum sativum* agglutinin (PSA)	10	0.890 (0.053)	0.929 (0.028)	Man *α*1-3(*2,4)Man	
*Ricinus communis* agglutinin I (RCA I/RCA_120_)	10	0.953 (0.026)	0.958 (0.008)	(*2,3,4,6)Gal *β*1-4(*3,6)GlcNAc	
Soybean agglutinin (SBA)	10	0.875 (0.061)	0.938 (0.026)	(*3,4,6)GalNAc	
*Sambucus nigra* agglutinin (SNA)	10	0.950 (0.060)	0.979 (0.010)	Neu5Ac *α*2-6Gal *β*1-4GlcNAc	
*Ulex europaeus* agglutinin I (UEA I)	100	0.861 (0.049)	0.895 (0.042)	(*3)Fuc	
Wheat germ agglutinin (WGA)	1	0.882 (0.021)	0.901 (0.004)	GlcNAc *β*1-3Gal *β*1-4(*3,6)GlcNAc *β*1-3(*2,4,6)Gal *β*1-4(*3,6)GlcNAc	

Model performance was assessed using stratified 5-fold cross-validation, with Area Under the Curve (AUC) values calculated for both validation and training folds (shown as mean (s.d.)). The top motif is defined as the feature with the highest coefficient in the logistic regression classification model, and is shown for a single test/training split. Experimentally determined lectin specificities and associated citations are provided in Additional file 7

^*^Note: Motifs are written in a modified CFG linear text nomenclature. A set of parentheses with connection types preceded by an asterisk indicates restricted connection types for the following residue. For example, a GlcNAc motif with restricted connections on C3 and C4 is indicated by (*3,4)GlcNAc

### Identification of key binding motifs for PNA

**Fig. 3:**
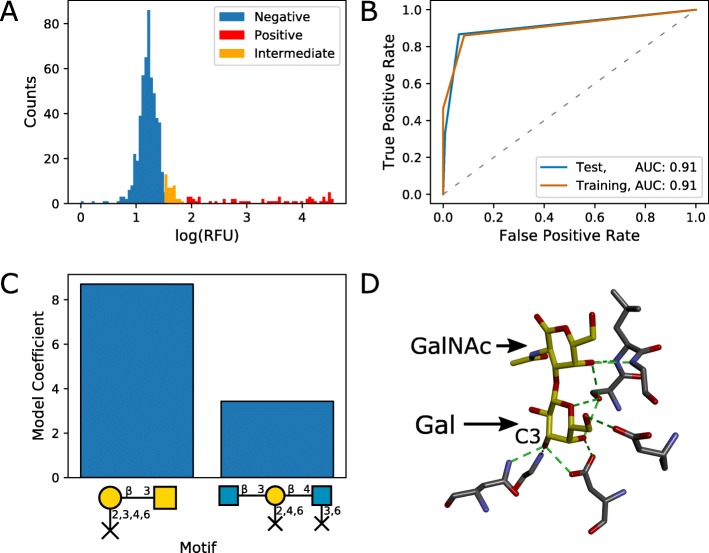
Predicted carbohydrate-binding motifs of PNA from CFG glycan microarray data. **a** Distribution of RFUs and classification of non-binding (blue), intermediate binding (orange), and binding glycans (red). **b** ROC curves for the test (n=143) and training (n=428) sets. The ratio of negative to positive samples was 9.0. **c** Logistic regression coefficients for identified motifs. **d** The intermolecular hydrogen bonding interactions (shown in green) between the T antigen (carbon backbone shown in yellow) and the carbohydrate-binding domain of peanut agglutinin (PNA) (carbon backbones shown in grey). Carbon 3 of the Gal monomer is labelled to indicate where the sialic acid is linked in the sialyl T antigen. Reproduced from an X-ray crystal structure at 2.5 Å resolution available at the PDB (PDB: 2TEP) [28]. See Additional file 1 for a detailed notation key

Peanut agglutinin is a legume-derived lectin used in cell-based assays [26]. Following feature selection by mRMR and sparsity-promoting logistic regression with L1 regularisation, two motifs were selected for inclusion in a final model for PNA. The motif with the highest coefficient in the final logistic regression model was a Gal *β*1-3GalNAc motif, with restricted linkages on the non-reducing galactose residue (Fig. 3c). This agrees well with published reports of PNA binding specificity [27]. The Gal *β*1-3GalNAc motif is otherwise known as the tumour-associated (T) antigen, and its galactose residue is commonly sialylated to yield the sialyl T antigen. The motif retrieved in our model would restrict sialylation at the terminal galactose residue, which is supported by crystal structures of PNA binding to the T antigen [28]. The T antigen was also returned by the Glycan Miner Tool, but not by GLYMMR, and neither specifies the restricted linkage at the terminal galactose residue (Additional file 8). Within the bound structure, the terminal galactose residue is heavily involved in interactions with amino acid residues in the binding site of PNA (Fig. 3d). The final logistic regression model gave good classification performance, with AUC values of 0.908 and 0.909 for the training and test sets, respectively (Fig. 3b).

We note here that while interpretation of coefficients from a logistic regression model is relatively straightforward when there is little correlation between features, there are additional complexities to consider when features are highly correlated. For uncorrelated features, model coefficients can be interpreted as the change in the log-odds of glycan binding when that particular feature/motif is present. However, when features are highly correlated, there can be significant interplay between coefficients for correlated features. Therefore, interpretation of model coefficients for highly correlated motifs should be treated with a degree of caution. To assess the level of collinearity for each feature, we have calculated variance inflation factors for each set of predictive motifs (Additional file 3). Motifs with high variance inflation factors should be treated with caution — while these may still be important motifs, the model coefficient values may not be indicative of the true importance of that feature as a binding motif.

### Identification of key binding motifs for Con A

**Fig. 4:**
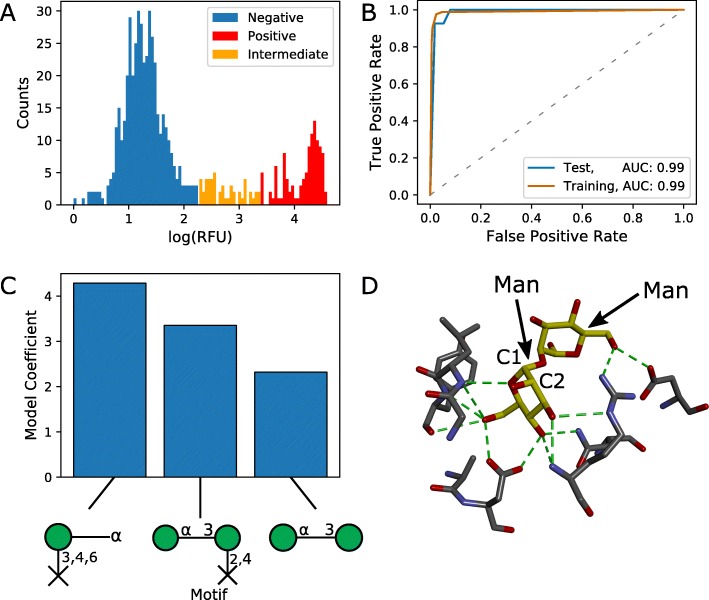
Predicted carbohydrate-binding motifs of Con A from CFG glycan microarray data. **a** Distribution of RFUs and classification of non-binding (blue), intermediate binding (orange), and binding glycans (red). **b** ROC curves for the test (n=141) and training (n=421) sets. The ratio of negative to positive samples was 4.1. **c** Logistic regression coefficients for identified motifs. **d** The intermolecular hydrogen bonding interactions (shown in green) between 2 *α*-mannobiose (carbon backbone shown in yellow) and the carbohydrate-binding domain of Concanavalin A (carbon backbones shown in grey). Reproduced from an X-ray crystal structure at 1.2 Å resolution available at the Protein Data Bank (PDB: 1I3H) [52]. See Additional file 1 for a detailed notation key

Con A is another widely available L-type lectin and is used extensively in lectin affinity chromatography [29]. Using glycan microarray data for Con A, we identified terminal *α*-linked mannose residues as the motif with the second highest model coefficient (Fig. 4c). This motif does, however, allow linkages from the carbon 2 of the mannose residue, which describes the non-branching linkages of mannose residues in oligomannose *N*-glycans. Interestingly, terminal mannose was not specified as a motif by either GLYMMR or the Glycan Miner Tool (Additional file 8), which fail to describe Con A’s high affinity for oligomannose *N*-glycans. A co-crystallised structure of Con A with a mannose disaccharide (Fig. 4d) explains Con A’s affinity for *α*-linked mannose residues, in agreement with the motifs identified by our approach. The other identified motifs describe the Man *α*1-3 arm of the *N*-glycan core. This is in agreement with the reported broad selectivity of Con A for *N*-glycans [30]. There is also crystallographic evidence of Con A binding to the pentasaccharide core of *N*-glycans, although this suggests a higher affinity for the Man *α*1-6 arm [31]. While both GLYMMR and the Glycan Miner Tool captured the specificity of Con A for the *N*-glycan core, the motifs identified by these tools are larger, and don’t solely specify the mannose core as the main binding determinant. When using the motifs identified by CCARL as features for a logistic regression classifier, we observed high AUC values of 0.989 and 0.987 for the training and test sets, respectively (Fig. 4b).

### Identification of key binding motifs for RCA I

**Fig. 5:**
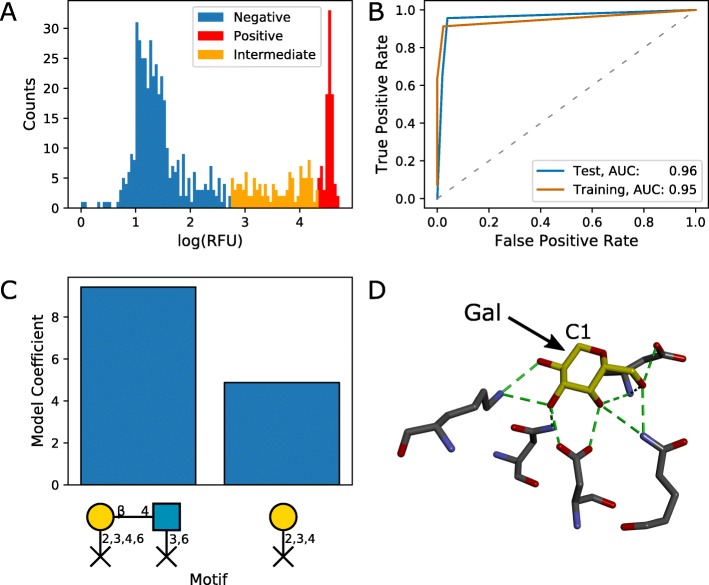
Predicted carbohydrate-binding motifs of RCA I from CFG glycan microarray data. **a** Distribution of RFUs and classification of non-binding (blue), intermediate binding (orange), and binding glycans (red). **b** ROC curves for the test (n=125) and training (n=372) sets. The ratio of negative to positive samples was 4.4. **c** Logistic regression coefficients for identified motifs. **d** The intermolecular hydrogen bonding interactions (shown in green) between *β*-galactose (carbon backbone shown in yellow) and the carbohydrate-binding domain of the B chain of ricin (carbon backbones shown in grey). Reproduced from an X-ray crystal structure at 2.5 Å resolution available at the PDB (PDB: 3RTI) [39]. See Additional file 1 for a detailed notation key

RCA I is an R-type lectin often used in histochemical staining [32]. Using CFG glycan microarray data, glycan structures terminating in *β*-linked galactose residues were predicted as potential binding motifs for RCA I (Fig. 5c). These motifs are consistent with the published selectivity of RCA I from chromatographic studies, including a preference for Gal *β*1-4GlcNAc over Gal *β*1-3GlcNAc and reduced affinities for galactose residues with linkages from the 3-, 4-, or 6-OH, and for *N*-acetylglucosamine residues with 3-OH linkages [33]. While linkages from the 6-OH reduce the binding affinity of RCA I, the second motif listed in Fig. 5c does not preclude these as binding glycans. This is consistent with the observation that RCA I tolerates the addition of an *α*2-6-linked sialic acid to the galactose residue [34]. This affinity was also captured by the Glycan Miner Tool, but not by GLYMMR (Additional file 8). When using these motifs for a logistic regression classifier, we observed high AUC values of 0.952 and 0.962 for the training and test sets, respectively (Fig. 5b), further supporting the validity of the identified motifs. Although there are no crystal structures available for RCA I on the PDB, the carbohydrate-binding B chain of heterotetrameric RCA I shares a high sequence homology with that of the toxin ricin (RCA II or RCA_60_), which also derives from the castor bean (*Ricinus communis*) [35]. As such, the co-crystallised structure for the lectin chain of ricin also supports *β*-galactose as a binding determinant (Fig. 5d).

### Binding motifs identified for haemagglutinins from different strains of influenza

**Fig. 6:**
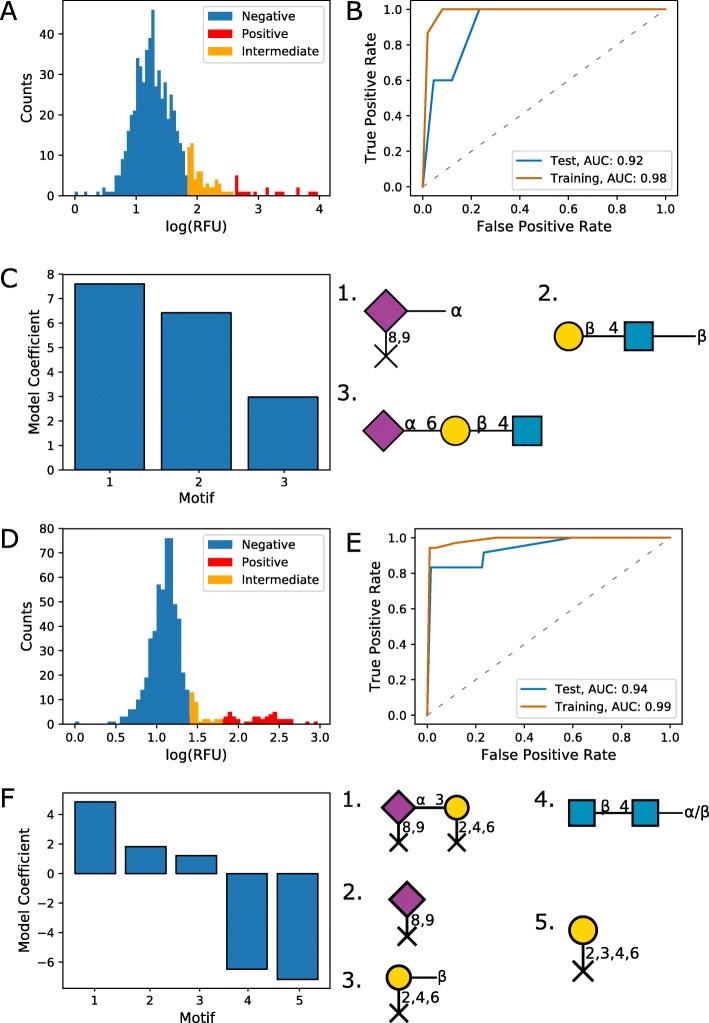
Predicted carbohydrate-binding motifs of two haemagglutinins from a human and an avian strain of influenza from CFG glycan microarray data. **a** Distribution of RFUs and classification of non-binding (blue), intermediate binding (orange), and binding glycans (red) for A/Puerto Rico/8/34 (H1N1) HA. **b** ROC curves for the test (n=138) and training (n=412) sets for A/Puerto Rico/8/34 (H1N1) HA. The ratio of negative to positive samples was 26.5. **c** Logistic regression coefficients for identified motifs for A/Puerto Rico/8/34 (H1N1) HA. **d** Distribution of RFUs and classification of non-binding (blue), intermediate binding (orange), and binding glycans (red) for A/harbor seal/Massachusetts/1/2011 (H3N8) HA. **e** ROC curves for the test (n=145) and training (n=433) sets for A/harbor seal/Massachusetts/1/2011 (H3N8) HA. The ratio of negative to positive samples was 11.4. **f** Logistic regression coefficients for identified motifs for A/harbor seal/Massachusetts/1/2011 (H3N8) HA. See Additional file 1 for a detailed notation key

Lectins are commonly found on the surfaces of microbes and are involved in host–pathogen interactions. As an example of a lectin that does not derive from legumes and is relevant to a human disease, we analysed glycan micoarray data from influenza haemagglutinins. The specificity of these haemagglutinins for *α*2-6-linked sialic acid residues, or *α*2-3-linked in the case of non-human strains [36], is well characterised and is reflected in the motifs identified by our pipeline (Fig. 6c, f). Accordingly, *α*2-6-linked (Fig. 6c) and *α*2-3-linked (Fig. 6f) sialic acid were identified as top motifs for the haemagglutinins from a human strain and an avian strain, respectively. However, Neu5Ac *α*2-6Gal *α*1-4GlcNAc was ranked as the third motif for the human strain. This highlights the importance of human synthesis of the top motifs in gaining a cohesive understanding of binding specificities. It is noted that classifier performance is not as good as that of Con A and RCA I, with test set AUC values of 0.912 and 0.945 for HA from human and avian strains, respectively (Fig. 6b, e). This may be partly due to the smaller number of positive binding glycans within the human HA data, with only 5 positive binders in the test set.

### Evaluation of method performance over a wide range of glycan microarrays

**Fig. 7:**
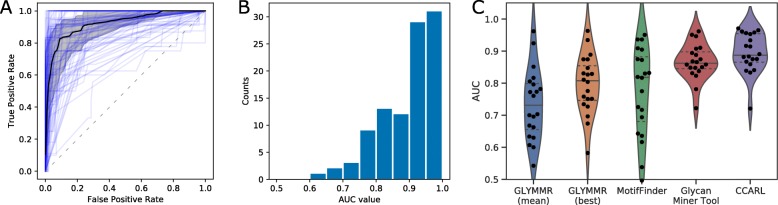
Classification performance across a range of different lectins. **a** Receiver-operator characteristic (ROC) curves across a number of different glycan microarray experiments. Individual ROC curves are shown in light blue. The median ROC curve is shown in black, with shading representing 25th-75th percentiles. The dashed line indicates an uninformative (random) classifier. **b** Area Under the Curve (AUC) values for all glycan microarray experiments examined. See Table 1 and Additional file 5 for a full list of lectins examined. **c** Classification performance of CCARL compared to existing glycan motif tools. Area Under the Curve (AUC) values were calculated across a number of different glycan microarray experiments using stratified 5-fold cross-validation (with the exception of MotifFinder, which was evaluated using a single fold). Motifs were extracted using GLYMMR, MotifFinder, the Glycan Miner Tool and CCARL, and assessed using a logistic regression model (with the exception of MotifFinder, which outputs predicted RFU values). Motifs from GLYMMR were extracted at several minimum support thresholds, and both the mean AUC value and best AUC value reported for each microarray experiment. Median and interquartile range are indicated by solid and dashed grey lines respectively

To assess the performance of this pipeline over a large set of glycan-binding proteins, we compiled a list of lectins that are commonly used in an experimental setting (Table 1, see Additional file 7 for known lectin specificities). We assessed model performance using stratified 5-fold cross-validation, calculating average Area Under ROC curves (AUC) across all iterations. Considerable variation in the performance of this pipeline between different glycan microarrays was observed, which is to be expected given the diverse range of binding modes and specificities between different lectins. Performance varied between close to perfect (e.g. a mean AUC of 0.97 for Con A) through to relatively poor (e.g. a mean AUC of 0.72 for MAL-II), although good classification performance was observed for the majority of lectins examined. Over all lectins examined, the median AUC value was 0.887 (IQR = 0.865–0.954) (Fig. 7a, b). The full list of motifs and associated model coefficients is supplied in Additional file 3.

### Comparison to other methods for motif identification from glycan microarray data

We compared the predictive performance of our pipeline to that of two other frequent subtree mining tools: the Glycan Miner Tool [13] hosted on RINGS (https://rings.glycoinfo.org/), and the GLYMMR algorithm [14] previously hosted on GlycoPattern (not available at the time of writing) (Fig. 7c, Table 2). The GLYMMR algorithm employs a frequent subtree mining approach, with some additional filtering to select motifs that are enriched within the positive binding set of glycans. GLYMMR requires the user to set a number of parameters, including a threshold parameter which is equivalent to a minimum support threshold. With the datasets we used for this comparison, we were unable to find a single minimum support threshold that yielded a similar number of motifs for all data—with a minimum support threshold of 20%, the number of returned motifs ranged from one to several thousand. As such, we assessed GLYMMR at a range of minimum support thresholds (20%, 30%, 40% and 50%) and for each sample report both the average AUC value across all thresholds as well as the AUC for the best threshold. The Glycan Miner Tool employs an alpha-closed frequent subtree mining approach, and has two user-defined parameters, alpha and minimum support. For this analysis, we chose alpha = 0.8 and a minimum support of 20%. These parameters were chosen as they yielded between 5 and 25 motifs across the range of glycan arrays tested, similar to the approach described in Ichimiya et al. [15]. A classification model (L2-regularised logistic regression) was built using the motifs obtained from both GLYMMR and the Glycan Miner Tool and performance was assessed using the same stratified 5-fold cross validation approach outlined previously. The motifs generated by both GLYMMR and the Glycan Miner Tool are available in Additional file 8. The classifier built using motifs from the Glycan Miner Tool had a median AUC of 0.862 (IQR = 0.845–0.898). Similarly, the motifs generated using the GLYMMR tool yielded a median AUC of 0.807 (IQR = 0.747–0.854) when selecting the best AUC across all minimum support thresholds and a median AUC of 0.732 (IQR = 0.655–0.798) when taking the mean AUC across all minimum support thresholds. Classification of glycan binding with CCARL was compared to MotifFinder, another tool for the prediction of glycan binding [18]. MotifFinder had a median AUC of 0.818 (IQR = 0.681–0.882). We note that MotifFinder requires the use of a prebuilt library of motifs, making the detection of novel/unusual motifs difficult, which may explain the poor performance of MotifFinder on some datasets. Overall, the CCARL method presented here has improved performance compared to GLYMMR, the Glycan Miner Tool and MotifFinder (Fig. 7c).

**Table 2:** Comparison of classifier performance across different motif generation tools

Lectin	GLYMMR(mean)	GLYMMR(best)	Glycan Miner Tool	MotifFinder	CCARL
*Agaricus bisporus* agglutinin (ABA)	0.607 (0.151)	0.776 (0.088)	0.888 (0.067)	0.905	**0.934 (0.034)**
Concanavalin A (Con A)	0.760 (0.083)	0.875 (0.048)	0.951 (0.042)	0.937	**0.971 (0.031)**
*Dolichos biflorus* agglutinin (DBA)	0.630 (0.098)	0.674 (0.126)	0.722 (0.083)	**0.936**	0.839 (0.069)
Human DC-SIGN tetramer	0.634 (0.132)	0.727 (0.125)	0.823 (0.130)	0.538	**0.841 (0.062)**
*Griffonia simplicifolia* Lectin I isolectin B_4_ (GSL I-B_4_)	0.773 (0.103)	0.847 (0.086)	**0.875 (0.066)**	**0.875**	0.867 (0.061)
Influenza hemagglutinin (HA) (A/Puerto Rico/8/34) (H1N1)	0.851 (0.140)	0.889 (0.103)	0.838 (0.144)	0.643	**0.917 (0.104)**
Influenza HA (A/harbor seal/Massachusetts/1/2011) (H3N8)	0.925 (0.059)	0.935 (0.034)	0.947 (0.021)	0.717	**0.958 (0.028)**
Jacalin	0.782 (0.061)	0.804 (0.050)	0.848 (0.026)	0.726	**0.882 (0.055)**
*Lens culinaris* agglutinin (LCA)	0.772 (0.092)	0.811 (0.083)	0.908 (0.083)	0.832	**0.956 (0.037)**
*Maackia amurensis* lectin I (MAL-I)	0.700 (0.054)	0.758 (0.057)	0.868 (0.050)	**0.873**	0.833 (0.035)
*Maackia amurensis* lectin II (MAL-II)	0.600 (0.162)	0.827 (0.056)	**0.850 (0.091)**	0.830	0.721 (0.073)
*Phaseolus vulgaris* erythroagglutinin (PHA-E)	0.817 (0.061)	0.875 (0.044)	0.910 (0.016)	0.496	**0.965 (0.021)**
*Phaseolus vulgaris* leucoagglutinin (PHA-L)	0.805 (0.095)	0.829 (0.089)	0.858 (0.110)	0.636	**0.875 (0.132)**
Peanut agglutinin (PNA)	0.668 (0.116)	0.751 (0.133)	0.894 (0.041)	0.617	**0.914 (0.048)**
*Pisum sativum* agglutinin (PSA)	0.796 (0.070)	0.830 (0.050)	0.858 (0.064)	0.694	**0.891 (0.053)**
*Ricinus communis* agglutinin I (RCA I/RCA_120_)	0.696 (0.053)	0.751 (0.032)	0.848 (0.034)	0.909	**0.953 (0.026)**
Soybean agglutinin (SBA)	0.542 (0.061)	0.582 (0.049)	0.781 (0.046)	0.775	**0.875 (0.061)**
*Sambucus nigra* agglutinin (SNA)	0.962 (0.051)	**0.963 (0.057)**	0.962 (0.050)	0.820	0.961 (0.059)
*Ulex europaeus* agglutinin I (UEA I)	0.703 (0.099)	0.734 (0.057)	0.866 (0.023)	**0.951**	0.859 (0.047)
Wheat germ agglutinin (WGA)	0.663 (0.048)	0.697 (0.055)	0.831 (0.034)	0.817	**0.883 (0.021)**

Model performance was assessed using stratified 5-fold cross-validation, with mean Area Under the Curve (AUC) values calculated across all validation folds (shown as mean (s.d.)). The best performing tool for each sample is highlighted in bold. Note the MotifFinder tool was evaluated with a single test-train split due to difficulty automating this tool. GLYMMR was evaluated across a range of minimum support thresholds, with AUC values reported for the best threshold as well as mean AUC values across all thresholds

We also compared different methods of thresholding to categorise binding vs. non-binding glycans. Overall, our MAD-based method for distinguishing binding from non-binding glycans proved to be less conservative than either the Universal Threshold described by Wang et al. [25] or *z*-score method incorporated into the GLYMMR algorithm [14], capturing larger positive binding sets of glycans (see Additional file 4).

## Discussion

In this work, we have developed a classification tool for glycan microarray data, which can also be used to suggest likely binding motifs. This tool employs a frequent subtree mining approach, and includes information on ‘restricted linkages’, allowing specific identification of terminal motifs that can only bind if present at the non-reducing end of glycans. We have assessed this tool across several commonly used lectins, using publicly available data from the CFG. Overall, this tool had good classification performance for a range of lectins and was able to identify key motifs for each lectin. These motifs are mostly consistent with reported binding selectivities. However, our results further challenge the often incorrectly cited exclusive affinity of MAL II for *α*2-3-linked sialic acids [37] (see Additional file 3). However, these AUC values are low (0.758 and 0.859 for the test and training sets, respectively), and so more investigation would be required to predict actual binding determinants of MAL II. Additionally, we were unable to capture the reported selectivity of PHA-E for asialylated terminal galactose on the Man *α*1-6 arm of bisected *N*-glycans [38], and nor was this apparent upon manual examination of the positive binding set.

One challenging aspect of dealing with large-scale analysis of glycan microarrays is the automatic assignment of positive binding glycans, as both the location and spread of background RFUs can vary considerably between different analytes and their concentrations. This made it difficult to assign a single RFU as a defining positive binding threshold. While we explored several existing approaches for assignment of positive binders [14, 25], we ultimately used a technique based on modified *z*-scores, derived from Median Absolute Deviation (MAD) scores. MAD scores are robust to outliers and are hence resilient to a significant population of positive binders, unlike standard parametric approaches, such as *z*-scores based on estimates of standard deviation. The use of a MAD-based method is supported by the excellent classification performance obtained across several glycan microarrays (e.g. an AUC of 0.99 for Con A), which would not be expected with an inappropriate threshold for identification of positive binders. Additionally, MAD performed favourably compared with both the Universal Threshold and the unmodified *z*-scores incorporated into GLYMMR, capturing larger positive binding sets. This is particularly advantageous in mining for secondary motifs, whose RFUs can be dwarfed by highly homologous primary motifs. While we did not explore MAD-based assignment of positive binders with other glycan microarray platforms, we expect this technique to have broad applicability outside of the CFG microarray data. We also note that the intermediate binding set presents another opportunity to mine for secondary motifs. However, we did not incorporate the intermediate binding set into these analyses, so as to avoid Type I errors. We leave it to the user’s discretion as to whether the intermediate binding set should be considered in each analysis, but caution that measures should be taken to prevent Type I errors, such as the use of higher thresholds.

One of the major contributions of this work, in comparison to other frequent subtree mining approaches for motif identification, is the addition of restricted linkage nodes. These indicate the absence of a connection at a particular position within a motif. This enables identification of terminal residues as potential motifs. For example, we identified the T antigen (Gal *β*1-3GalNAc) as a candidate binding motif for peanut agglutinin (PNA), excluding any forms with additional residues connected to the galactose residue (Fig. 3). In support of this observation, PNA has been shown experimentally to bind to terminal T antigen but not to sialyl T antigen (Neu5Ac *α*2-3Gal *β*1-3GalNAc) [27]. Additionally, the binding mode for T antigen to PNA, as observed by X-ray crystallography, would exclude sialylation of the non-reducing galactose. The utility of restricted linkages was also demonstrated by the identification of terminal *β*-linked galactose as a potential binding motif for RCA I, which is supported by crystal structures of the highly homologous ricin B chain [35, 39] (Fig. 5).

Previous work by Klamer et al. introduced the concept of a ‘free’ modifier with respect to glycan motifs [18] and incorporated this into MotifFinder. MotifFinder does not perform frequent subtree mining, and is primarily used with a library of motifs, which may explain the overall performance gap compared to our pipeline. While in some cases the use of a prebuilt library may perform better than frequent subtree mining, a frequent subtree mining approach is likely to be more suited to identification of unusual or novel motifs from glycan microarray data. We also compared CCARL to other existing tools for motif identification, with CCARL performing better than both GLYMMR and Glycan Miner Tool. The Glycan Miner Tool generally performed well, with only a small difference in median AUC values compared to CCARL. In general, the motifs returned by the Glycan Miner Tool were similar to those returned by CCARL, although often larger, whereas the motif returned by CCARL are smaller and seem to capture the core binding determinant. For example, the motifs returned by CCARL for Con A capture the specificity for core mannose residues, whereas those returned by the Glycan Miner Tool also include residues surrounding the mannose core (Additional file 8). While both GLYMMR and Glycan Miner Tool employ a frequent subtree mining approach, neither tool considers restricted linkages, and the improved performance of CCARL over these other tools validates the inclusion of restricted linkages in motif mining tools.

One limitation of a subtree mining approach for motif detection is the limited ability to accurately detect structural constraints that may impact on glycan binding. These constraints include steric hindrance effects from other parts of the glycan structure or situations in which the potential motif is inaccessible for binding due to arrangement on a protein or microchip surface. When constructing glycan microarrays, various linkers are used to conjugate glycans to the surface of the microarray. On the CFG glycan microarrays, linkers are amino acids and amino-functionalised organic molecules, which allow covalent coupling to the *N*-hydroxysuccinimide-activated glass slides [10]. However, the Carbohydrate Microarray Facility of Imperial College London produces lipid-linked glycan microarrays, which better simulate in vivo binding interactions of glycolipids [40]. There is considerable evidence that linker type has an impact on recognition of motifs on glycan microarrays, with Grant et al. demonstrating that this can be explained by glycan orientation relative to the microchip surface, which can restrict protein binding to an otherwise complementary motif [41]. Similarly, motif location within the overall glycan can have a large impact on binding affinity. While we attempted to capture some of these phenomena with the inclusion of restricted linkages, there are likely to be other steric constraints that are not captured by this approach. For example, when applying our classifier to the ABA lectin, it was noted that many of the false positive binders included a bisecting *N*-acetylglucosamine residue from the mannosyl core. ABA recognises terminal *N*-acetylglucosamine residues as well as T antigen [42], which were both identified as motifs by our method (Table 1 and Additional file 3: Figure S1). It is likely that steric hindrance from the branches either side of a *N*-acetylglucosamine residue that bisects the trimannosyl core prevents binding of ABA to the residue. However, our classifier fails to distinguish non-bisecting, terminal *N*-acetylglucosamine residues from bisecting *N*-acetylglucosamine residues. Similarly, we identified core mannose residues as motifs for Con A binding; Con A is therefore predicted to bind to any *N*-glycan. However, more highly branched *N*-glycans (e.g. tetra-antennary) have been shown to restrict Con A binding to core mannose residues [43]. As such, a method accounting for the spatial environment of potential motifs may improve prediction performance, and this is a potential avenue for future work.

Glycan classification and motif identification tools can be used to extend the effective coverage of existing experimental glycan microarrays. While the number of glycans included in glycan microarrays has steadily increased over time, there are still many glycans not covered by existing microarrays; the number of glycans in the human glycome is estimated to be approximately 9,000 [44], while there are only 609 glycans in the most recent CFG glycan microarray. This highlights a potential role for classification tools (such as the one described in this paper) in predicting lectin binding to the large number of glycans not included in current microarrays. It is also important to consider the types of glycans included in a microarray. The CFG glycan microarrays are biased toward mammalian and, particularly, human structures, and so are less helpful for evaluating non-mammalian glycan ligands. In these settings, a glycan microarray customised for the organism of interest could be used for classifier training to ensure more accurate binding predictions [45, 46].

Ideally, any prediction of binding gained from glycan microarray experiments should be validated by other methods, such as affinity chromatography, X-ray crystallography, and in vivo assays [47]. However, the use of motif prediction tools can serve to narrow down the number of potential motifs that need to be investigated and validated with traditional wet-lab techniques. For example, Ichimiya et al. used the glycan miner tool available at the RINGS to search for novel binding determinants of influenza [15]. Although, the sulfated structures posited as determinants in this study were not captured as top motifs in our results, and we suggest more experimental evidence, such as crystallographic data or a customised glycan microarray, is required to verify these binding determinants.

While CCARL aids in the identification of glycan motifs, a manual interpretation of the top motifs is often still required to gain a complete understanding of predicted binding determinants. For example, the top motif identified for LCA is Man *α*1-3Man *α* (see Table 1 and Additional file 6: Figure S9), which may appear strange for a lectin reported to bind to core fucoses. However, closer inspection of the remaining top motifs reveals *α*1-6-linked core fucose as a key motif. This makes sense upon examination of the literature, which reports *α*-linked mannose oligosaccharides as the main binding determinant of LCA, and the addition of core fucoses to enhance binding [48]. Wholly manual interpretations of glycan microarray data have previously led to important discoveries. A custom microarray of glycans from human milk was used to discover Gal *β*1-3GlcNAc *β*1-3Gal *β*1-4Glc as a binding determinant of a neonatal strain of rotavirus [49], which was later validated as a co-crystallised protein structure [50]. However, manual identification of binding motifs becomes more difficult with a greater number and variety of glycans included on a microarray, making automated pipelines for identification of binding motifs and prediction of glycan binding essential.

## Conclusions

We present here an automated method for the identification of candidate motifs from glycan microarray data, which allows accurate classification of glycans with unknown binding behaviour. We have termed this approach ‘Carbohydrate Classification Accounting for Restricted Linkages’ (CCARL). This method extends frequent subtree mining approaches of glycan microarray data by allowing identification of terminal motifs, distinguishing these from otherwise identical motifs present elsewhere within glycan structures. Using a set of glycan microarray data from the CFG, we demonstrate that our classification pipeline successfully identifies binding motifs of well characterised lectins, in agreement with their published selectivities and with generally excellent classification performance. CCARL will aid in the identification of motifs from the ever-increasing number of glycan microarrays, supporting research to improve our understanding of human-, plant-, and pathogen-derived glycan-binding proteins.

## Supplementary information

**Additional file 1** Modified SNFG key. The Symbol Nomenclature for Glycans (SNFG) [51] was used for drawing all glycans, with the addition of a cross to indicate a restricted linkage node.

**Additional file 2** Representation of glycans as directed, labelled graphs. Glycans were represented as directed, labelled graphs, with nodes labelled with monosaccharide type, and edges labelled with connection type (i.e. anomer and linkage position). Additional nodes were added to represent the absence of a potential link, and termed restricted linkage nodes.

**Additional file 3** Motif identification for a range of glycan microarrays. Detailed motifs and classifier performance for the range of glycan microarrays presented in Table 1.

**Additional file 4** Comparison of MAD-based detection of positive binders to other methods for detecting positive binding glycans. Detection of positive binding glycans by median absolute deviation (MAD) compared to the *z*-score threshold employed by Cholleti et al. [14] and the ‘Universal Thresholding’ approach employed by Wang et al. [25]. For the *z*-score threshold, *z*-scores were calculated for the average RFU values across all concentrations (as outlined by Cholleti et al. [14]). For the Universal Threshold approach, only microarrays within a determined linear range (see Wang et al. [25] for further details) were used to determine positive binders. As the MAD-based thresholding approach is applied to each microarray separately, individual plots for each concentration are shown for MAD-based thresholding.

**Additional file 5** Details on lectins examined in this study. Glycan microarray data used in this study was obtained from the CFG. This file provides additional details to Table 1 on the datasets used throughout this work.

**Additional file 6** Training and test sets. CSV files for all training and test sets used for this manuscript.

**Additional file 7** Lectin specificities. Experimentally characterised lectin specificities for all lectins examined in this study.

**Additional file 8** Motifs from GLYMMR and glycan motif miner. Motifs extracted using GLYMMR and Glycan Miner Tool for a range of glycan microarray datasets.

## Data Availability

The glycan microarray datasets analysed in this study were obtained online at the Consortium for Functional Glycomics (http://www.functionalglycomics.org/) and are detailed in Additional file 5. All code and associated data for the Carbohydrate Classification Accounting for Restricted Linkages (CCARL) method is available at https://github.com/andrewguy/CCARL.

## Abbreviations

ABA: *Agaricus bisporus* agglutinin
AFP: *α*-fetoprotein
AUC: Area under the curve
CFG: Consortium for functional Glyomics
Con A: Concanavalin A
DBA: *Dolichos biflorus* agglutinin
GLYMMR: GlycanMotifMiner
GSL I B_4_: *Griffonia simplicifolia* Lectin I isolectin B_4_
HA: Haemagglutinin
LCA: *Lens culinaris* agglutinin
MAD: Median absolute deviation
MAL I: *Maackia amurensis* lectin II
MAL II: *Maackia amurensis* lectin I
MCAW: Multiple Carbohydrate Alignment with Weights
MCC: Matthews Correlation Coefficient
mRMR: Minimum redundancy, maximum relevance
PDB: Protein Data Bank
PHA-E: *Phaseolus vulgaris* erythroagglutinin
PHA-L: *Phaseolus vulgaris* leucoagglutinin
PNA: Peanut agglutinin
PSA: *Pisum sativum* agglutinin
RCA I: *Ricinus communis* agglutinin I
RFU: Relative fluorescence units
RINGS: Resource for Informatics of Glycomes at Soka
ROC: Receiver operating characteristic
SBA: Soybean agglutinin
SNA: *Sambucus nigra* agglutinin
SNFG: Symbol Nomenclature for Glycans
T antigen: Tumour-associated antigen
UEA I: *Ulex europaeus* agglutinin I
WGA: Wheat germ agglutinin

## References

[1] Hakomori S-I, Kannagi R (1983). Glycosphingolipids as tumor-associated and differentiation markers. J Natl Cancer Inst.

[2] Paszek MJ, DuFort CC, Rossier O, Bainer R, Mouw JK, Godula K, Hudak JE, Lakins JN, Wijekoon AC, Cassereau L, Rubashkin MG, Magbanua MJ, Thorn KS, Davidson MW, Rugo HS, Park JW, Hammer DA, Giannone G, Bertozzi CR, Weaver VM (2014). The cancer glycocalyx mechanically primes integrin-mediated growth and survival. Nature.

[3] Weis W, Brown JH, Cusack S, Paulson JC, Skehel JJ, Wiley DC (1988). Structure of the influenza virus haemagglutinin complexed with its receptor, sialic acid. Nature.

[4] East L, Isacke CM (2002). The mannose receptor family. Biochim Biophys Acta.

[5] Peumans WJ, Van Damme EJ (1995). Lectins as plant defense proteins. Plant Physiol.

[6] Sato Y, Nakata K, Kato Y, Shima M, Ishii N, Koji T, Taketa K, Endo Y, Nagataki S (1993). Early recognition of hepatocellular carcinoma based on altered profiles of alpha-fetoprotein. N Engl J Med.

[7] Noda K, Miyoshi E, Uozumi N, Yanagidani S, Ikeda Y, Gao C, Suzuki K, Yoshihara H, Yoshikawa K, Kawano K, Hayashi N, Hori M, Taniguchi N (1998). Gene expression of alpha1-6 fucosyltransferase in human hepatoma tissues: a possible implication for increased fucosylation of alpha-fetoprotein. Hepatology.

[8] Oswald DM, Cobb BA (2018). Emerging glycobiology tools: A renaissance in accessibility. Cell Immunol.

[9] Yamanishi Y, Bach F, Vert J-P (2007). Glycan classification with tree kernels. Bioinformatics.

[10] Blixt O, Head S, Mondala T, Scanlan C, Huflejt ME, Alvarez R, Bryan MC, Fazio F, Calarese D, Stevens J, Razi N, Stevens DJ, Skehel JJ, van Die I, Burton DR, Wilson IA, Cummings R, Bovin N, Wong C-H, Paulson JC (2004). Printed covalent glycan array for ligand profiling of diverse glycan binding proteins. Proc Natl Acad Sci U S A.

[11] Porter A, Yue T, Heeringa L, Day S, Suh E, Haab BB (2010). A motif-based analysis of glycan array data to determine the specificities of glycan-binding proteins. Glycobiology.

[12] Kletter D, Singh S, Bern M, Haab BB (2013). Global comparisons of lectin-glycan interactions using a database of analyzed glycan array data. Mol Cell Proteome.

[13] Hashimoto K, Takigawa I, Shiga M, Kanehisa M, Mamitsuka H (2008). Mining significant tree patterns in carbohydrate sugar chains. Bioinformatics.

[14] Cholleti SR, Agravat S, Morris T, Saltz JH, Song X, Cummings RD, Smith DF (2012). Automated motif discovery from glycan array data. OMICS.

[15] Ichimiya T, Nishihara S, Takase-Yoden S, Kida H, Aoki-Kinoshita K (2014). Frequent glycan structure mining of influenza virus data revealed a sulfated glycan motif that increased viral infection. Bioinformatics.

[16] Hosoda M, Akune Y, Aoki-Kinoshita KF (2017). Development and application of an algorithm to compute weighted multiple glycan alignments. Bioinformatics.

[17] Hosoda M, Takahashi Y, Shiota M, Shinmachi D, Inomoto R, Higashimoto S, Aoki-Kinoshita KF (2018). MCAW-DB: A glycan profile database capturing the ambiguity of glycan recognition patterns. Carbohydr Res.

[18] Klamer Z, Staal B, Prudden AR, Liu L, Smith DF, Boons G-J, Haab B (2017). Mining high-complexity motifs in glycans: A new language to uncover the fine specificities of lectins and glycosidases. Anal Chem.

[19] Iglewicz B, Hoaglin DC (1993). How to Detect and Handle Outliers.

[20] Yan X, Han J. gspan: graph-based substructure pattern mining. In: 2002 IEEE International Conference on Data Mining, 2002. Proceedings: 2002. p. 721–4. 10.1109/icdm.2002.1184038.

[21] Zhou K. 2019. https://github.com/Jokeren/gBolt. Accessed 14 Jun 2019.

[22] Peng H, Long F, Ding C (2005). Feature selection based on mutual information: criteria of max-dependency, max-relevance, and min-redundancy. IEEE Trans Pattern Anal Mach Intell.

[23] Ramírez-Gallego S, Lastra I, Martínez-Rego D, Bolón-Canedo V, Benítez JM, Herrera F, Alonso-Betanzos A (2017). Fast-mRMR: Fast minimum redundancy maximum relevance algorithm for high-dimensional big data. Int J Intell Syst.

[24] Seabold S, Perktold J. Statsmodels: Econometric and statistical modeling with python. In: Proc. of the 9th Python in Science Conf: 2010. http://www.statsmodels.org/stable/index.html.

[25] Wang L, Cummings RD, Smith DF, Huflejt M, Campbell CT, Gildersleeve JC, Gerlach JQ, Kilcoyne M, Joshi L, Serna S, Reichardt N-C, Parera Pera N, Pieters RJ, Eng W, Mahal LK (2014). Cross-platform comparison of glycan microarray formats. Glycobiology.

[26] Logtenberg T, de Gast GC, Nieuwenhuis P, van den Broek AA, Hanna MG (1982). Peanut agglutinin (PNA) binding as a marker for immature human B lymphocytes. is bone marrow not the complete bursa-equivalent?. In Vivo Immunology: Histophysiology of the Lymphoid System.

[27] Chacko BK, Appukuttan PS (2001). Peanut (*Arachis hypogaea*) lectin recognizes alpha-linked galactose, but not N-acetyl lactosamine in N-linked oligosaccharide terminals. Int J Biol Macromol.

[28] Ravishankar R, Ravindran M, Suguna K, Surolia A, Vijayan M (1997). The specificity of peanut agglutinin for Thomsen-Friedenreich antigen is mediated by water-bridges. Curr Sci.

[29] Coligan JE, Dunn BM, Speicher DW, Wingfield PT (2001). Lectin affinity chromatography. Current Protocols in Protein Science, vol. 230.

[30] Brewer CF, Bhattacharyya L (1986). Specificity of concanavalin A binding to asparagine-linked glycopeptides. a nuclear magnetic relaxation dispersion study. J Biol Chem.

[31] Moothoo DN, Naismith JH (1998). Concanavalin A distorts the *β*-GlcNAc-(1 →2)-Man linkage of *β*-GlcNAc-(1 →2)- *α*-Man-(1 →3)-[ *β*-Gl cNAc-(1 →2)- *α*-Man-(1 →6)]-Man upon binding. Glycobiology.

[32] Alroy J, Goyal V, Skutelsky E (1987). Lectin histochemistry of mammalian endothelium. Histochemistry.

[33] Itakura Y, Nakamura-Tsuruta S, Kominami J, Sharon N, Kasai K-I, Hirabayashi J (2007). Systematic comparison of oligosaccharide specificity of *Ricinus communis* agglutinin I and *Erythrina* lectins: a search by frontal affinity chromatography. J Biochem.

[34] Song X, Yu H, Chen X, Lasanajak Y, Tappert MM, Air GM, Tiwari VK, Cao H, Chokhawala HA, Zheng H, Cummings RD, Smith DF (2011). A sialylated glycan microarray reveals novel interactions of modified sialic acids with proteins and viruses. J Biol Chem.

[35] Worbs S, Skiba M, Söderström M, Rapinoja M-L, Zeleny R, Russmann H, Schimmel H, Vanninen P, Fredriksson S-Å, Dorner BG (2015). Characterization of ricin and *R. communis* agglutinin reference materials. Toxins.

[36] Couceiro JN, Paulson JC, Baum LG (1993). Influenza virus strains selectively recognize sialyloligosaccharides on human respiratory epithelium; the role of the host cell in selection of hemagglutinin receptor specificity. Virus Res.

[37] Geisler C, Jarvis DL (2011). Effective glycoanalysis with *Maackia amurensis* lectins requires a clear understanding of their binding specificities. Glycobiology.

[38] Yamashita K, Hitoi A, Kobata A (1983). Structural determinants of *Phaseolus vulgaris* erythroagglutinating lectin for oligosaccharides. J Biol Chem.

[39] Monzingo AF, Robertus JD (1992). X-ray analysis of substrate analogs in the ricin A-chain active site. J Mol Biol.

[40] Palma AS, Feizi T, Childs RA, Chai W, Liu Y (2014). The neoglycolipid (NGL)-based oligosaccharide microarray system poised to decipher the meta-glycome. Curr Opin Chem Biol.

[41] Grant OC, Smith HMK, Firsova D, Fadda E, Woods RJ (2014). Presentation, presentation, presentation! molecular-level insight into linker effects on glycan array screening data. Glycobiology.

[42] Nakamura-Tsuruta S, Kominami J, Kuno A, Hirabayashi J (2006). Evidence that *Agaricus bisporus* agglutinin (ABA) has dual sugar-binding specificity. Biochem Biophys Res Commun.

[43] Bories PN, Feger J, Benbernou N, Rouzeau JD, Agneray J, Durand G (1990). Prevalence of tri- and tetraantennary glycans of human alpha 1-acid glycoprotein in release of macrophage inhibitor of interleukin-1 activity. Inflammation.

[44] Cummings RD (2009). The repertoire of glycan determinants in the human glycome. Mol Biosyst.

[45] Geissner A, Reinhardt A, Rademacher C, Johannssen T, Monteiro J, Lepenies B, Thépaut M, Fieschi F, Mrázková J, Wimmerova M, Schuhmacher F, Götze S, Grünstein D, Guo X, Hahm HS, Kandasamy J, Leonori D, Martin CE, Parameswarappa SG, Pasari S, Schlegel MK, Tanaka H, Xiao G, Yang Y, Pereira CL, Anish C, Seeberger PH (2019). Microbe-focused glycan array screening platform. Proc Natl Acad Sci U S A.

[46] Jankowska E, Parsons LM, Song X, Smith DF, Cummings RD, Cipollo JF (2018). A comprehensive *Caenorhabditis elegans* N-glycan shotgun array. Glycobiology.

[47] Kolarich Daniel, Rapp Erdmann, Struwe Weston B., Haslam Stuart M., Zaia Joseph, McBride Ryan, Agravat Sanjay, Campbell Matthew P., Kato Masaki, Ranzinger Rene, Kettner Carsten, York William S. (2013). The Minimum Information Required for a Glycomics Experiment (MIRAGE) Project: Improving the Standards for Reporting Mass-spectrometry-based Glycoanalytic Data. Molecular & Cellular Proteomics.

[48] Maupin KA, Liden D, Haab BB (2012). The fine specificity of mannose-binding and galactose-binding lectins revealed using outlier motif analysis of glycan array data. Glycobiology.

[49] Yu Y, Lasanajak Y, Song X, Hu L, Ramani S, Mickum ML, Ashline DJ, Prasad BVV, Estes MK, Reinhold VN, Cummings RD, Smith DF (2014). Human milk contains novel glycans that are potential decoy receptors for neonatal rotaviruses. Mol Cell Proteome.

[50] Hu L, Sankaran B, Laucirica DR, Patil K, Salmen W, Ferreon ACM, Tsoi PS, Lasanajak Y, Smith DF, Ramani S, Atmar RL, Estes MK, Ferreon JC, Prasad BVV (2018). Glycan recognition in globally dominant human rotaviruses. Nat Commun.

[51] Varki A, Cummings RD, Aebi M, Packer NH, Seeberger PH, Esko JD, Stanley P, Hart G, Darvill A, Kinoshita T, Prestegard JJ, Schnaar RL, Freeze HH, Marth JD, Bertozzi CR, Etzler ME, Frank M, Vliegenthart JF, Lütteke T, Perez S, Bolton E, Rudd P, Paulson J, Kanehisa M, Toukach P, Aoki-Kinoshita KF, Dell A, Narimatsu H, York W, Taniguchi N, Kornfeld S (2015). Symbol nomenclature for graphical representations of glycans. Glycobiology.

[52] Sanders DA, Moothoo DN, Raftery J, Howard AJ, Helliwell JR, Naismith JH (2001). The 1.2 Å resolution structure of the Con A-dimannose complex. J Mol Biol.

