# Supplementary material for: Identifying glycan motifs using a novel subtree mining approach
Source: BMC Bioinformatics. 2020 Feb 4;21:42. doi: 10.1186/s12859-020-3374-4 (PMC7001330; doi:10.1186/s12859-020-3374-4)

Glycan representation key

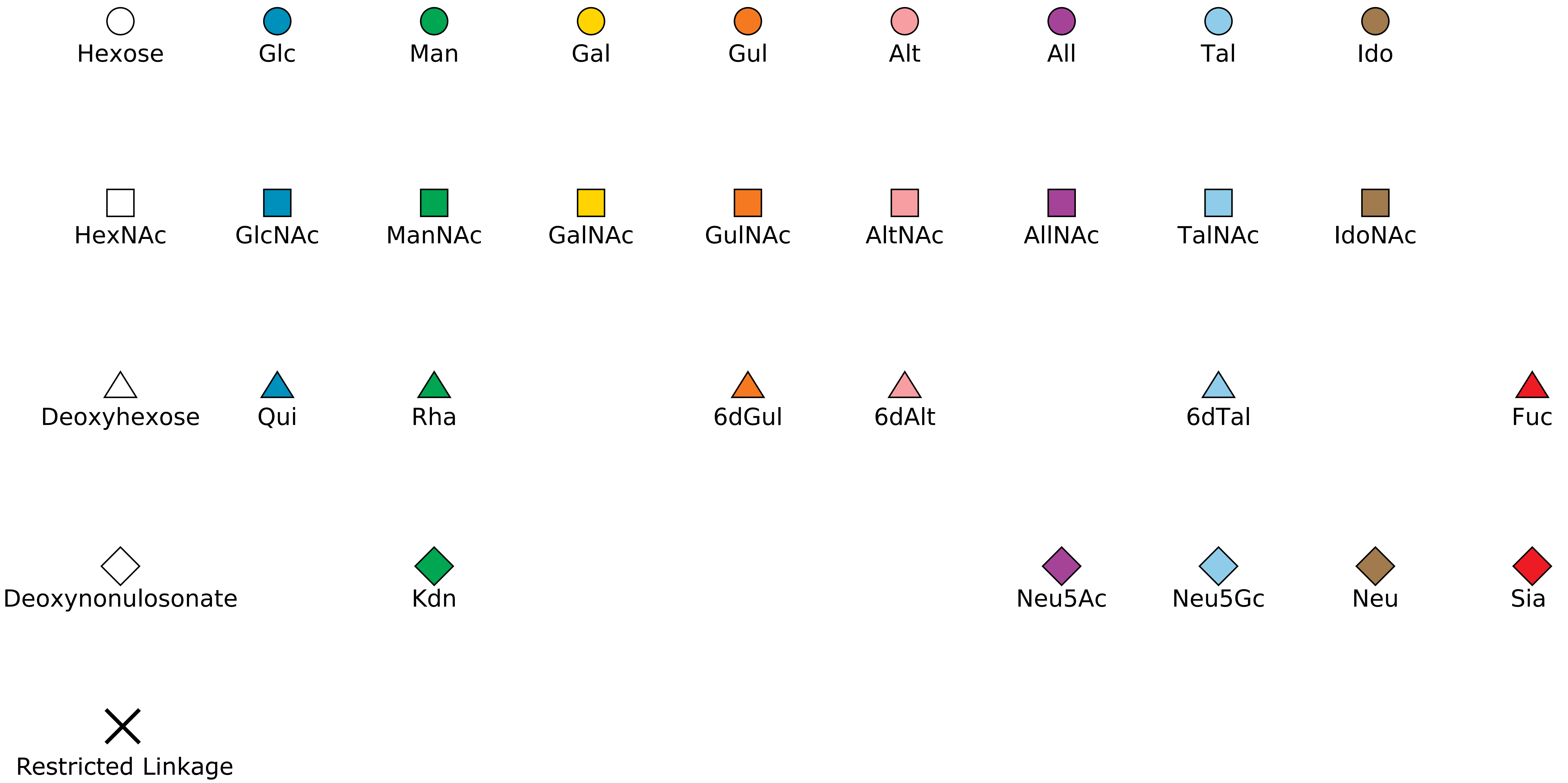

Supplement: Supplementary file 1 — Additional file 1 Modified SNFG key. The Symbol Nomenclature for Glycans (SNFG) [51] was used for drawing all glycans, with the addition of a cross to indicate a restricted linkage node. [file 12859_2020_3374_MOESM1_ESM.pdf]
