# Supplementary figures and images for: Identifying glycan motifs using a novel subtree mining approach
Source: BMC Bioinformatics. 2020 Feb 4;21:42. doi: 10.1186/s12859-020-3374-4 (PMC7001330; doi:10.1186/s12859-020-3374-4)

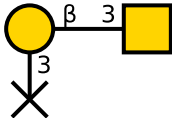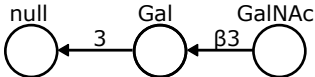

Supplement: Supplementary file 2 — Additional file 2 Representation of glycans as directed, labelled graphs. Glycans were represented as directed, labelled graphs, with nodes labelled with monosaccharide type, and edges labelled with connection type (i.e. anomer and linkage position). Additional nodes were added to represent the absence of a potential link, and termed restricted linkage nodes. [file 12859_2020_3374_MOESM2_ESM.pdf]

GLYMMR MOTIFS

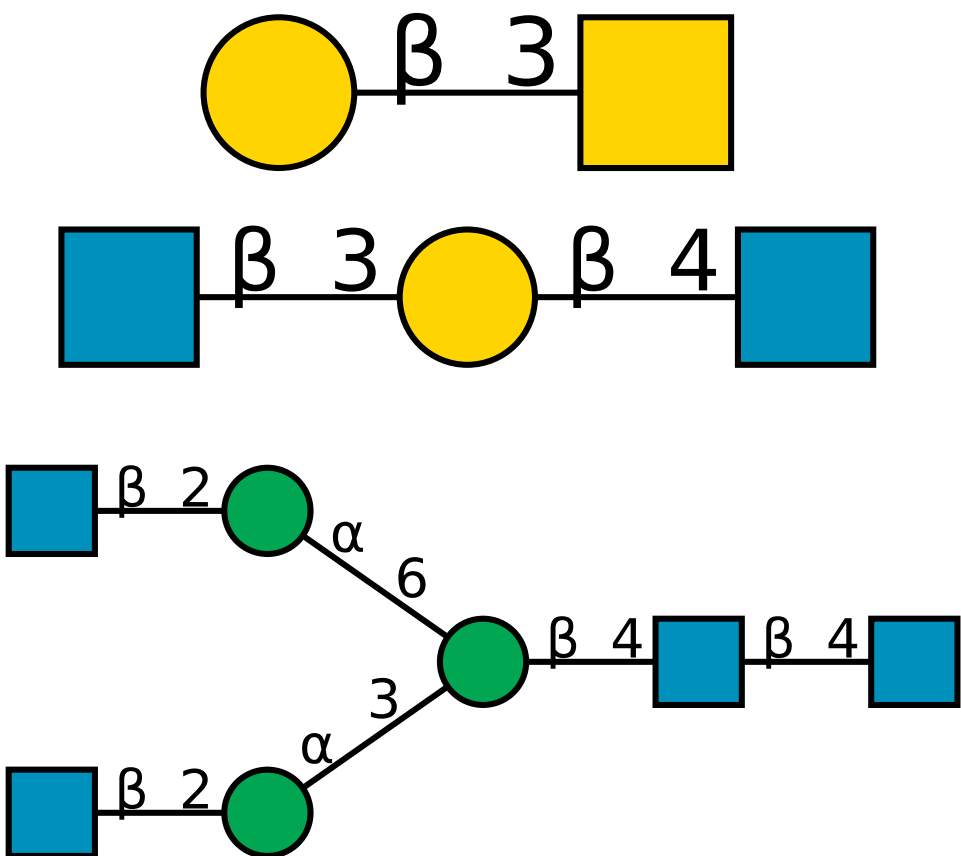

GLYCAN MOTIF MINER MOTIFS

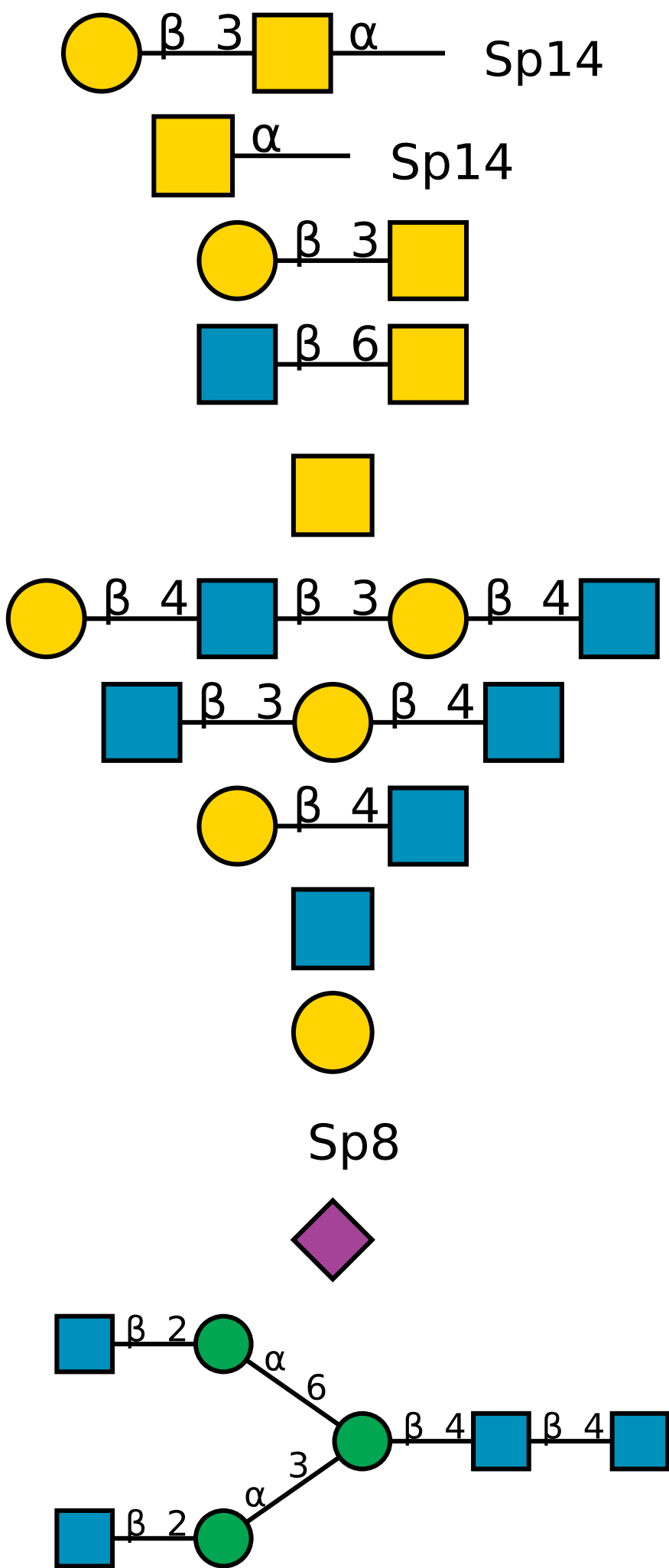

CCARL MOTIFS

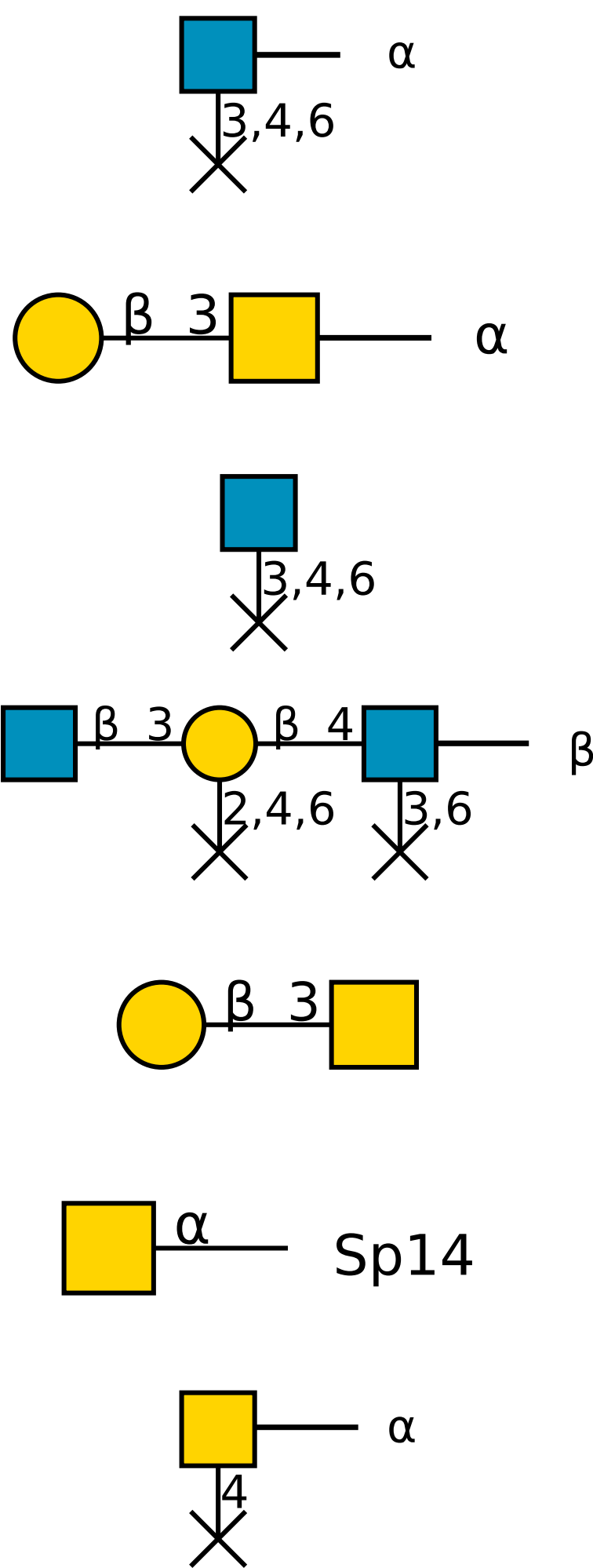

Supplement: Supplementary file 8 — Additional file 8 Motifs from GLYMMR and glycan motif miner. Motifs extracted using GLYMMR and Glycan Miner Tool for a range of glycan microarray datasets. [file 12859_2020_3374_MOESM8_ESM.zip › ABA.pdf]

## GLYMMR MOTIFS

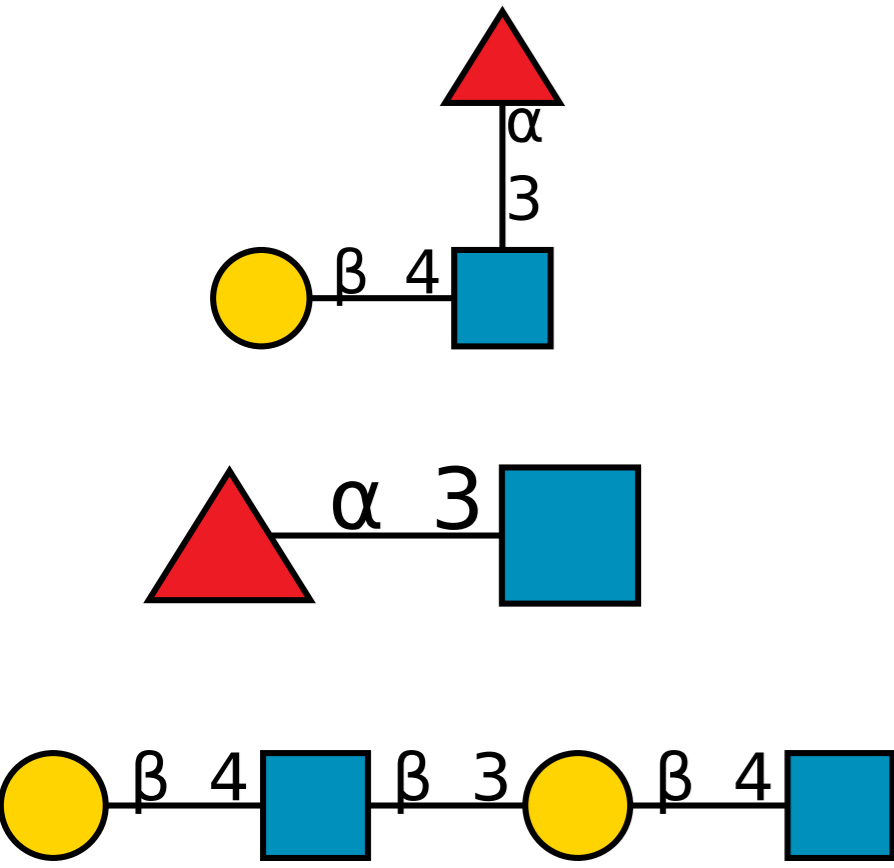

## GLYCAN MOTIF MINER MOTIFS

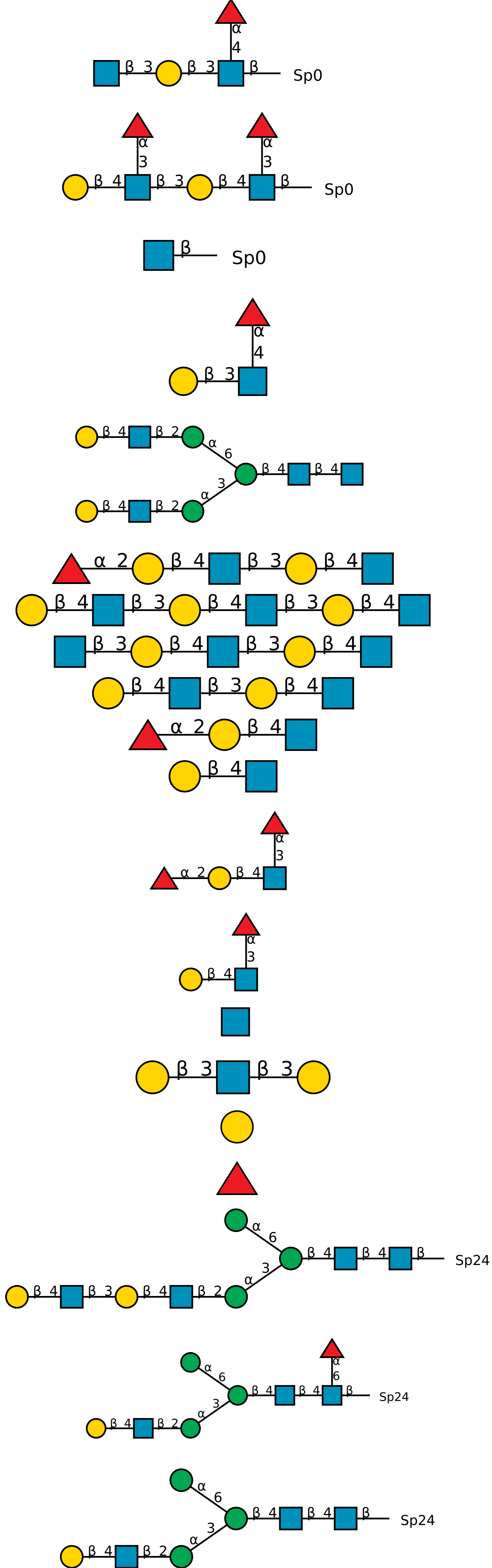

## CCARL MOTIFS

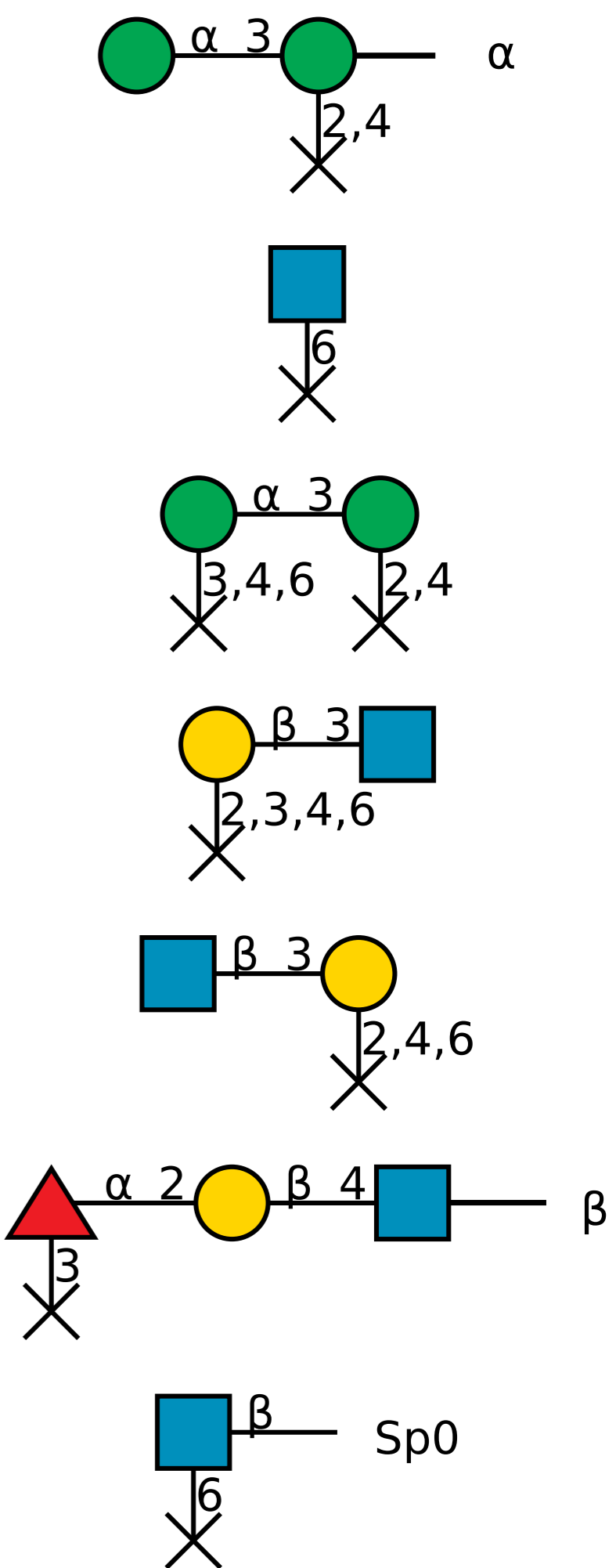

Supplement: Supplementary file 8 — Additional file 8 Motifs from GLYMMR and glycan motif miner. Motifs extracted using GLYMMR and Glycan Miner Tool for a range of glycan microarray datasets. [file 12859_2020_3374_MOESM8_ESM.zip › DC-Sign.pdf]

GLYMMR MOTIFS

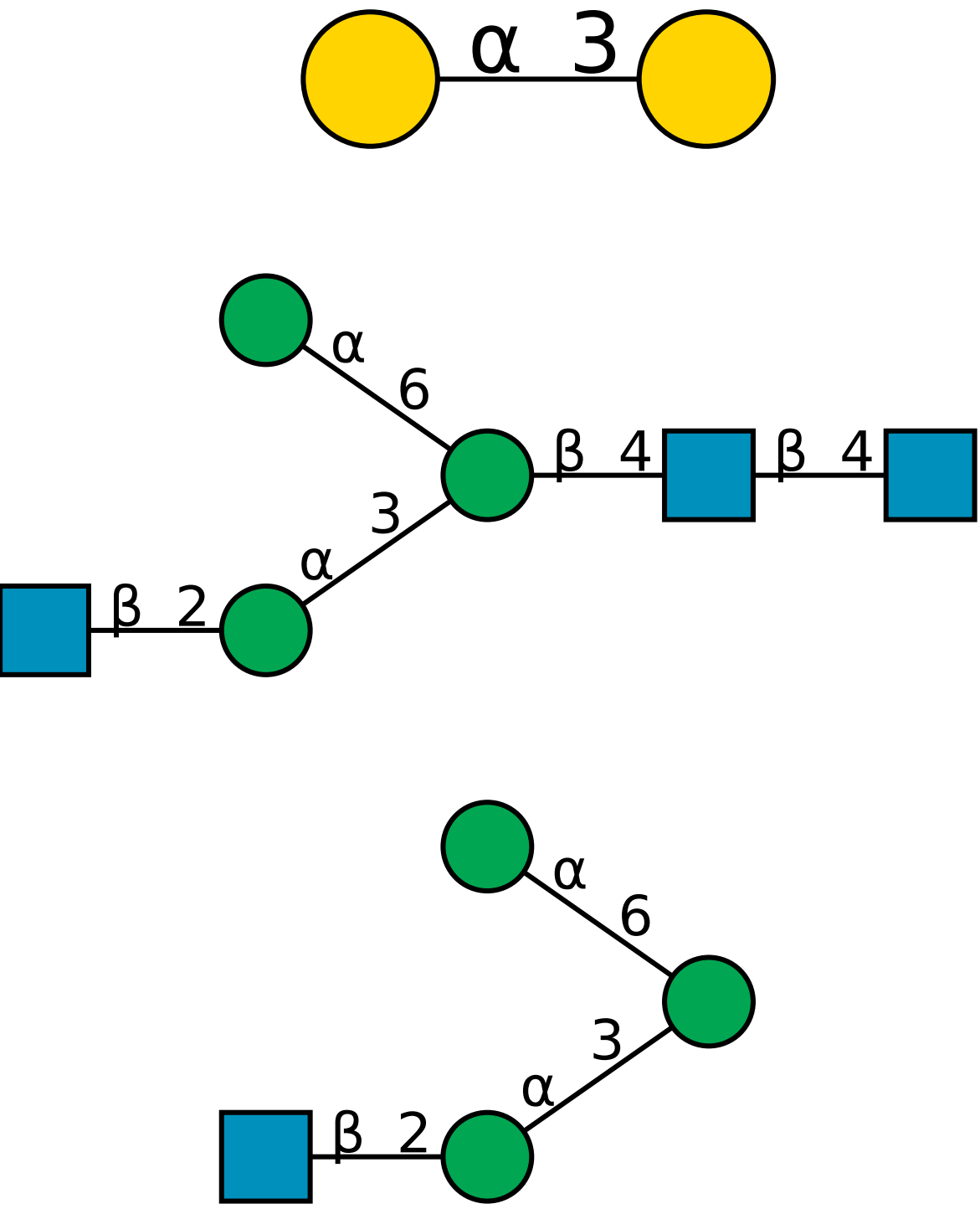

GLYCAN MOTIF MINER MOTIFS

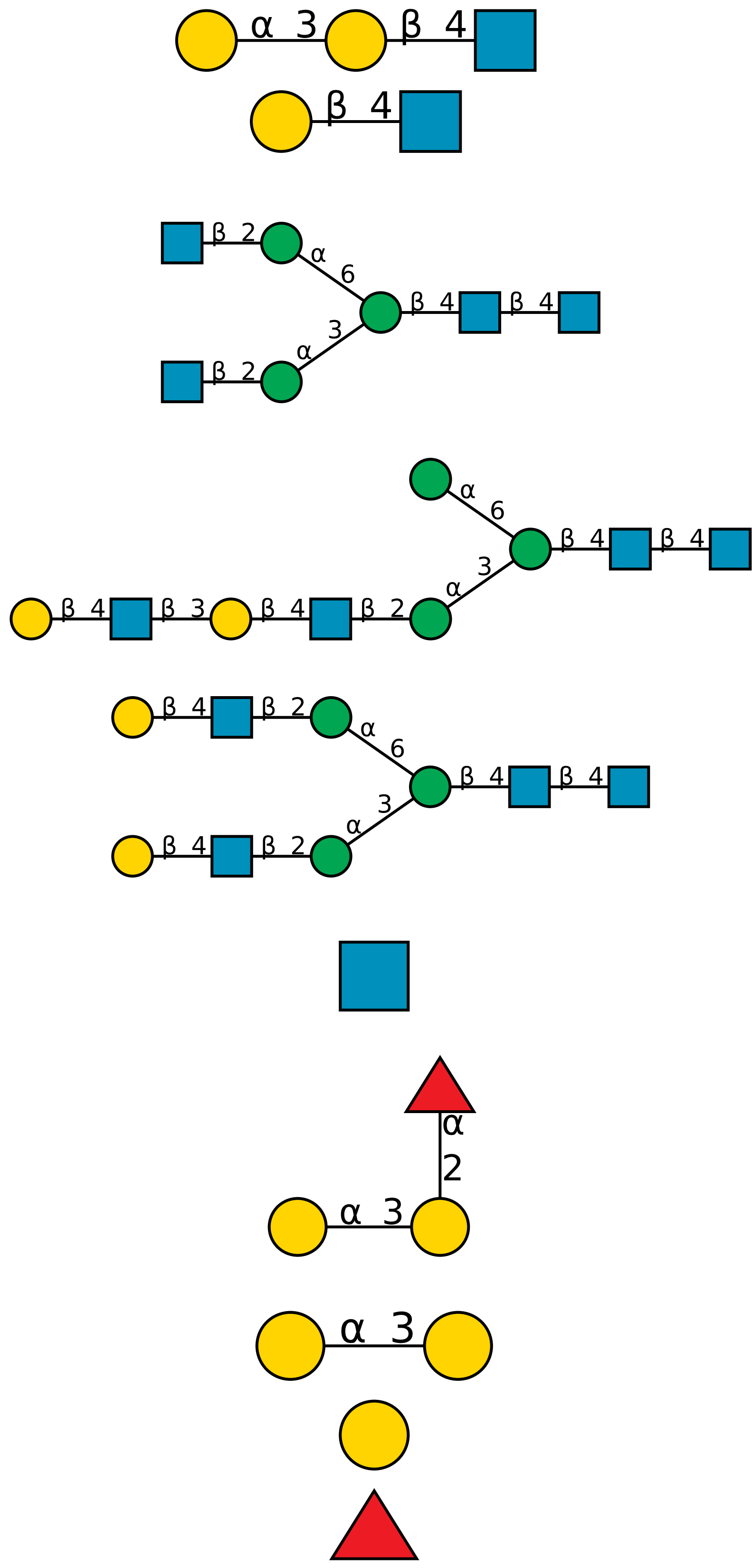

CCARL MOTIFS

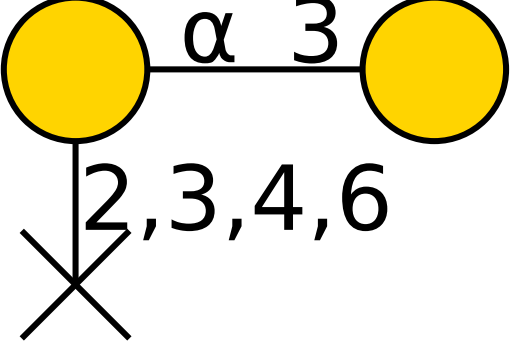

Sp0

Sp8

Supplement: Supplementary file 8 — Additional file 8 Motifs from GLYMMR and glycan motif miner. Motifs extracted using GLYMMR and Glycan Miner Tool for a range of glycan microarray datasets. [file 12859_2020_3374_MOESM8_ESM.zip › GSL-I.pdf]

GLYMMR MOTIFS

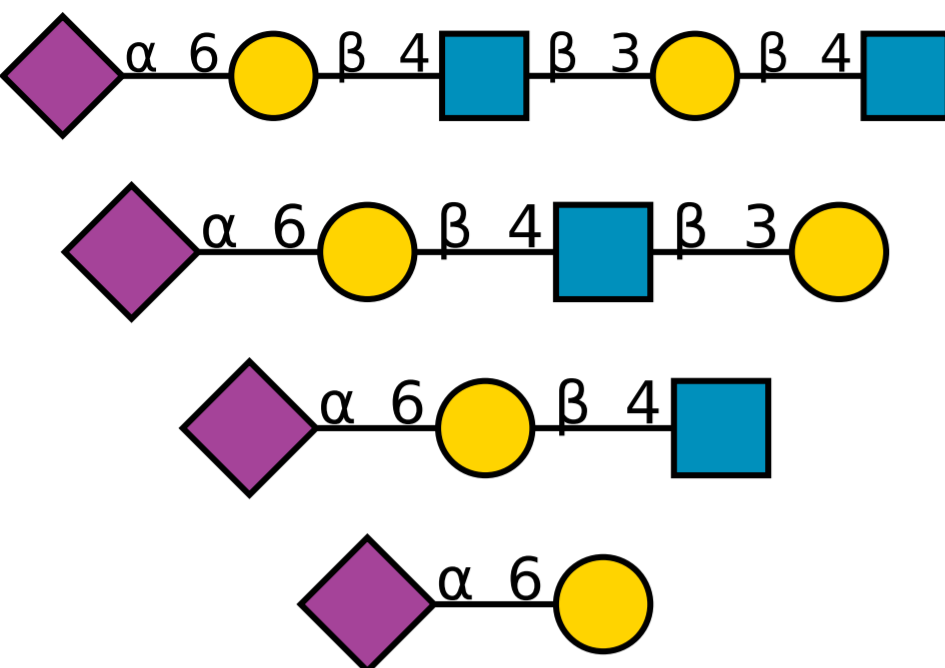

GLYCAN MOTIF MINER MOTIFS

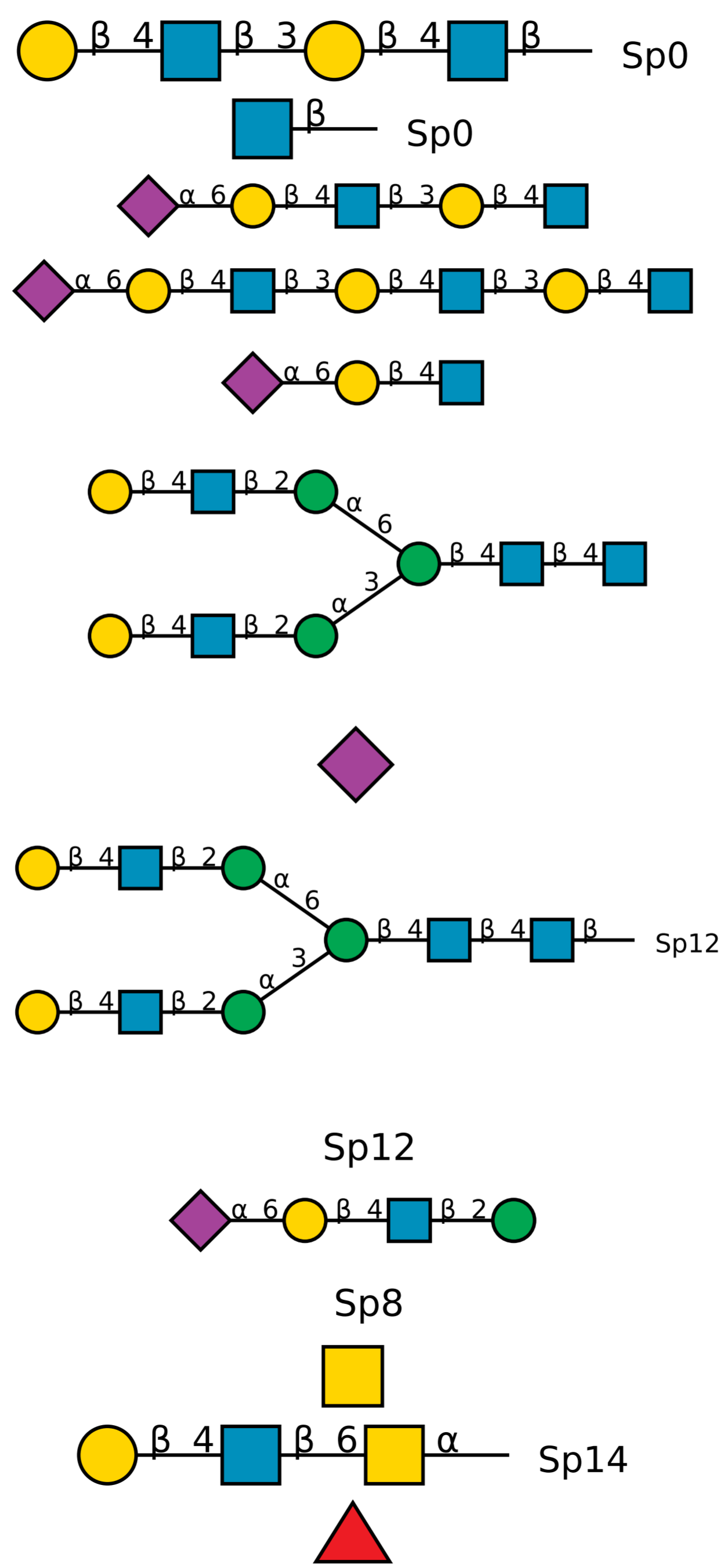

CCARL MOTIFS

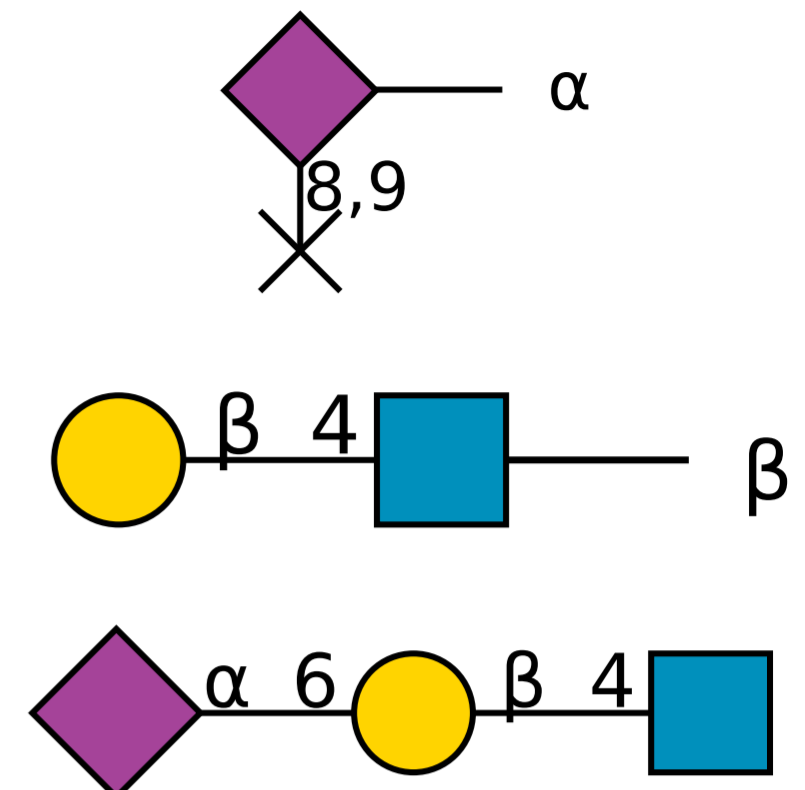

Supplement: Supplementary file 8 — Additional file 8 Motifs from GLYMMR and glycan motif miner. Motifs extracted using GLYMMR and Glycan Miner Tool for a range of glycan microarray datasets. [file 12859_2020_3374_MOESM8_ESM.zip › H1N1.pdf]

GLYMMR MOTIFS

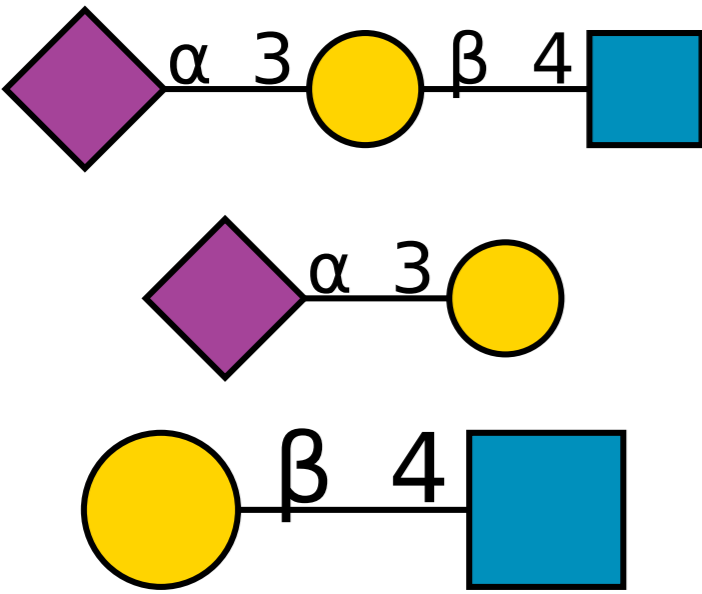

GLYCAN MOTIF MINER MOTIFS

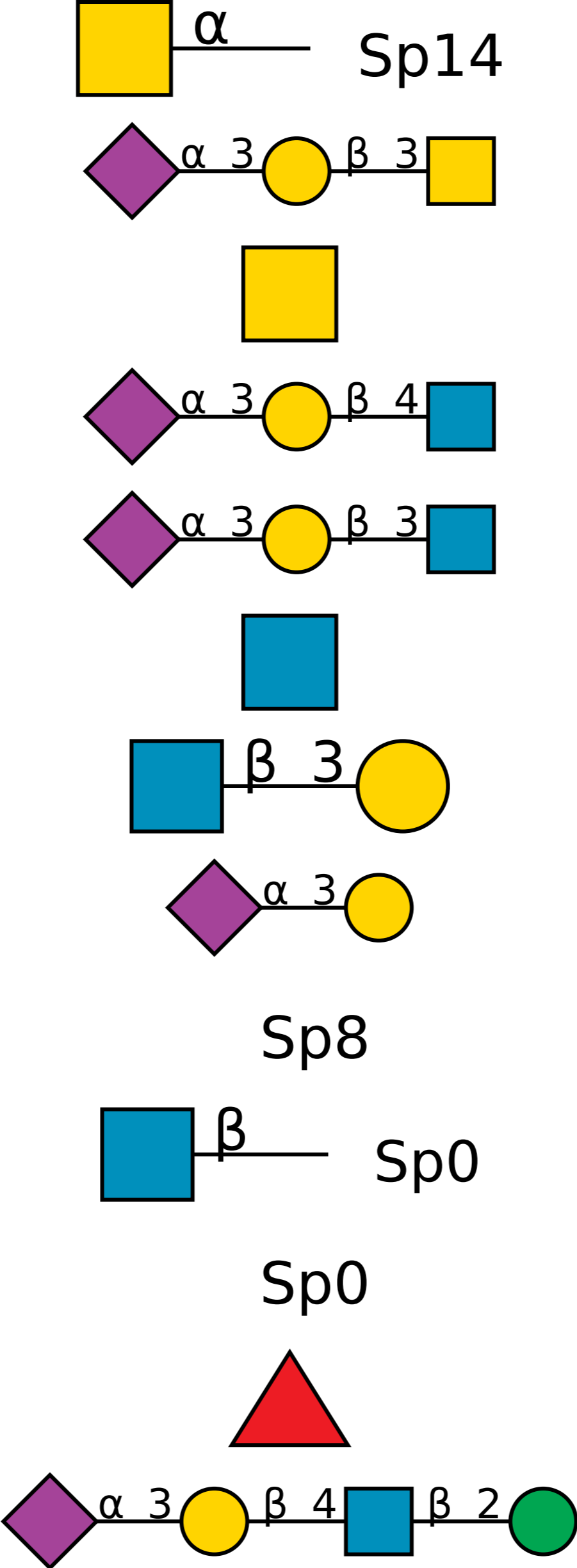

CCARL MOTIFS

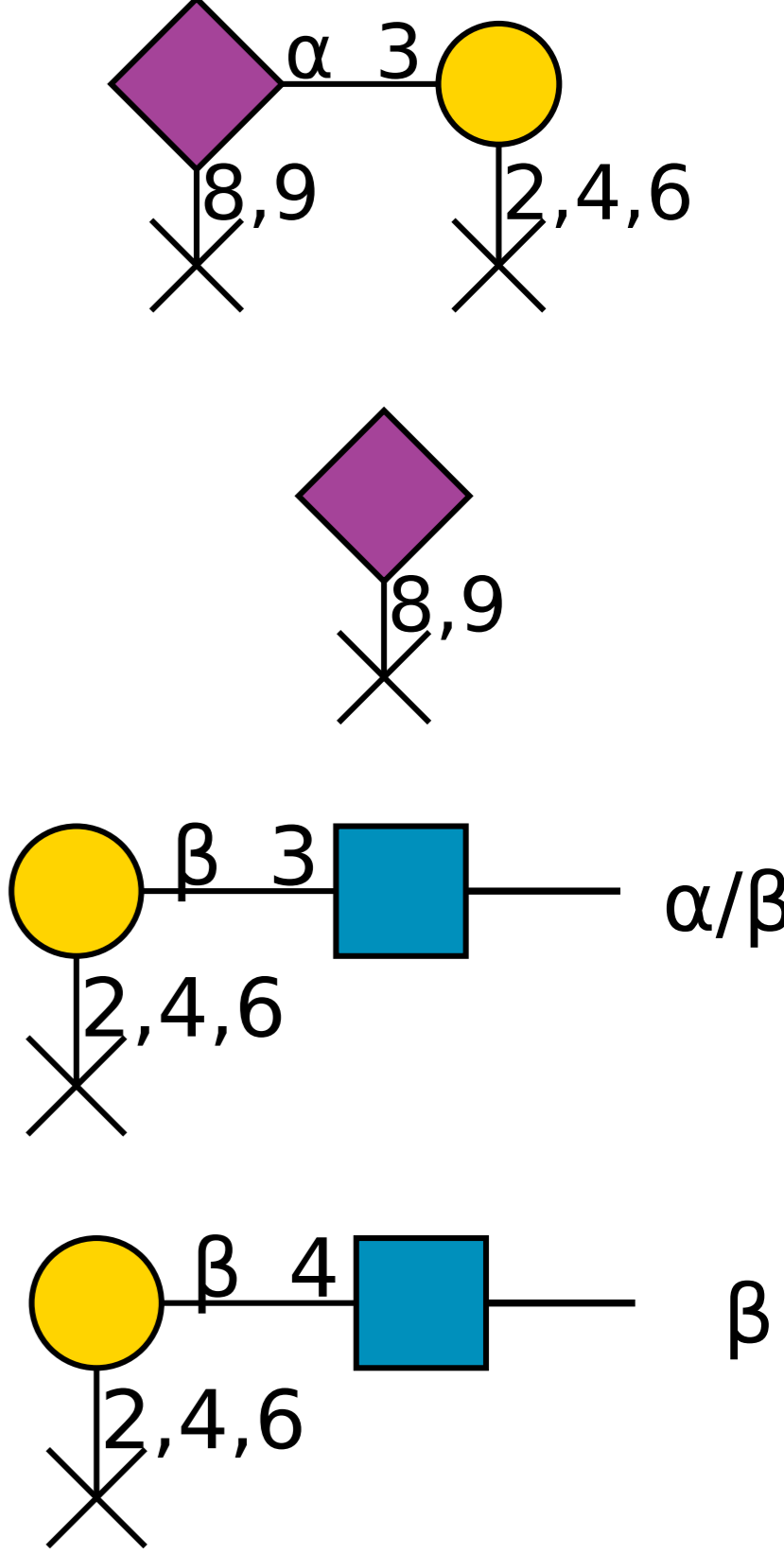

Supplement: Supplementary file 8 — Additional file 8 Motifs from GLYMMR and glycan motif miner. Motifs extracted using GLYMMR and Glycan Miner Tool for a range of glycan microarray datasets. [file 12859_2020_3374_MOESM8_ESM.zip › H3N8.pdf]

GLYMMR MOTIFS

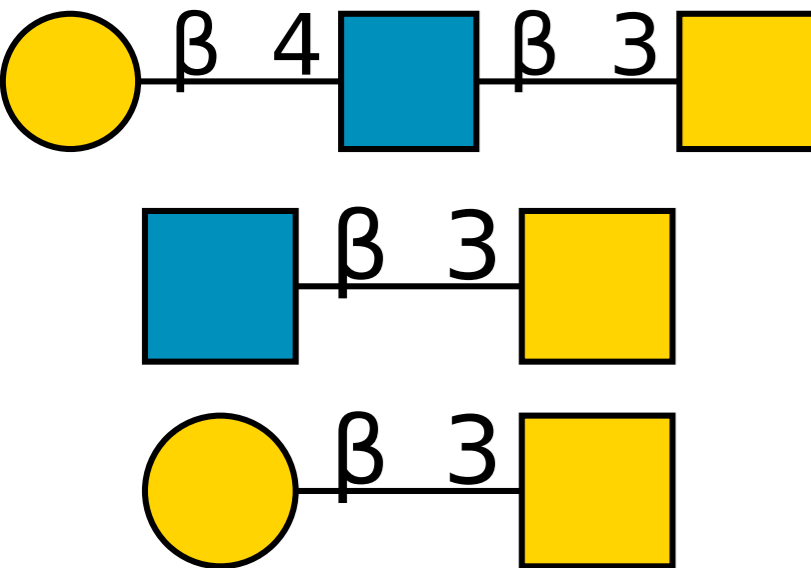

GLYCAN MOTIF MINER MOTIFS

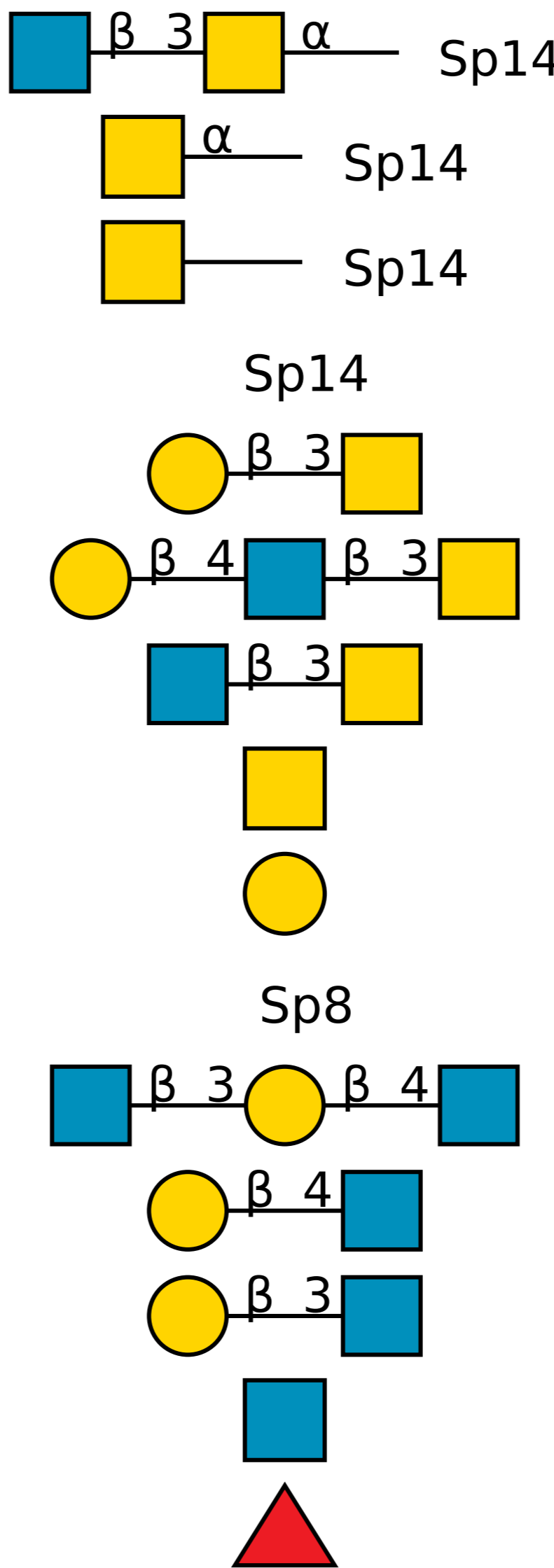

CCARL MOTIFS

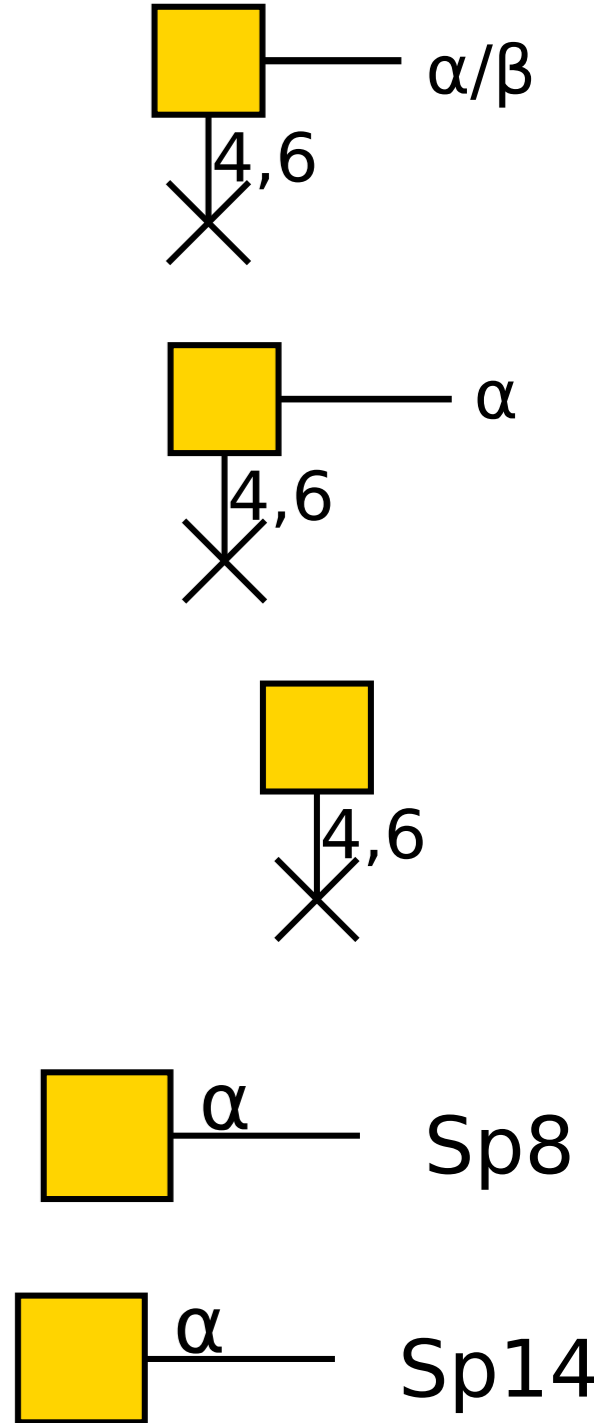

Supplement: Supplementary file 8 — Additional file 8 Motifs from GLYMMR and glycan motif miner. Motifs extracted using GLYMMR and Glycan Miner Tool for a range of glycan microarray datasets. [file 12859_2020_3374_MOESM8_ESM.zip › jacalin.pdf]

## GLYMMR MOTIFS

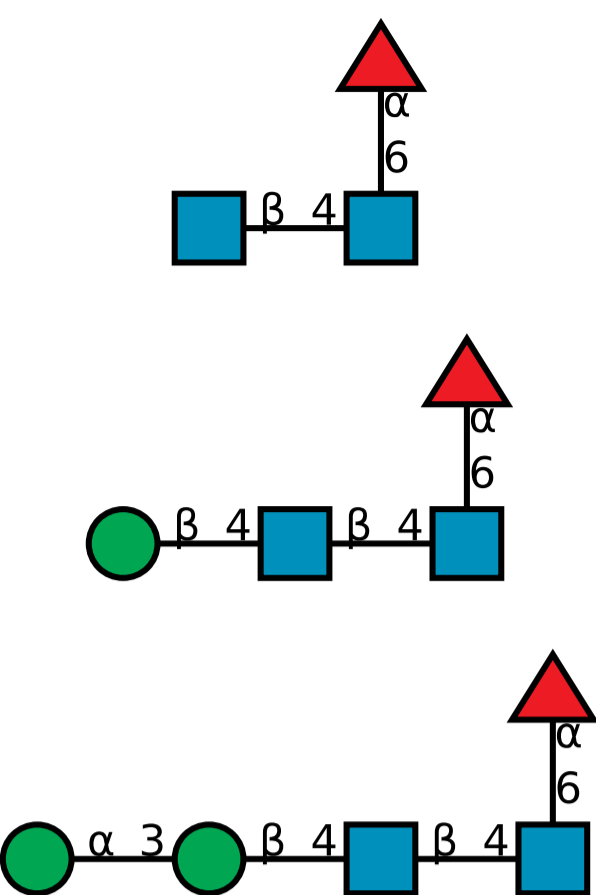

# GLYCAN MOTIF MINER MOTIFS

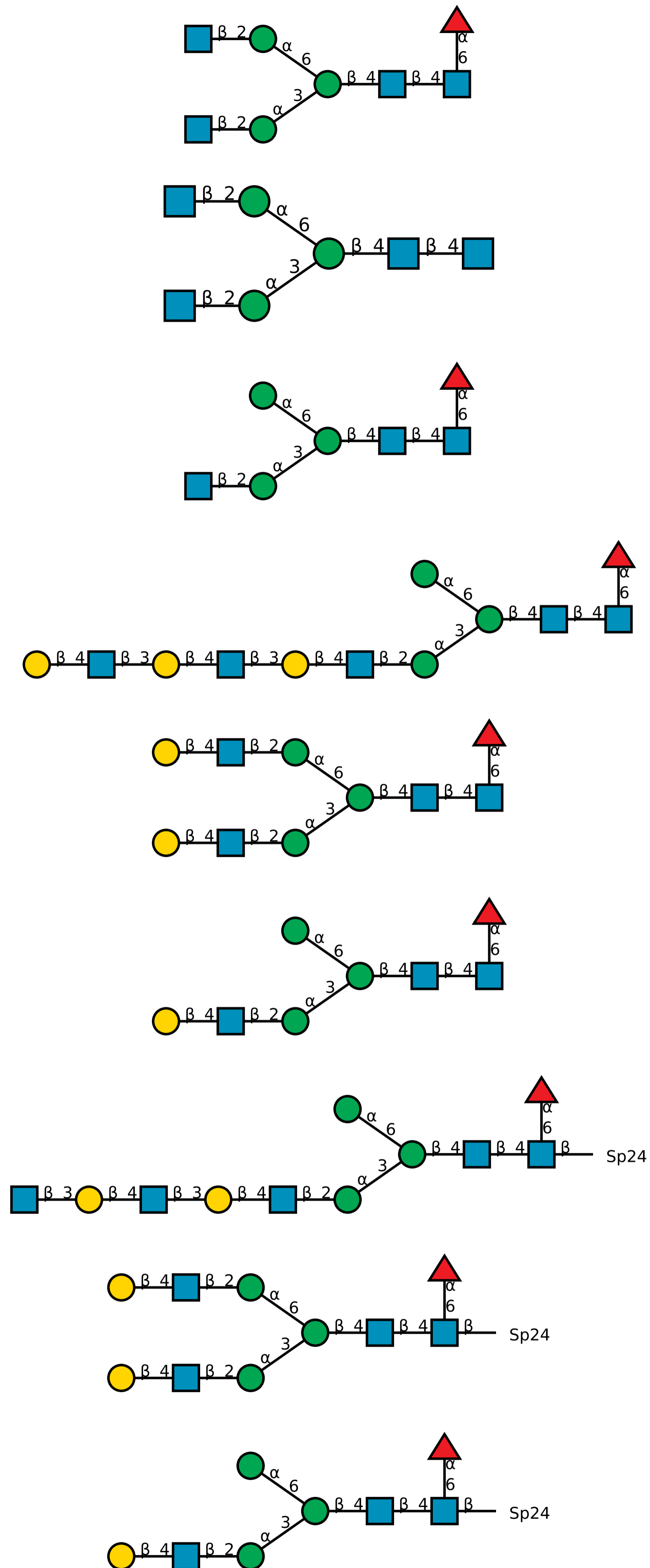

Sp12

## CCARL MOTIFS

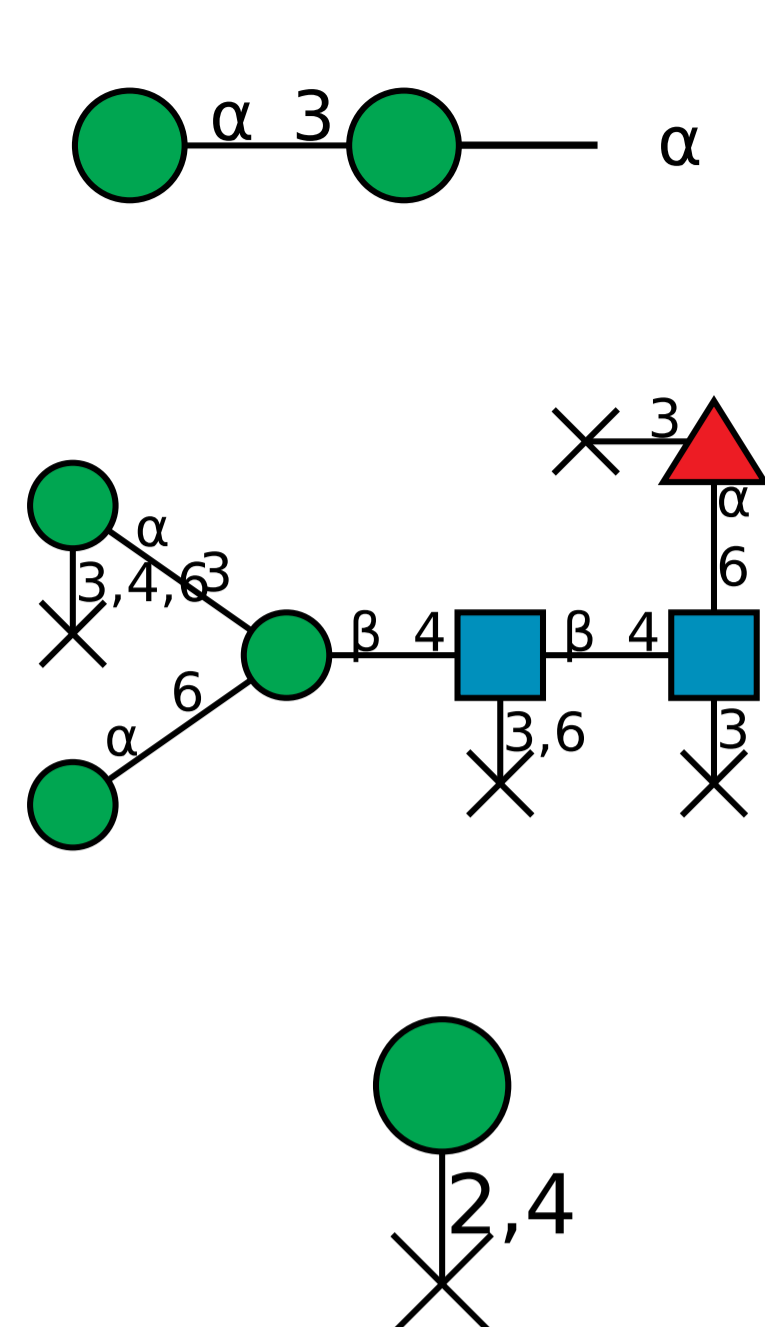

Supplement: Supplementary file 8 — Additional file 8 Motifs from GLYMMR and glycan motif miner. Motifs extracted using GLYMMR and Glycan Miner Tool for a range of glycan microarray datasets. [file 12859_2020_3374_MOESM8_ESM.zip › LCA.pdf]

GLYMMR MOTIFS

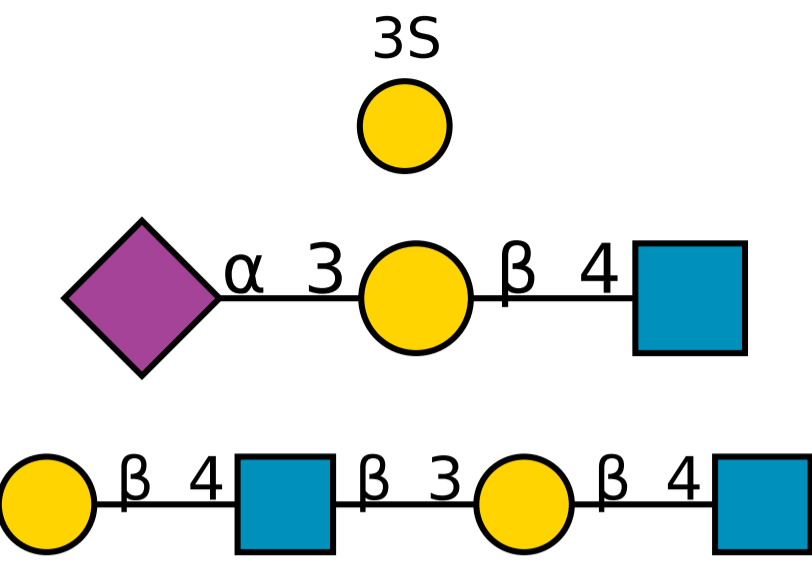

GLYCAN MOTIF MINER MOTIFS

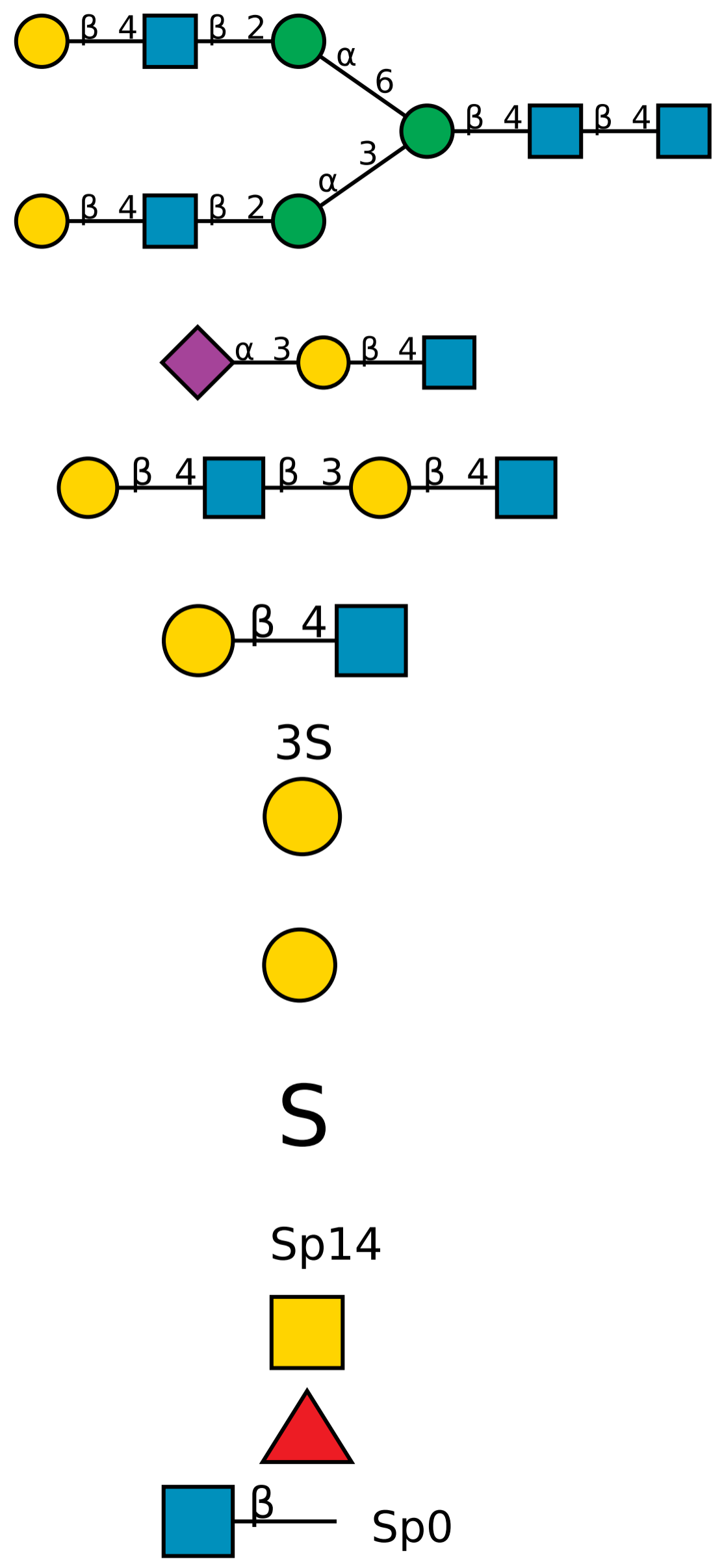

CCARL MOTIFS

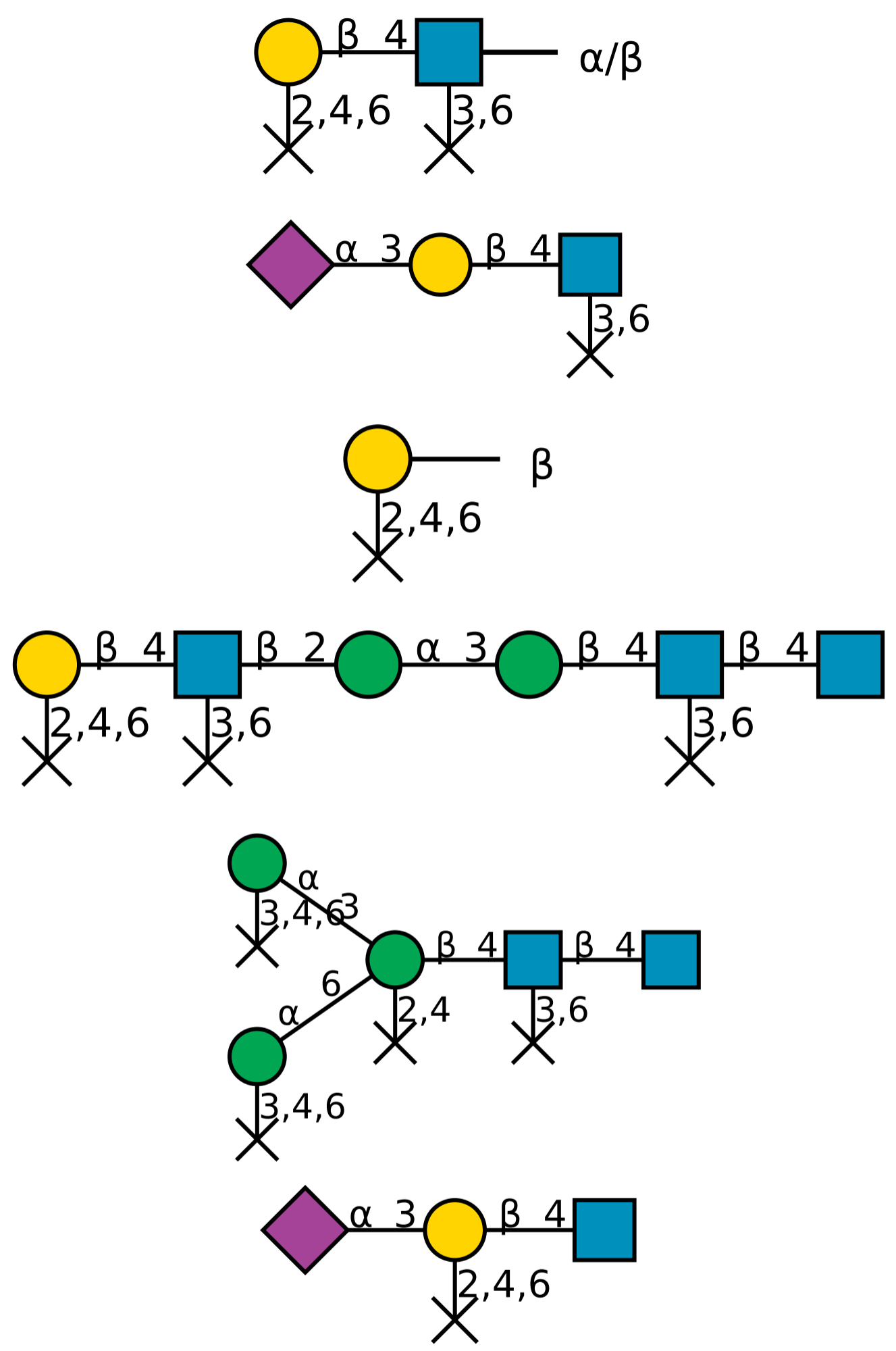

3S

S

Sp14

Sp0

Sp0

Supplement: Supplementary file 8 — Additional file 8 Motifs from GLYMMR and glycan motif miner. Motifs extracted using GLYMMR and Glycan Miner Tool for a range of glycan microarray datasets. [file 12859_2020_3374_MOESM8_ESM.zip › MAL-I.pdf]

GLYMMR MOTIFS

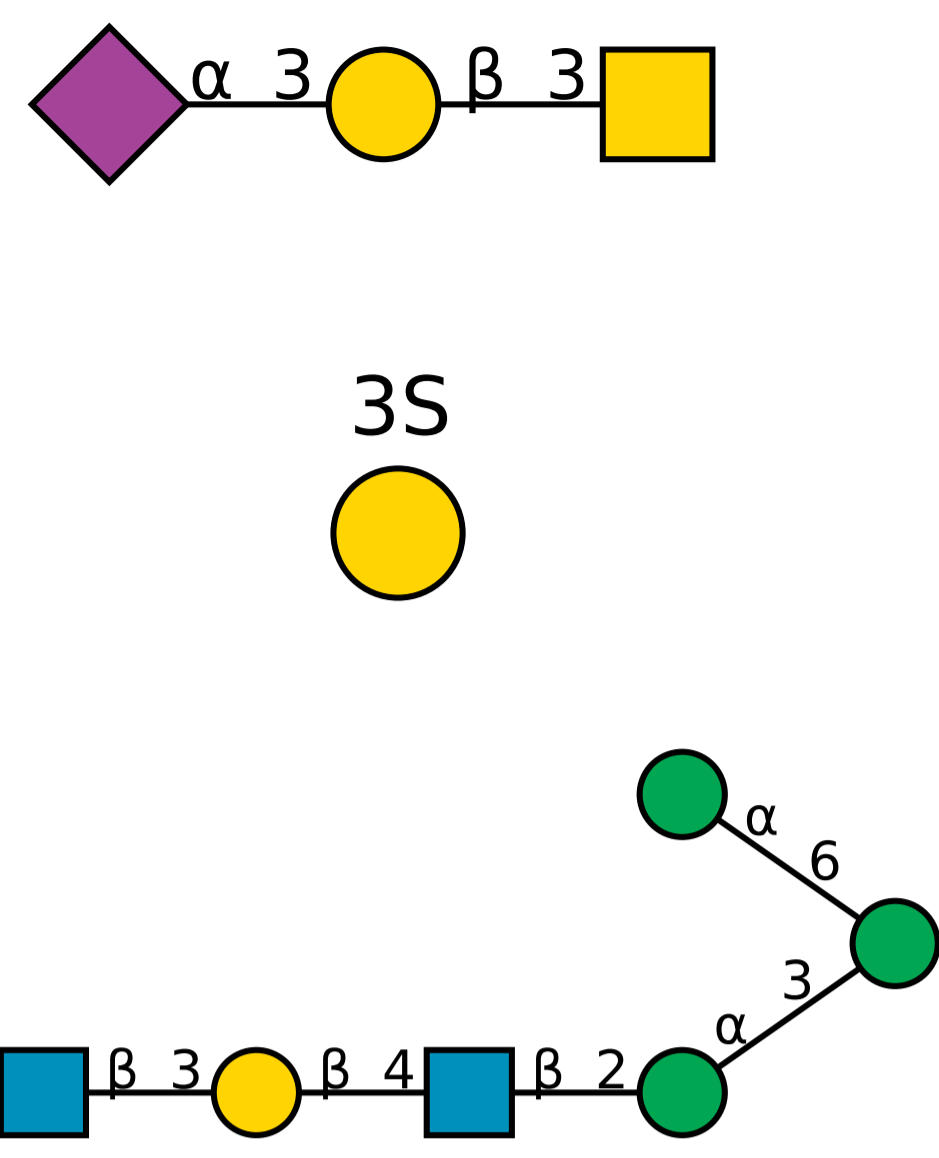

GLYCAN MOTIF MINER MOTIFS

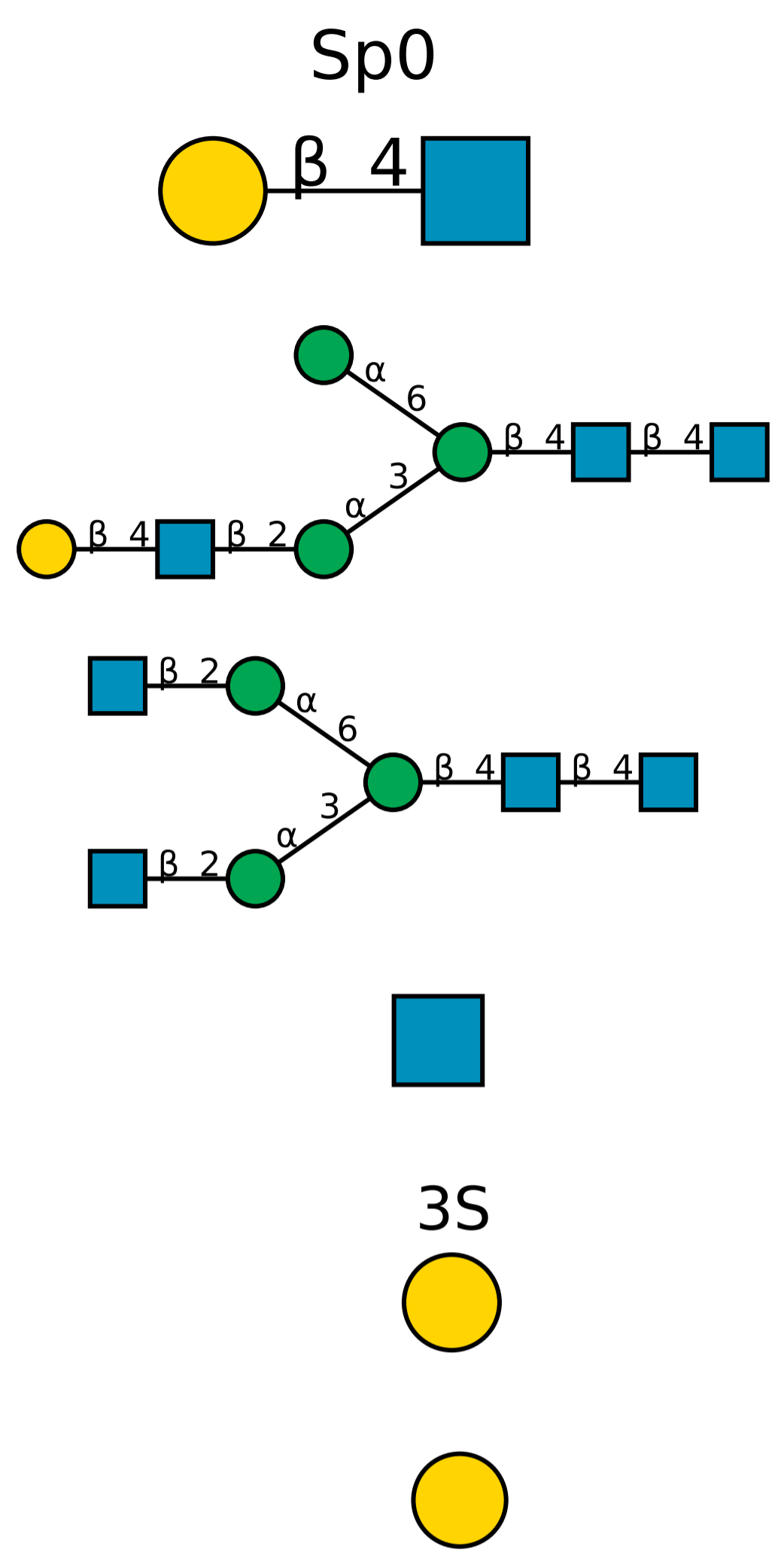

CCARL MOTIFS

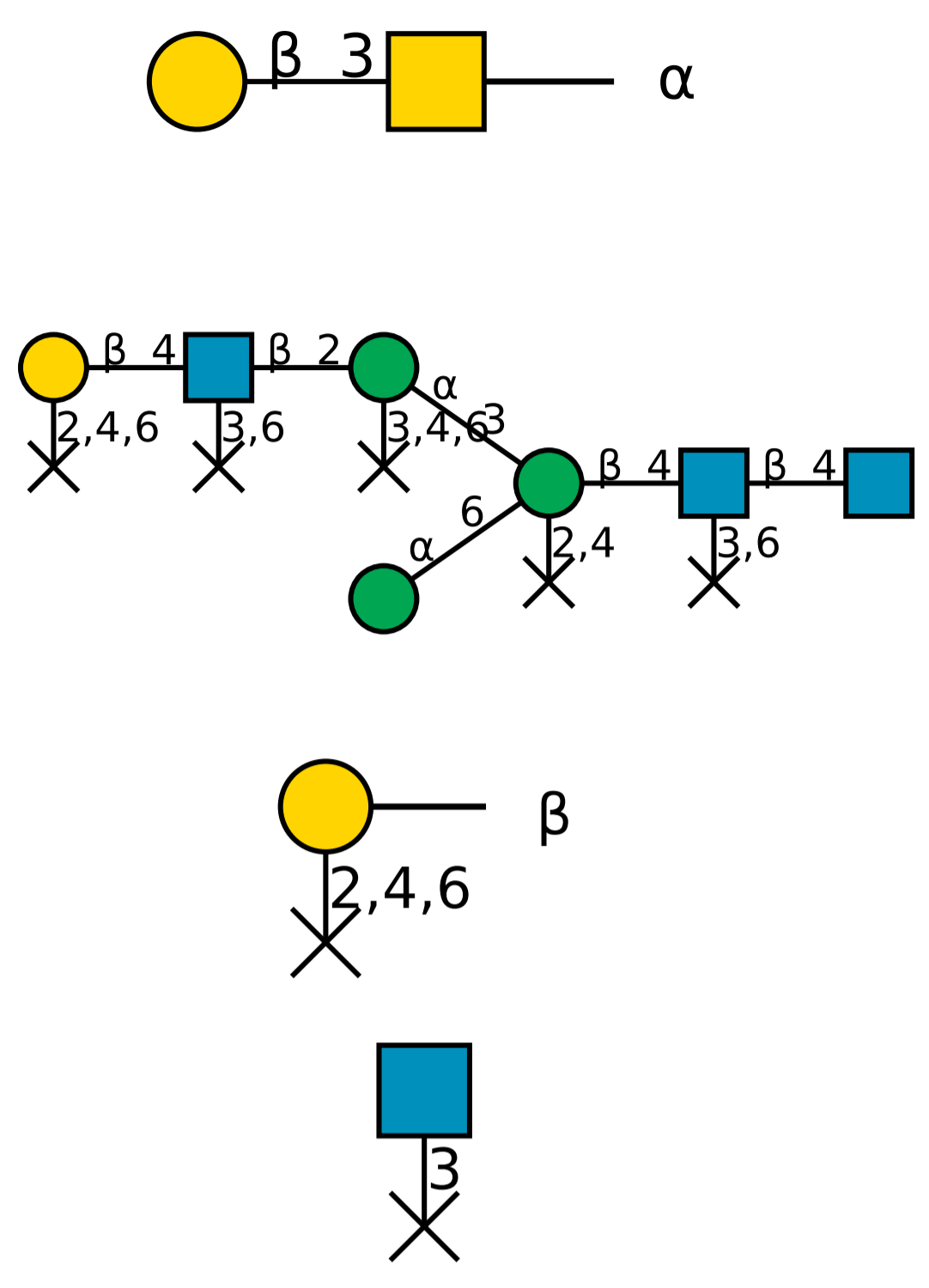

Sp8

Sp14

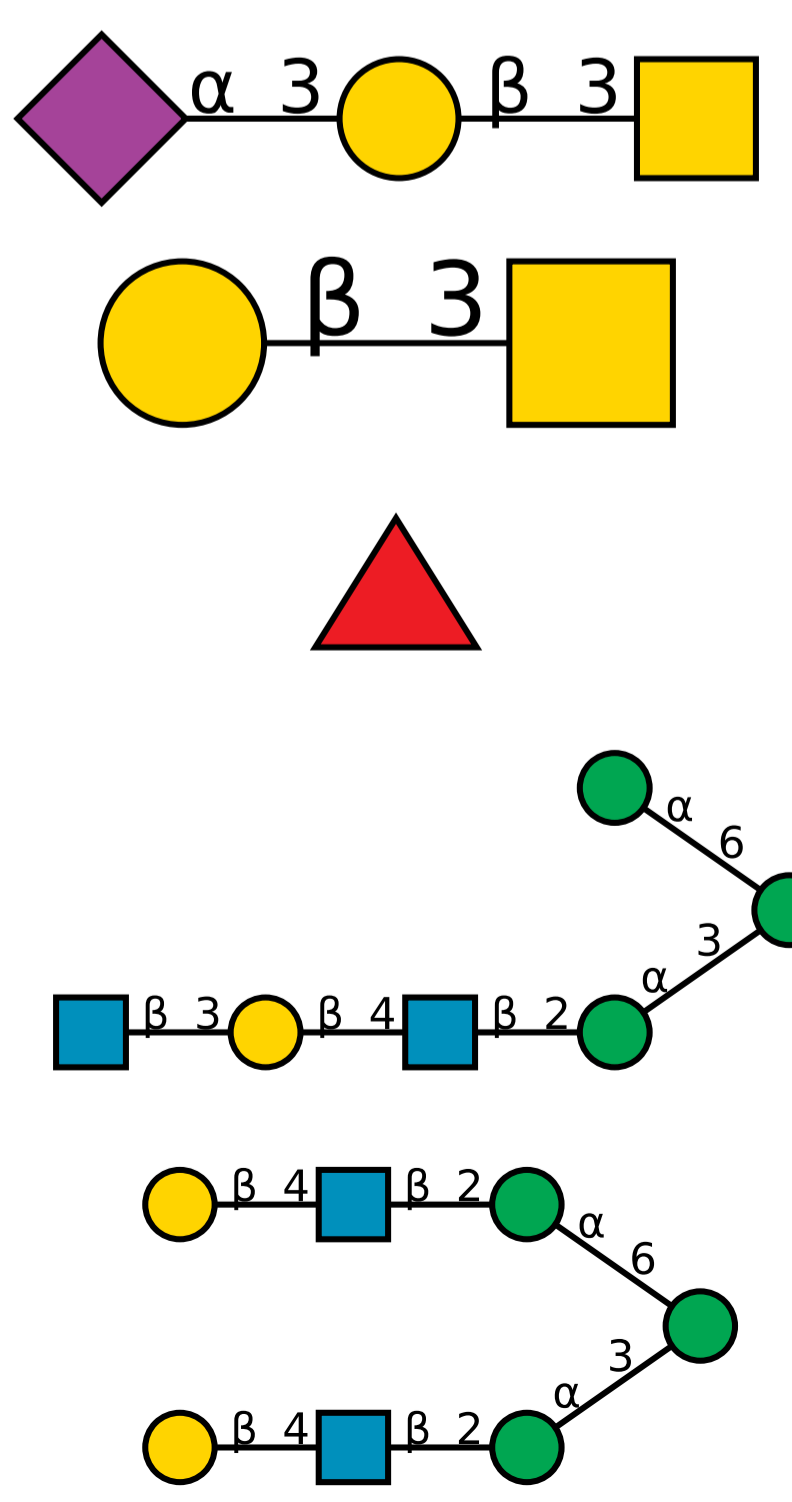

Supplement: Supplementary file 8 — Additional file 8 Motifs from GLYMMR and glycan motif miner. Motifs extracted using GLYMMR and Glycan Miner Tool for a range of glycan microarray datasets. [file 12859_2020_3374_MOESM8_ESM.zip › MAL-II.pdf]

# GLYMMR MOTIFS

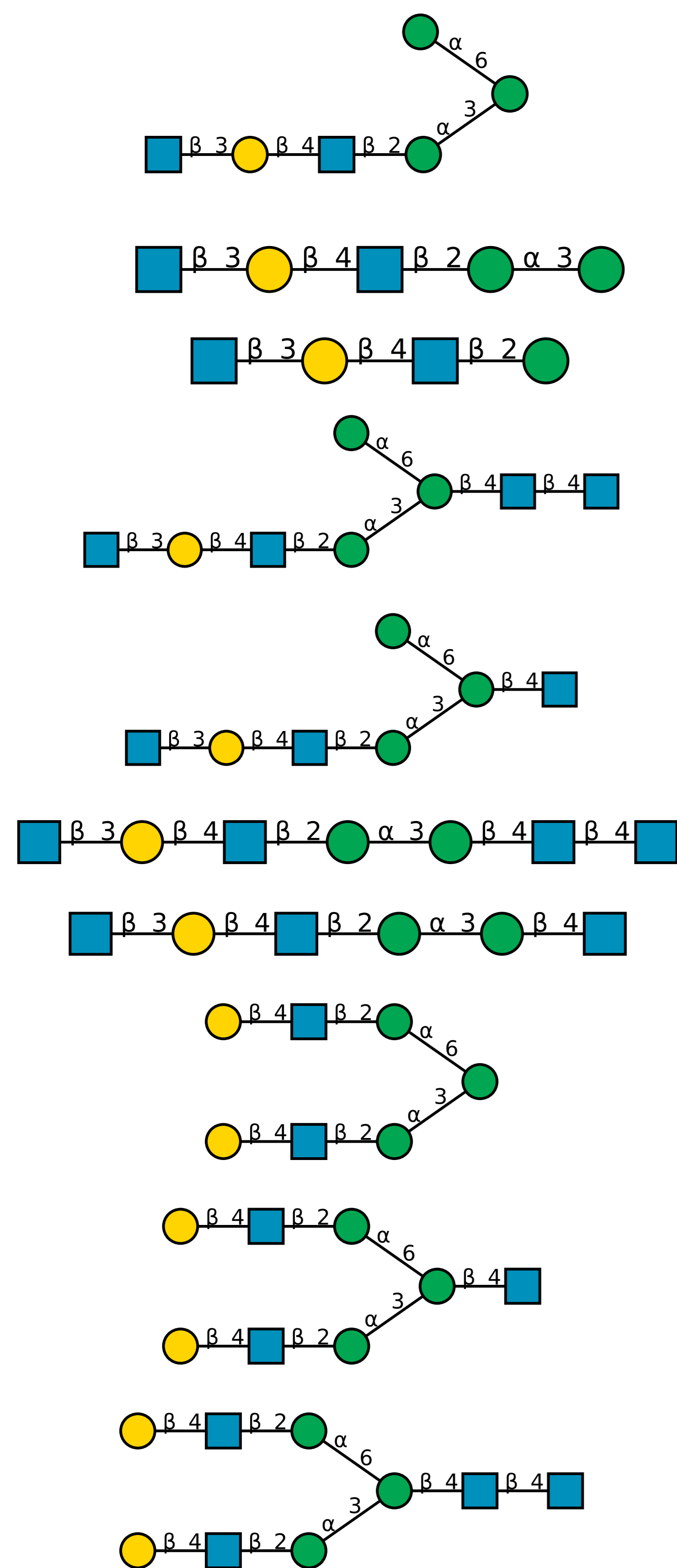

# GLYCAN MOTIF MINER MOTIFS

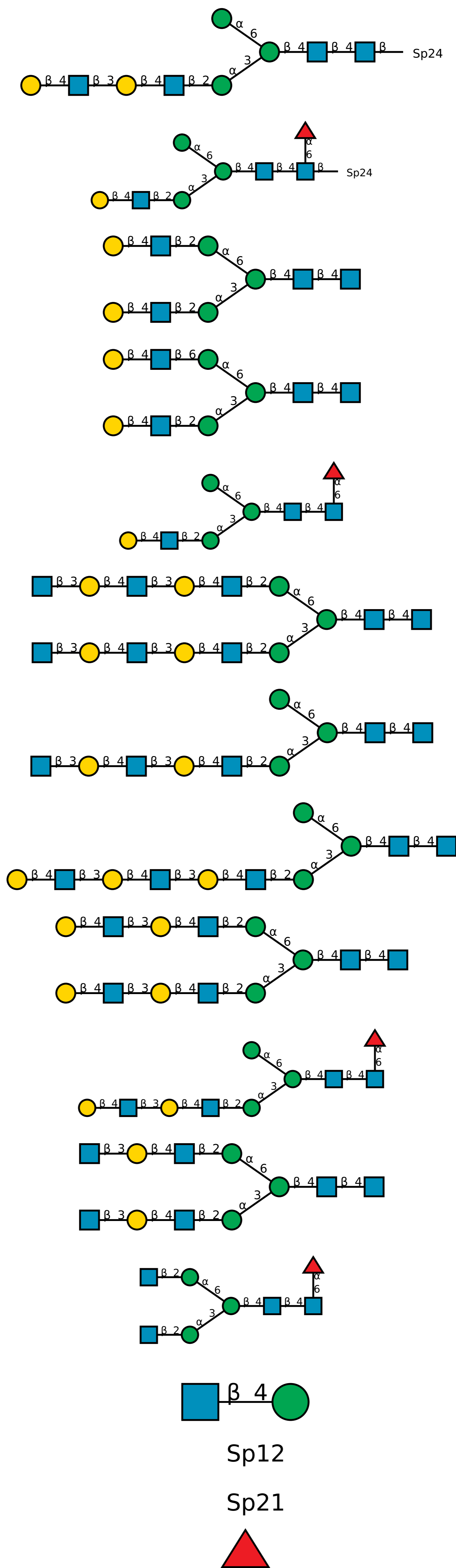

# CCARL MOTIFS

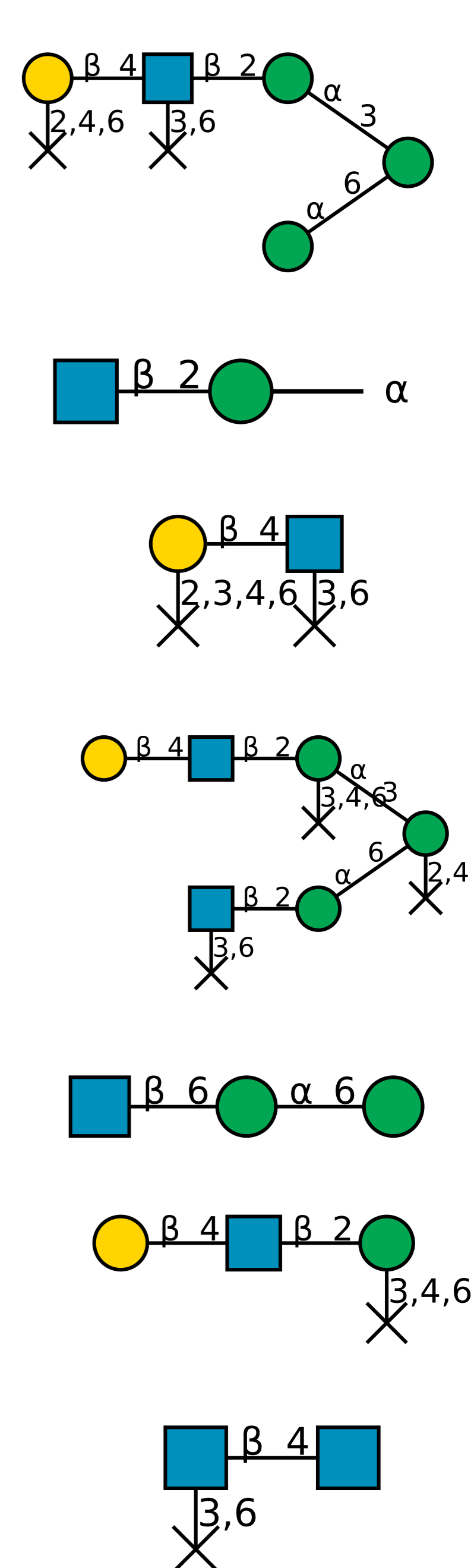

Supplement: Supplementary file 8 — Additional file 8 Motifs from GLYMMR and glycan motif miner. Motifs extracted using GLYMMR and Glycan Miner Tool for a range of glycan microarray datasets. [file 12859_2020_3374_MOESM8_ESM.zip › PHA-E.pdf]

GLYMMR MOTIF

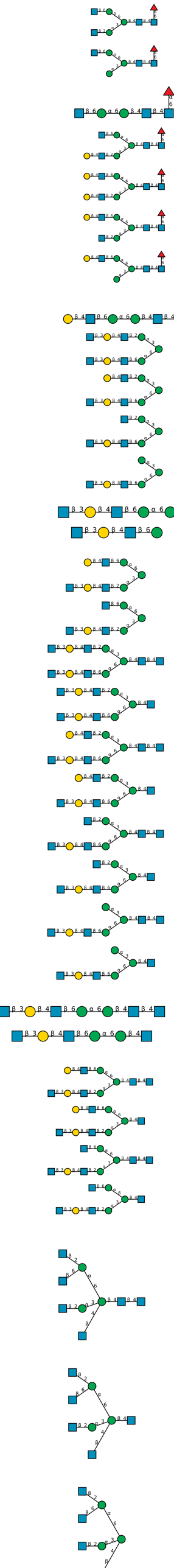

## GLYCAN MOTIF MINER MOTIFS

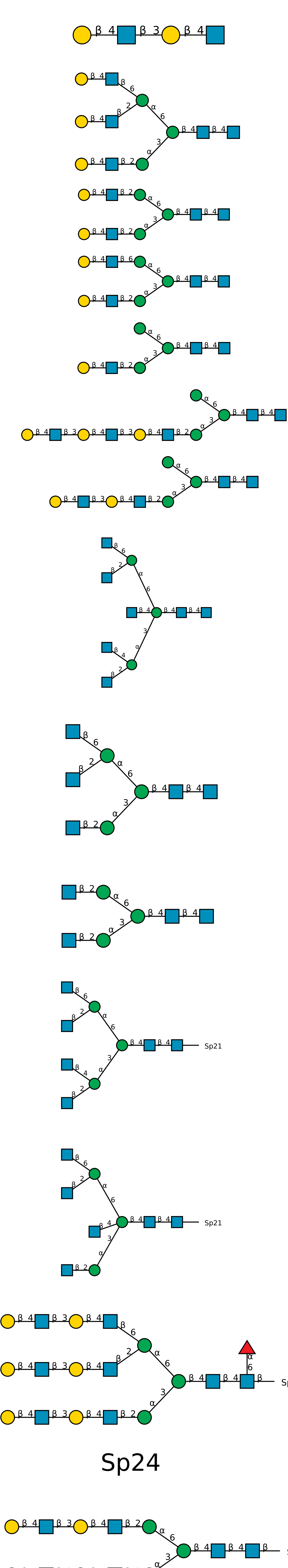

## CCARL MOTIFS

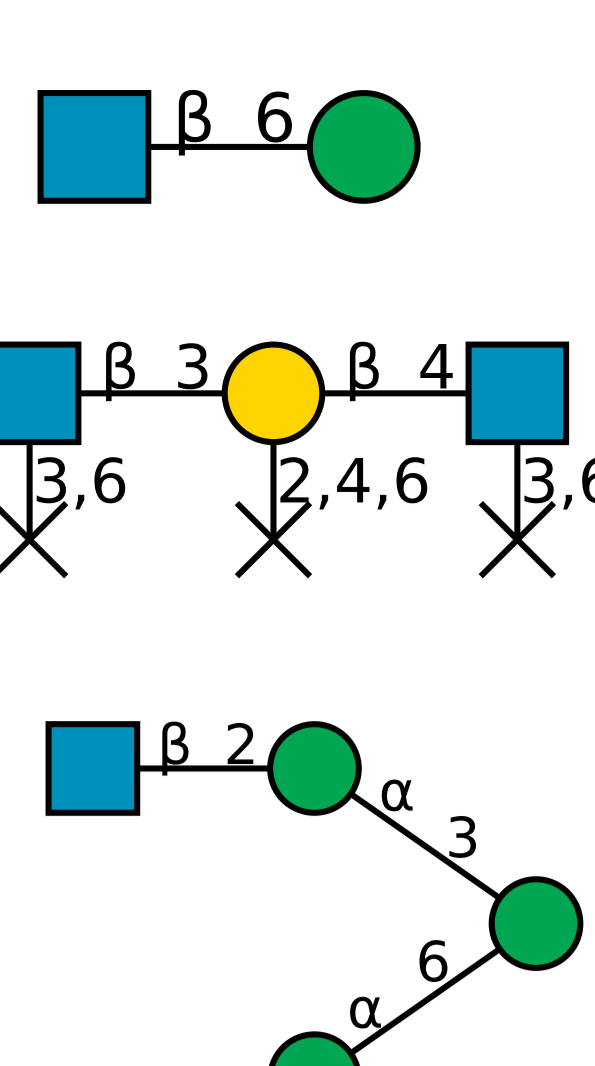

Supplement: Supplementary file 8 — Additional file 8 Motifs from GLYMMR and glycan motif miner. Motifs extracted using GLYMMR and Glycan Miner Tool for a range of glycan microarray datasets. [file 12859_2020_3374_MOESM8_ESM.zip › PHA-L.pdf]

## GLYMMR MOTIFS

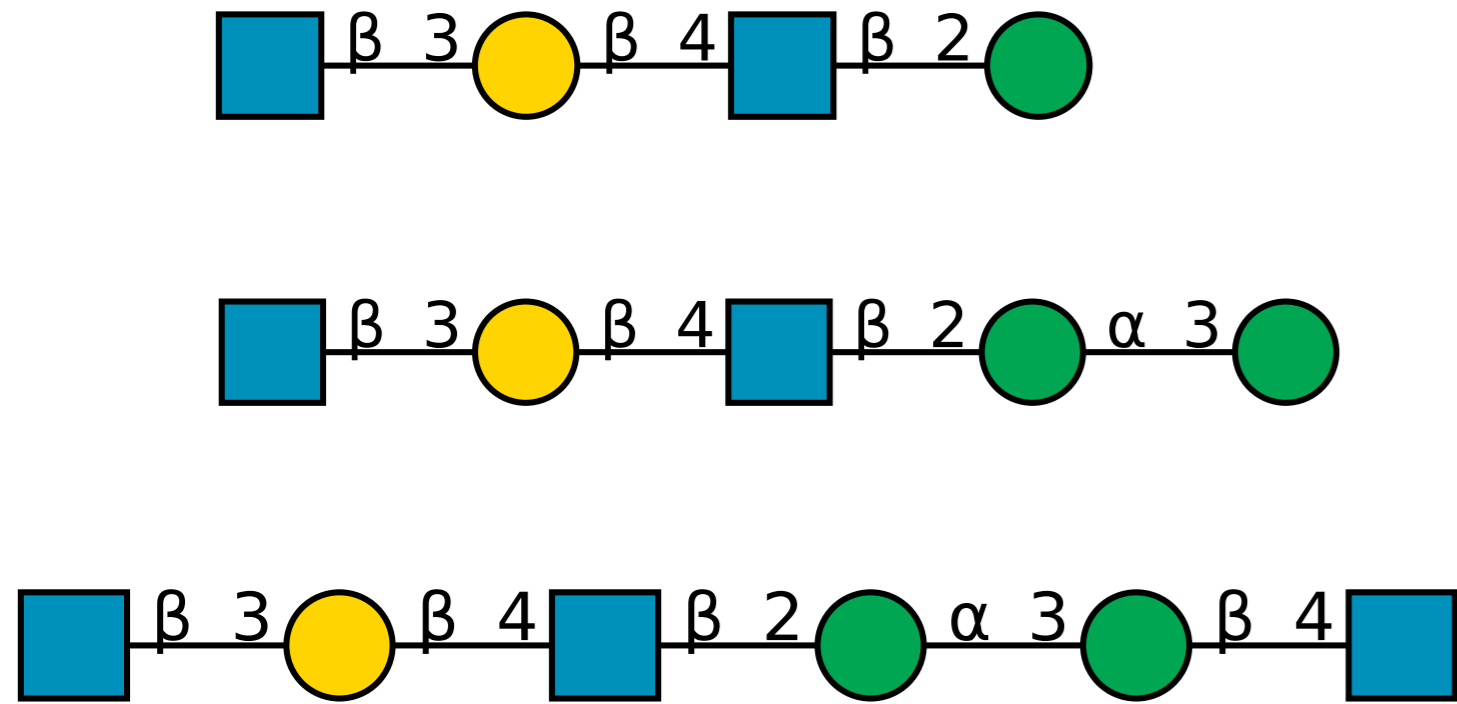

## GLYCAN MOTIF MINER MOTIFS

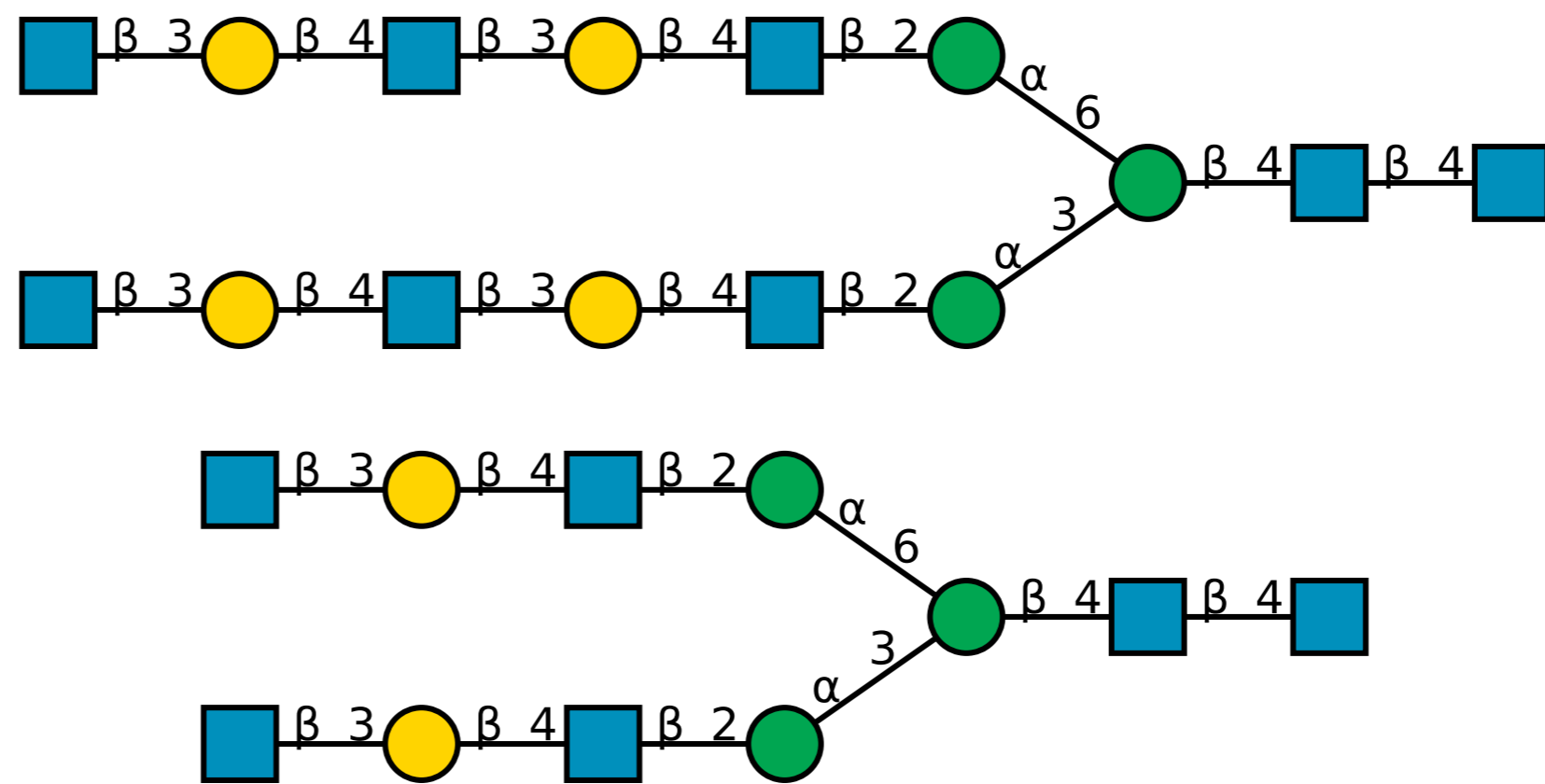

## CCARL MOTIFS

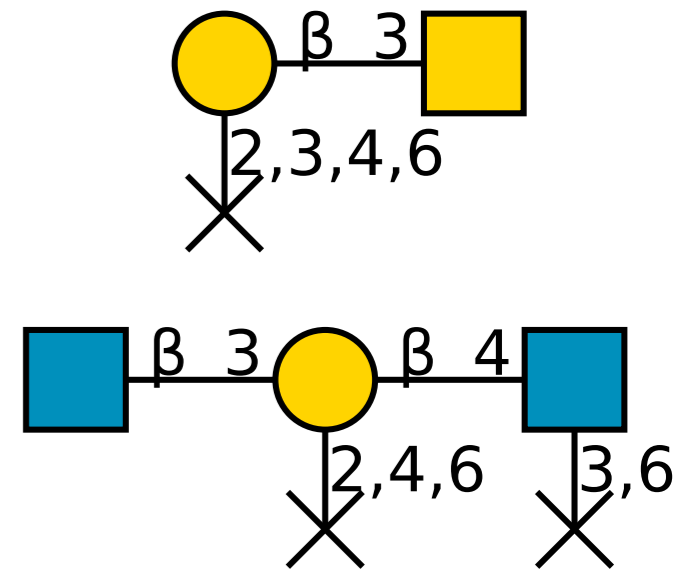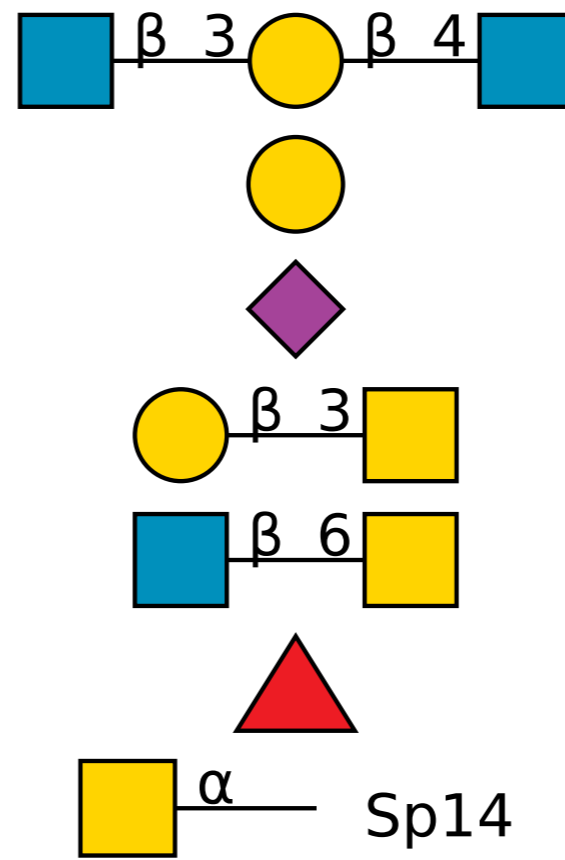

Sp12

Supplement: Supplementary file 8 — Additional file 8 Motifs from GLYMMR and glycan motif miner. Motifs extracted using GLYMMR and Glycan Miner Tool for a range of glycan microarray datasets. [file 12859_2020_3374_MOESM8_ESM.zip › PNA.pdf]

GLYMMR MOTIFS

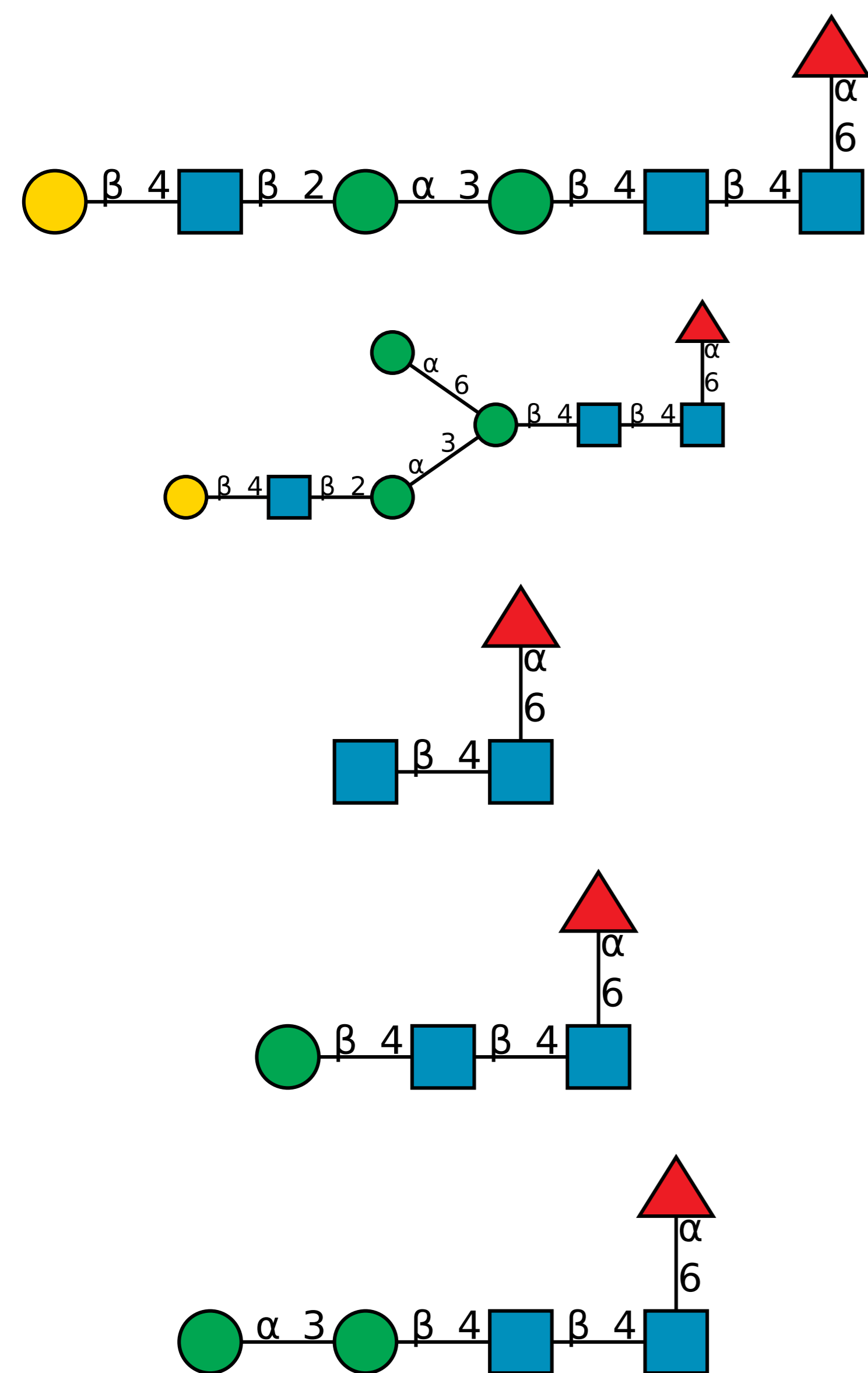

GLYCAN MOTIF MINER MOTIFS

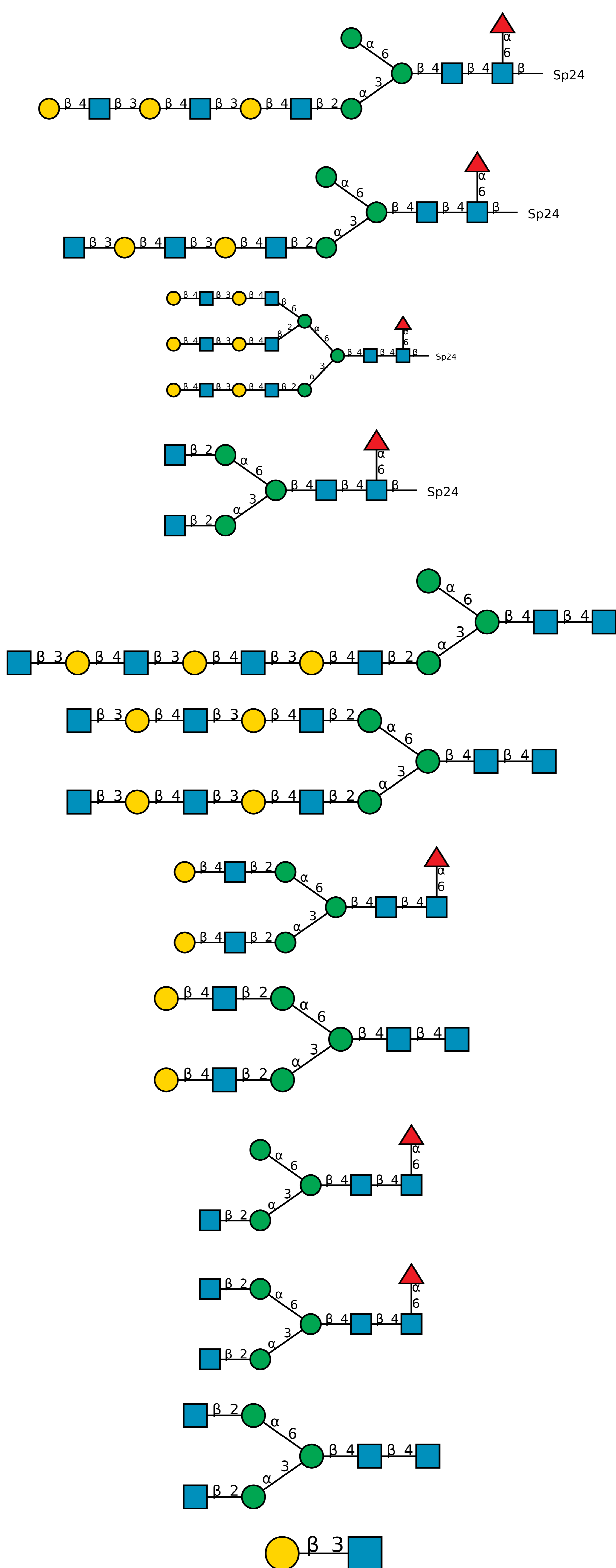

CCARL MOTIFS

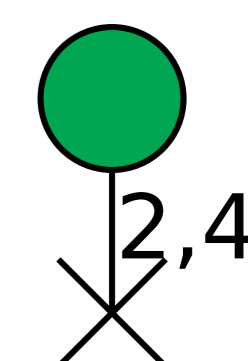

Supplement: Supplementary file 8 — Additional file 8 Motifs from GLYMMR and glycan motif miner. Motifs extracted using GLYMMR and Glycan Miner Tool for a range of glycan microarray datasets. [file 12859_2020_3374_MOESM8_ESM.zip › PSA.pdf]

GLYMMR MOTIFS

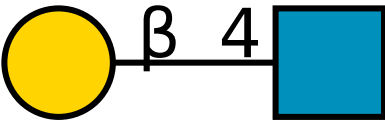

GLYCAN MOTIF MINER MOTIFS

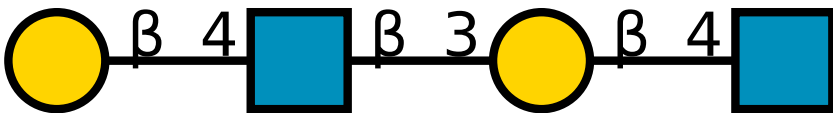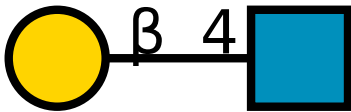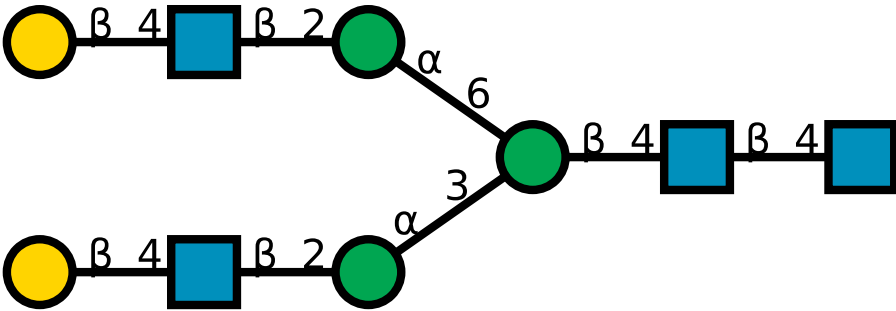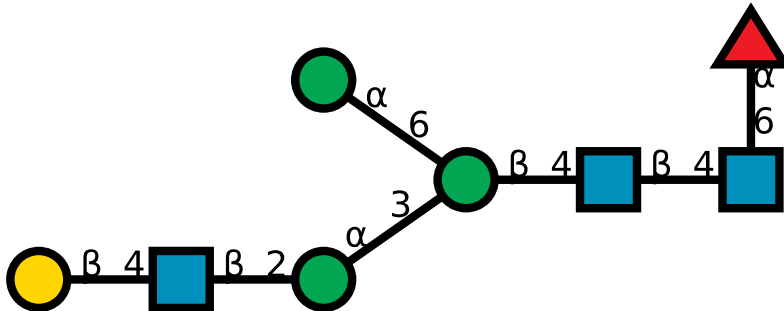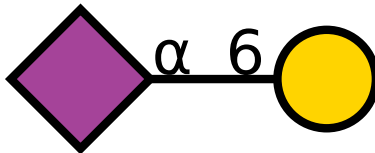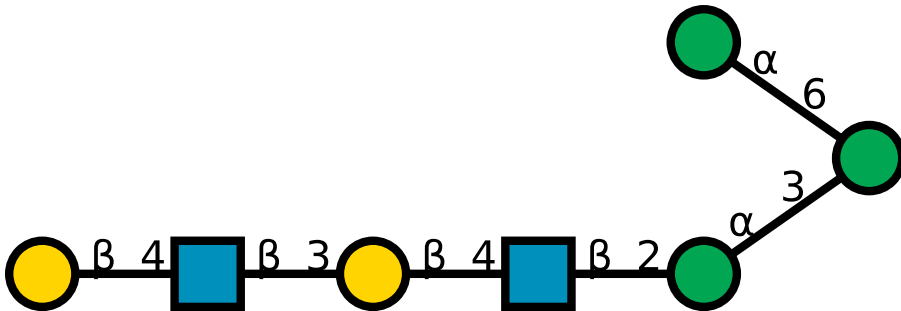

CCARL MOTIFS

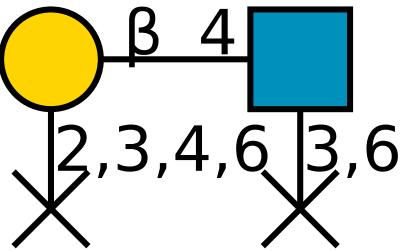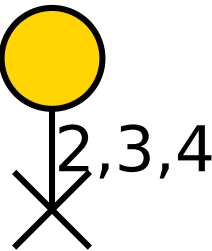

Sp0

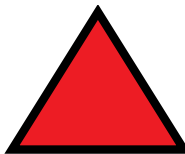

Sp21

Supplement: Supplementary file 8 — Additional file 8 Motifs from GLYMMR and glycan motif miner. Motifs extracted using GLYMMR and Glycan Miner Tool for a range of glycan microarray datasets. [file 12859_2020_3374_MOESM8_ESM.zip › RCA_I.pdf]

GLYMMR MOTIFS

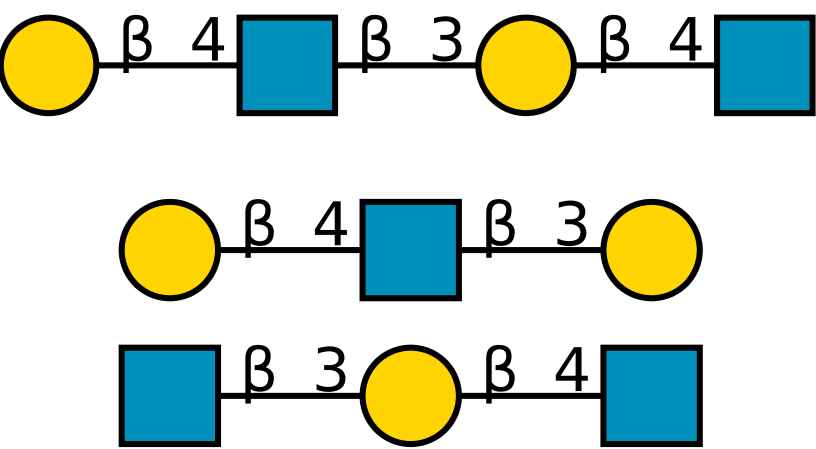

GLYCAN MOTIF MINER MOTIFS

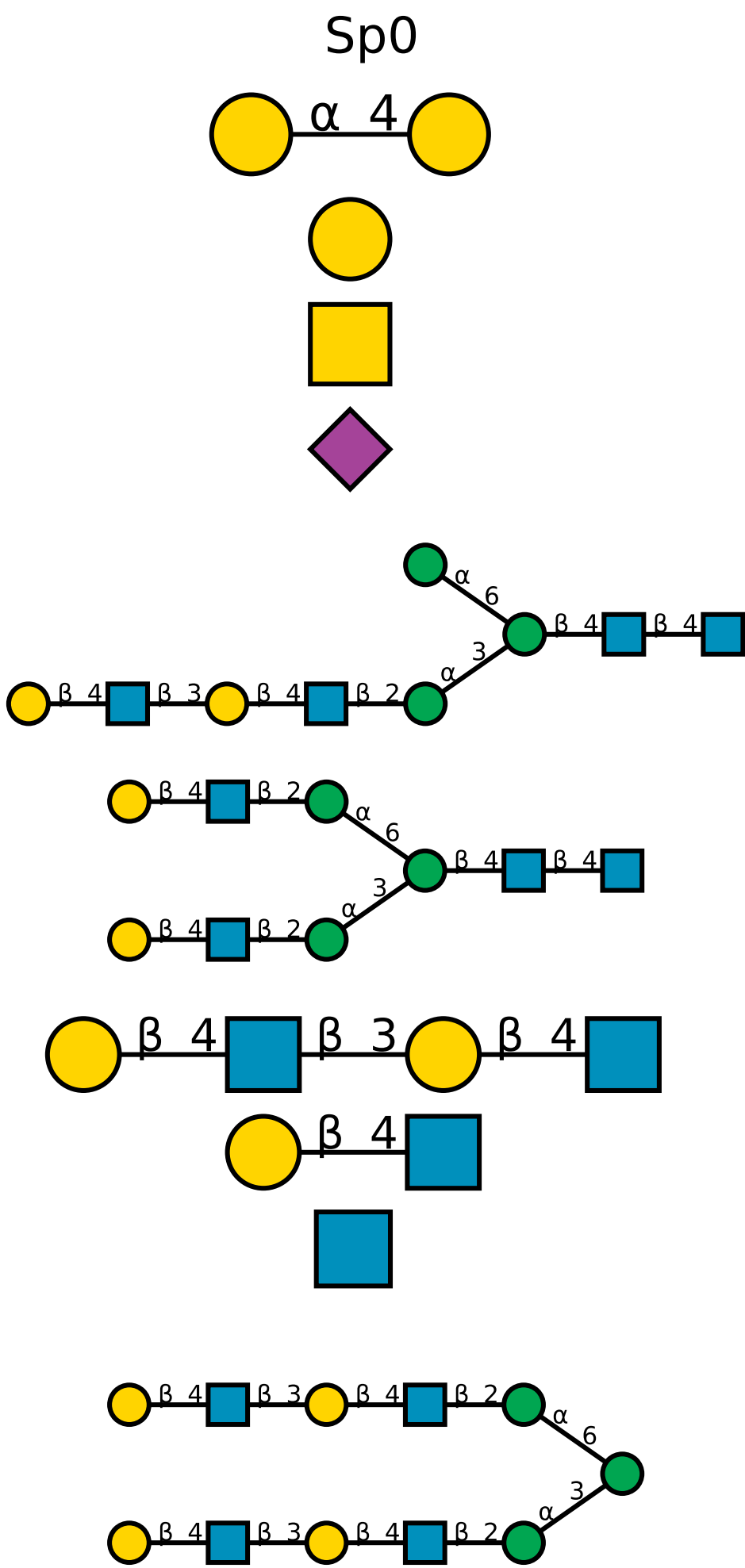

CCARL MOTIFS

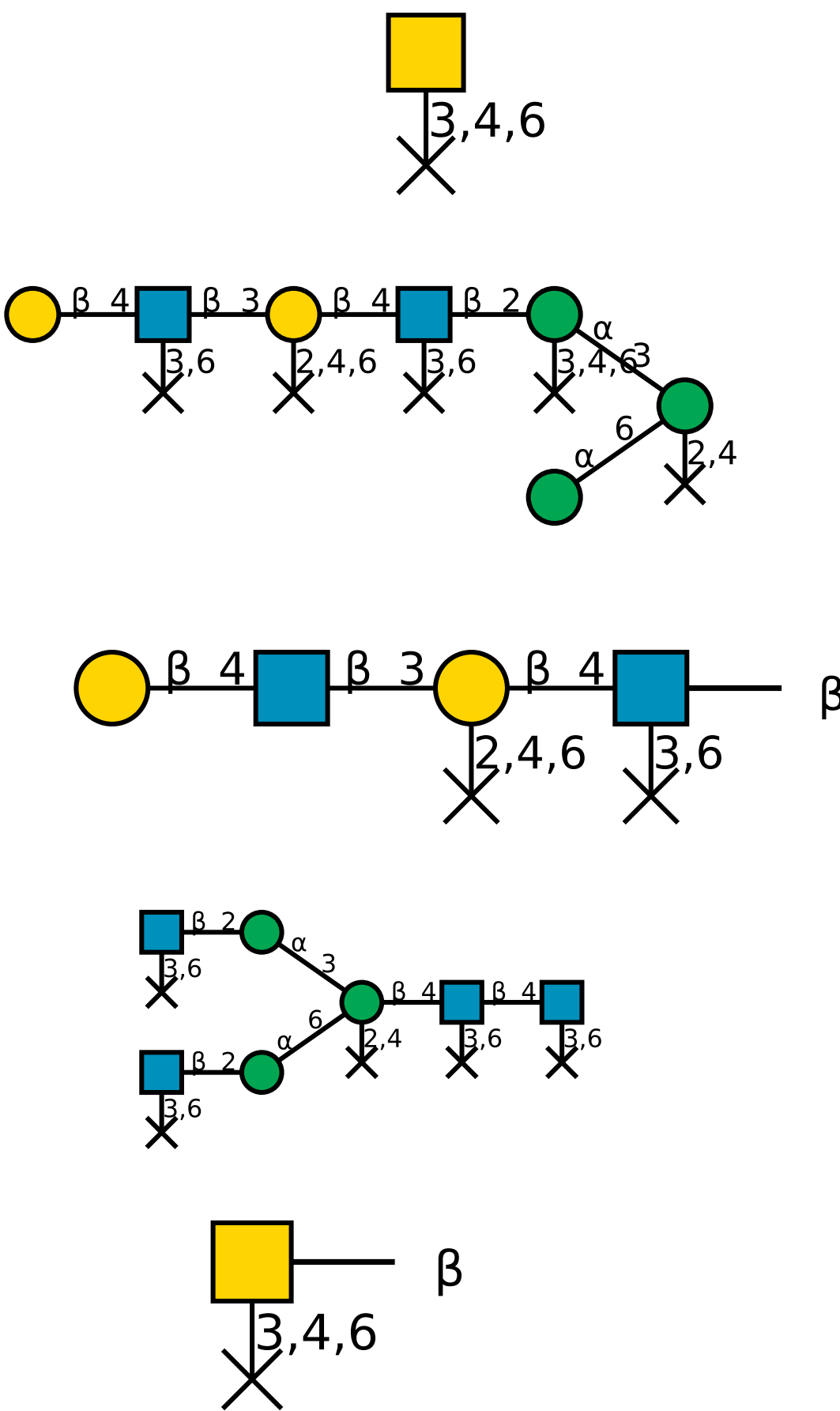

Supplement: Supplementary file 8 — Additional file 8 Motifs from GLYMMR and glycan motif miner. Motifs extracted using GLYMMR and Glycan Miner Tool for a range of glycan microarray datasets. [file 12859_2020_3374_MOESM8_ESM.zip › SBA.pdf]

GLYMMR MOTIFS

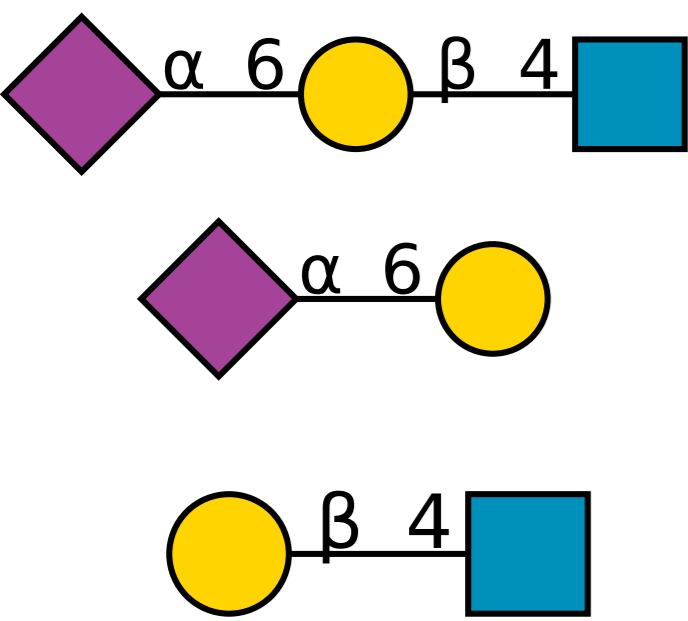

GLYCAN MOTIF MINER MOTIFS

Sp21

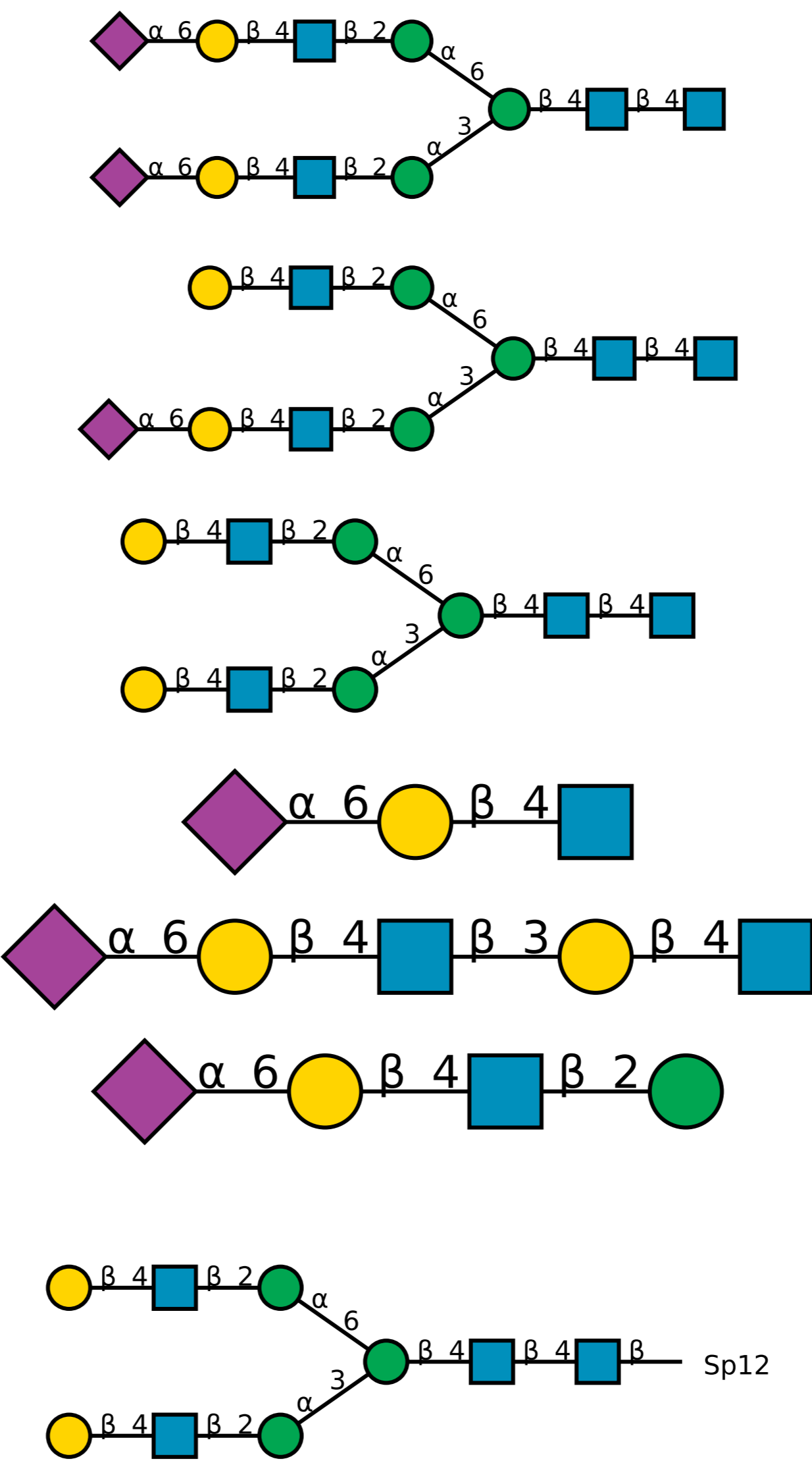

Sp12

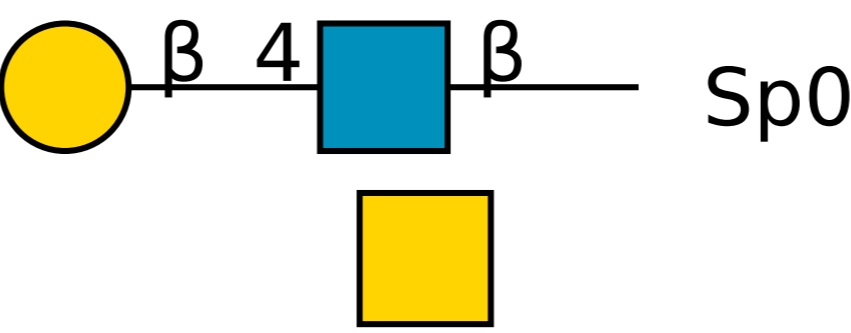

CCARL MOTIFS

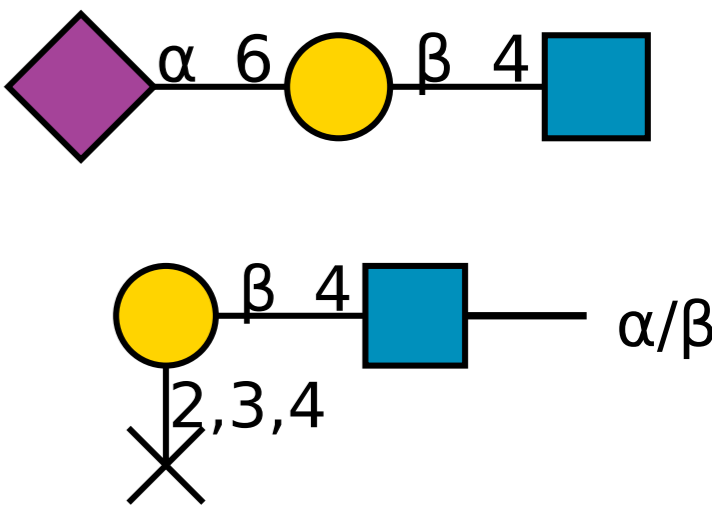

Supplement: Supplementary file 8 — Additional file 8 Motifs from GLYMMR and glycan motif miner. Motifs extracted using GLYMMR and Glycan Miner Tool for a range of glycan microarray datasets. [file 12859_2020_3374_MOESM8_ESM.zip › SNA.pdf]

GLYMMR MOTIFS

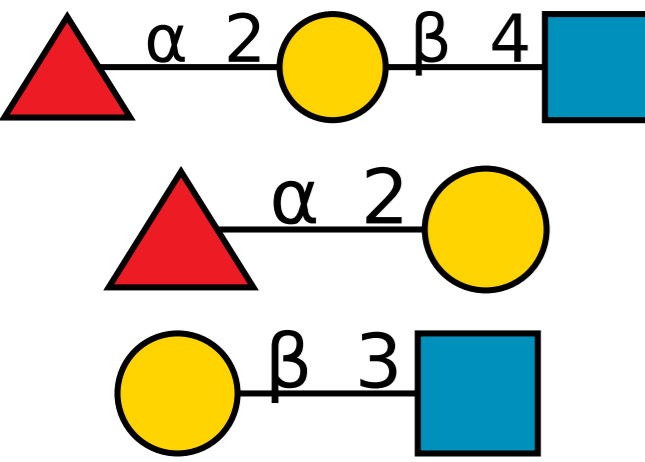

GLYCAN MOTIF MINER MOTIFS

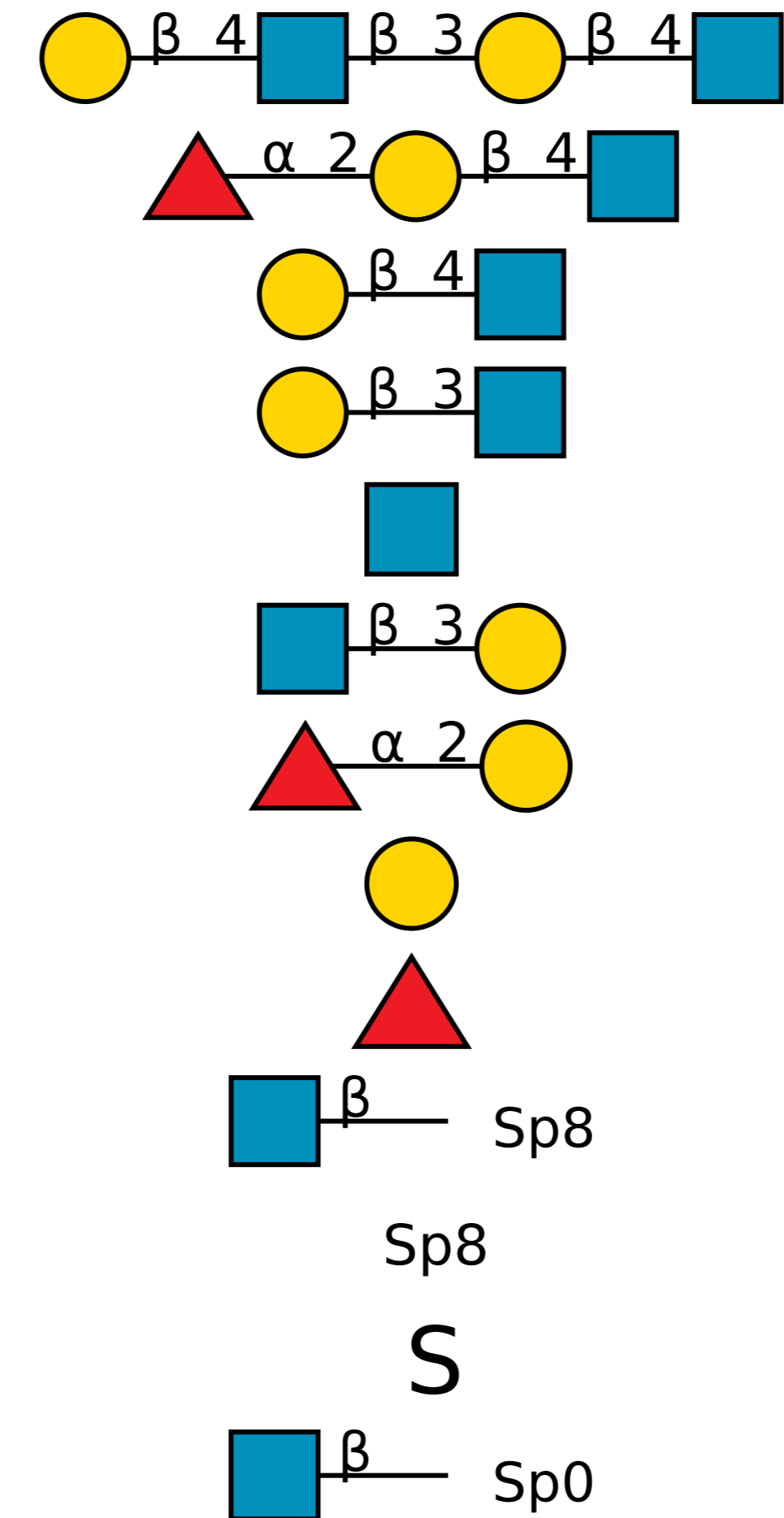

CCARL MOTIFS

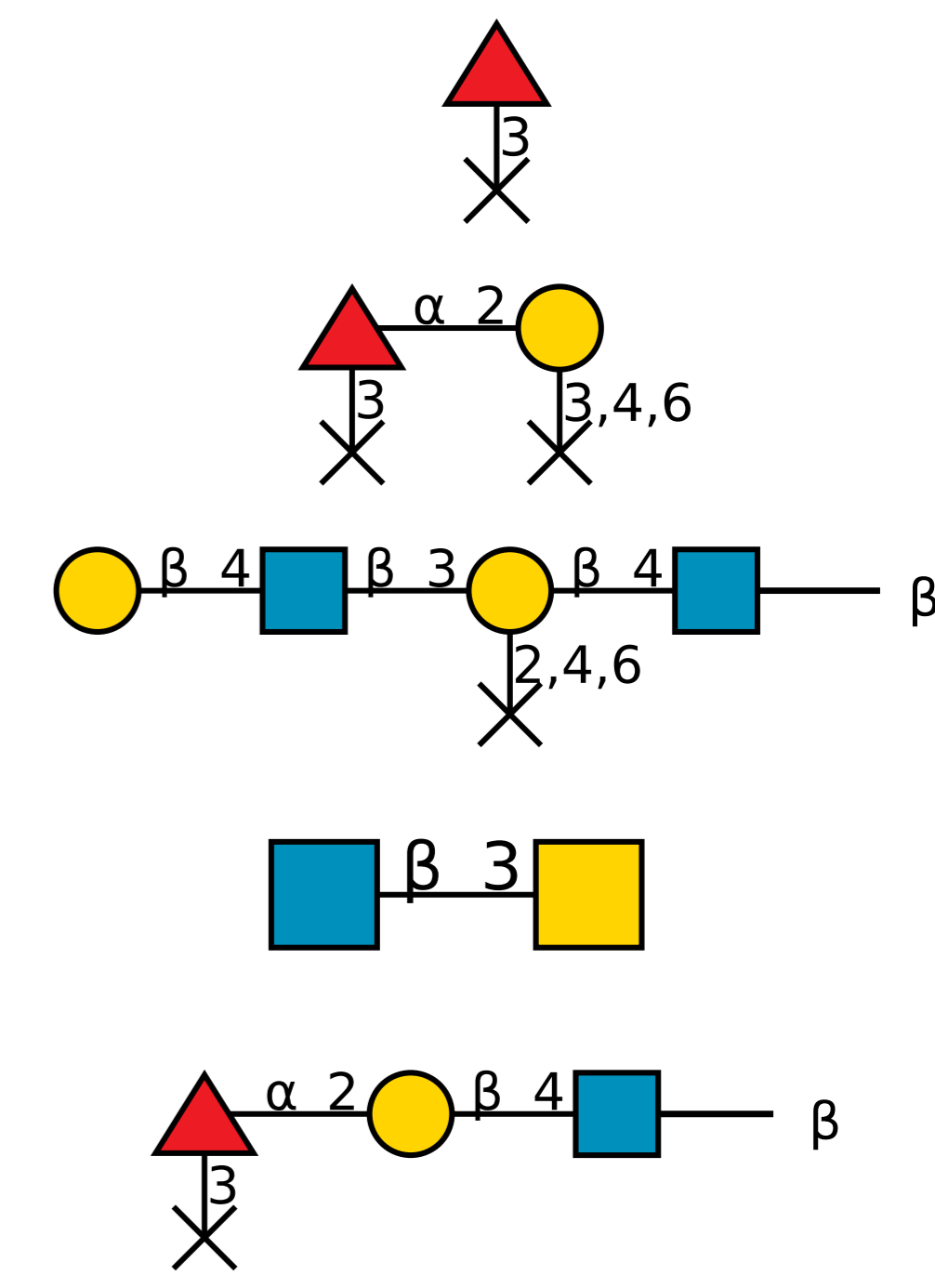

Supplement: Supplementary file 8 — Additional file 8 Motifs from GLYMMR and glycan motif miner. Motifs extracted using GLYMMR and Glycan Miner Tool for a range of glycan microarray datasets. [file 12859_2020_3374_MOESM8_ESM.zip › UEA1.pdf]

GLYMMR MOTIFS

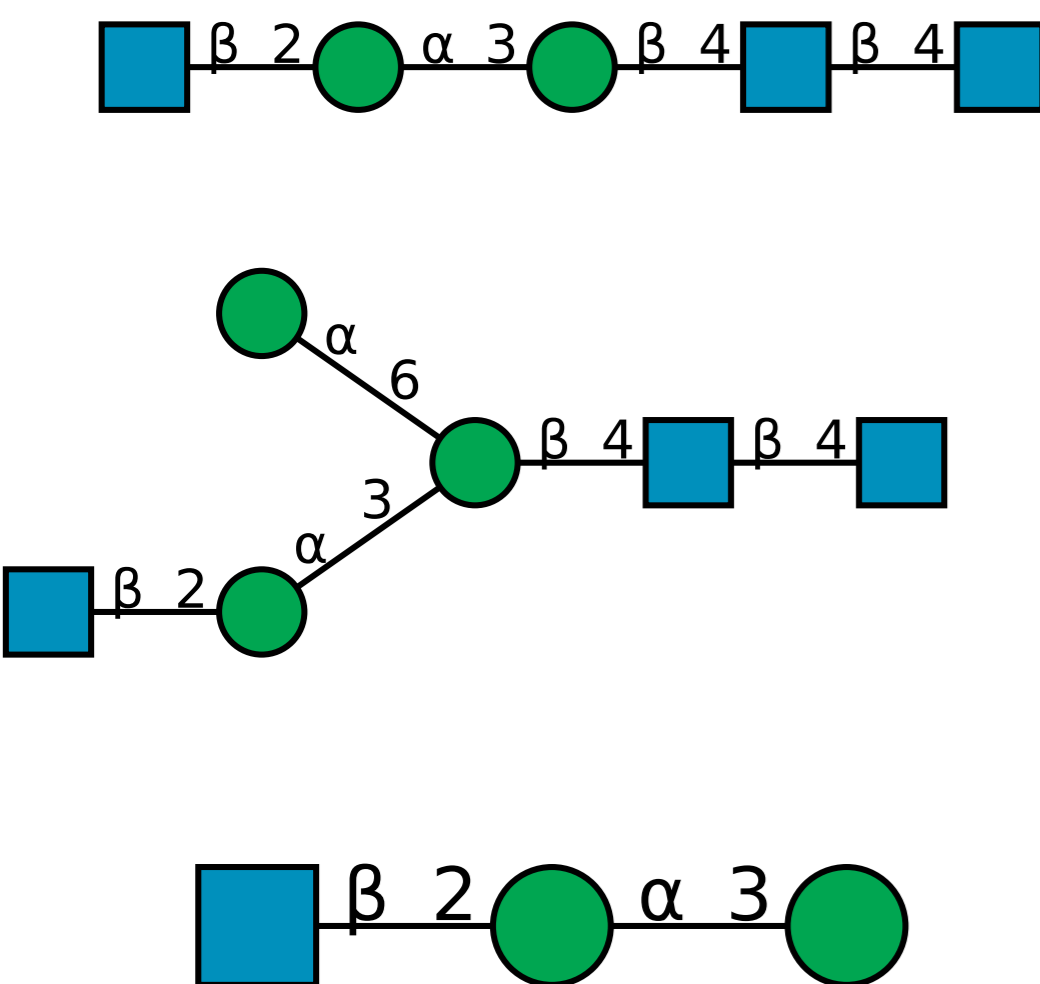

GLYCAN MOTIF MINER MOTIFS

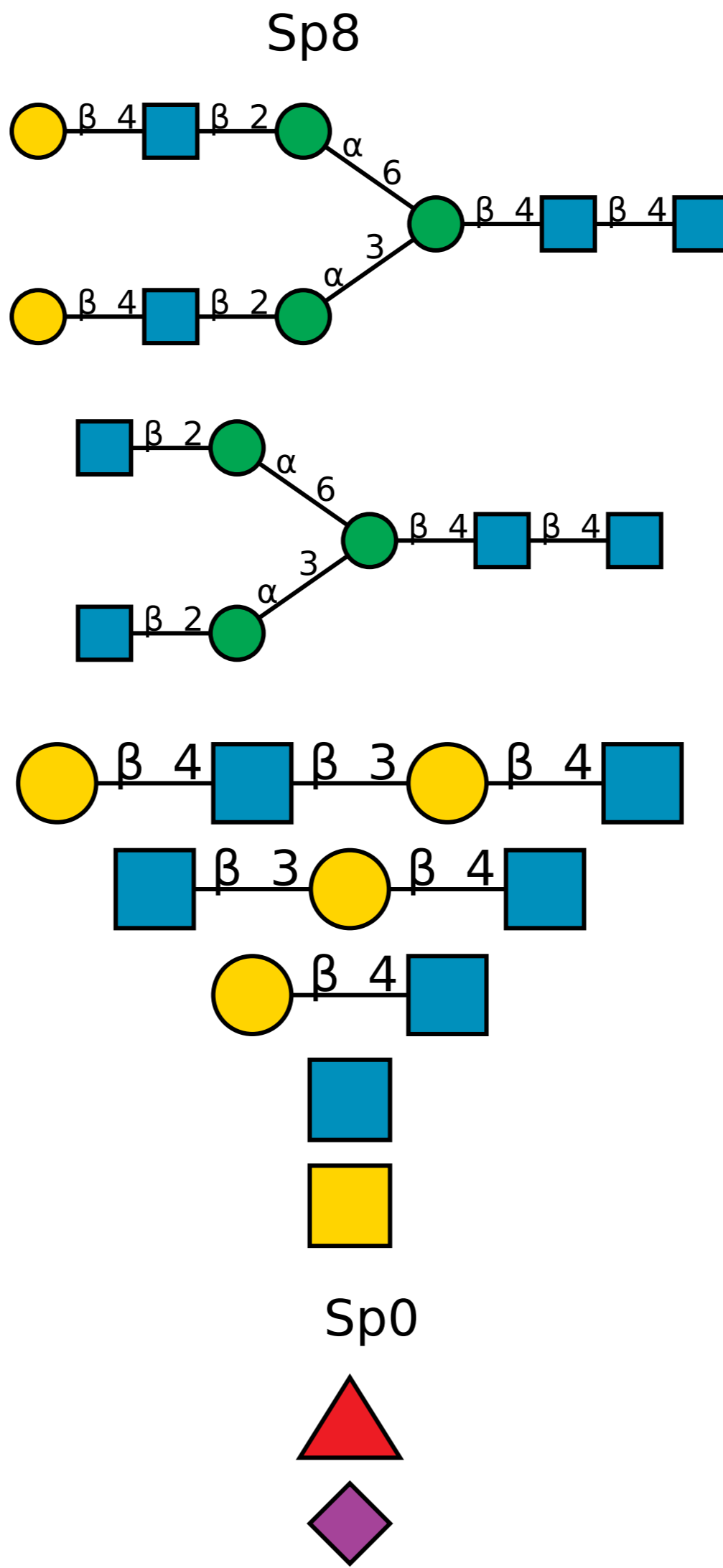

CCARL MOTIFS

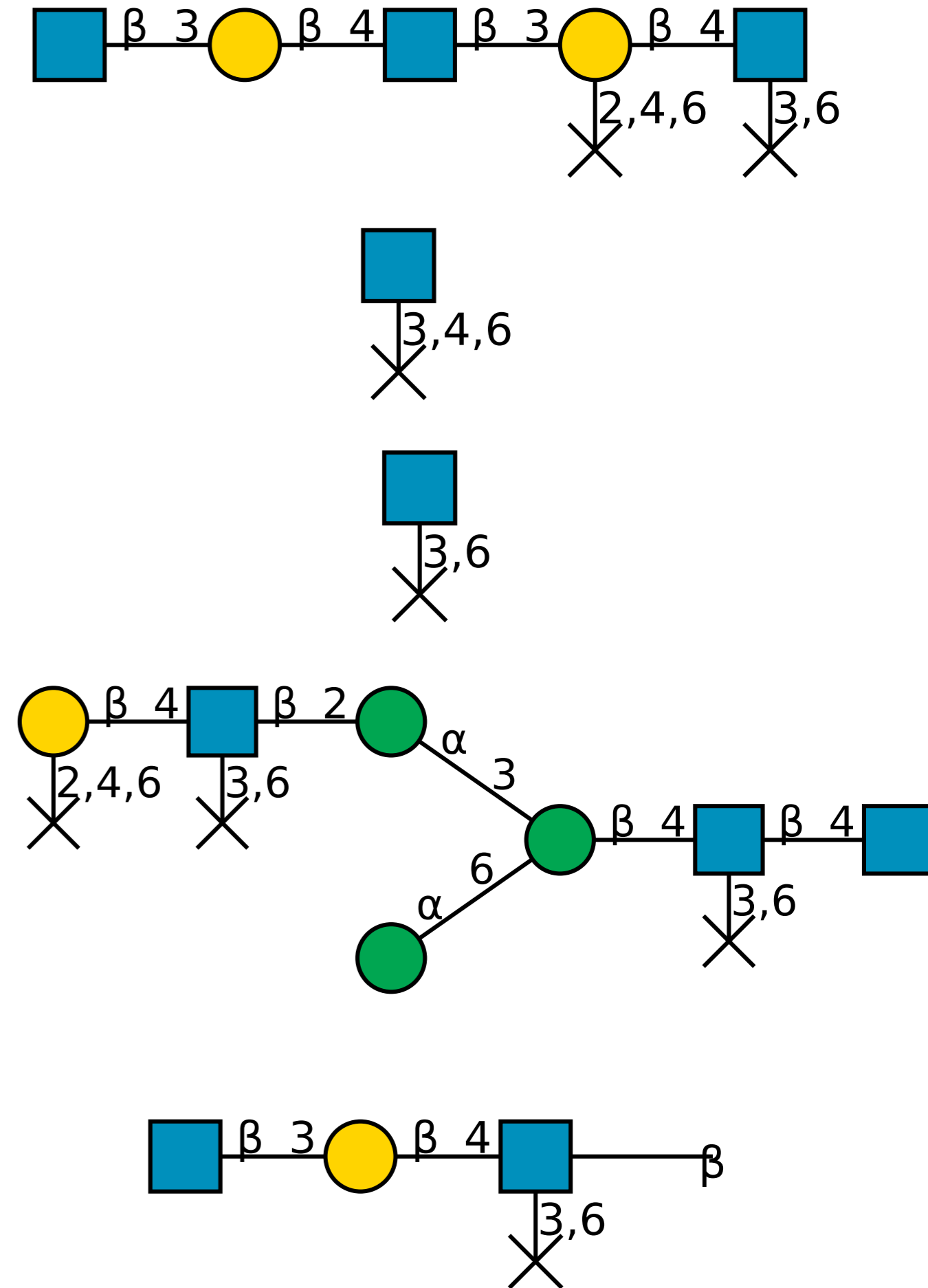

Supplement: Supplementary file 8 — Additional file 8 Motifs from GLYMMR and glycan motif miner. Motifs extracted using GLYMMR and Glycan Miner Tool for a range of glycan microarray datasets. [file 12859_2020_3374_MOESM8_ESM.zip › WGA.pdf]
