# Supplementary material for: Identifying glycan motifs using a novel subtree mining approach
Source: BMC Bioinformatics. 2020 Feb 4;21:42. doi: 10.1186/s12859-020-3374-4 (PMC7001330; doi:10.1186/s12859-020-3374-4)

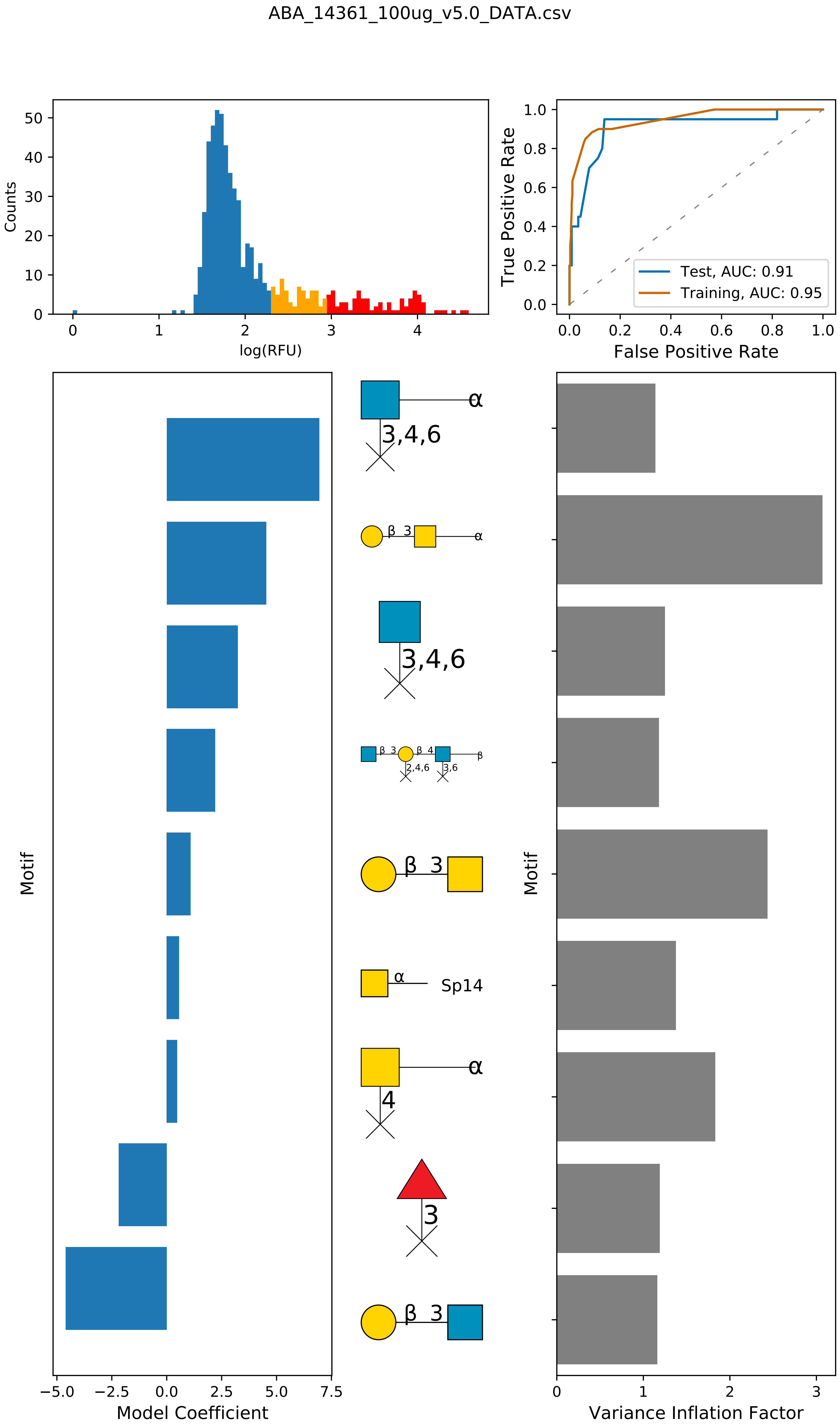
ABA_14361_100ug_v5.0_DATA.csv
Counts
log(RFU)
True Positive Rate
False Positive Rate
Test, AUC: 0.91
Training, AUC: 0.95
Motif
Model Coefficient
Variance Inflation Factor
Sp14

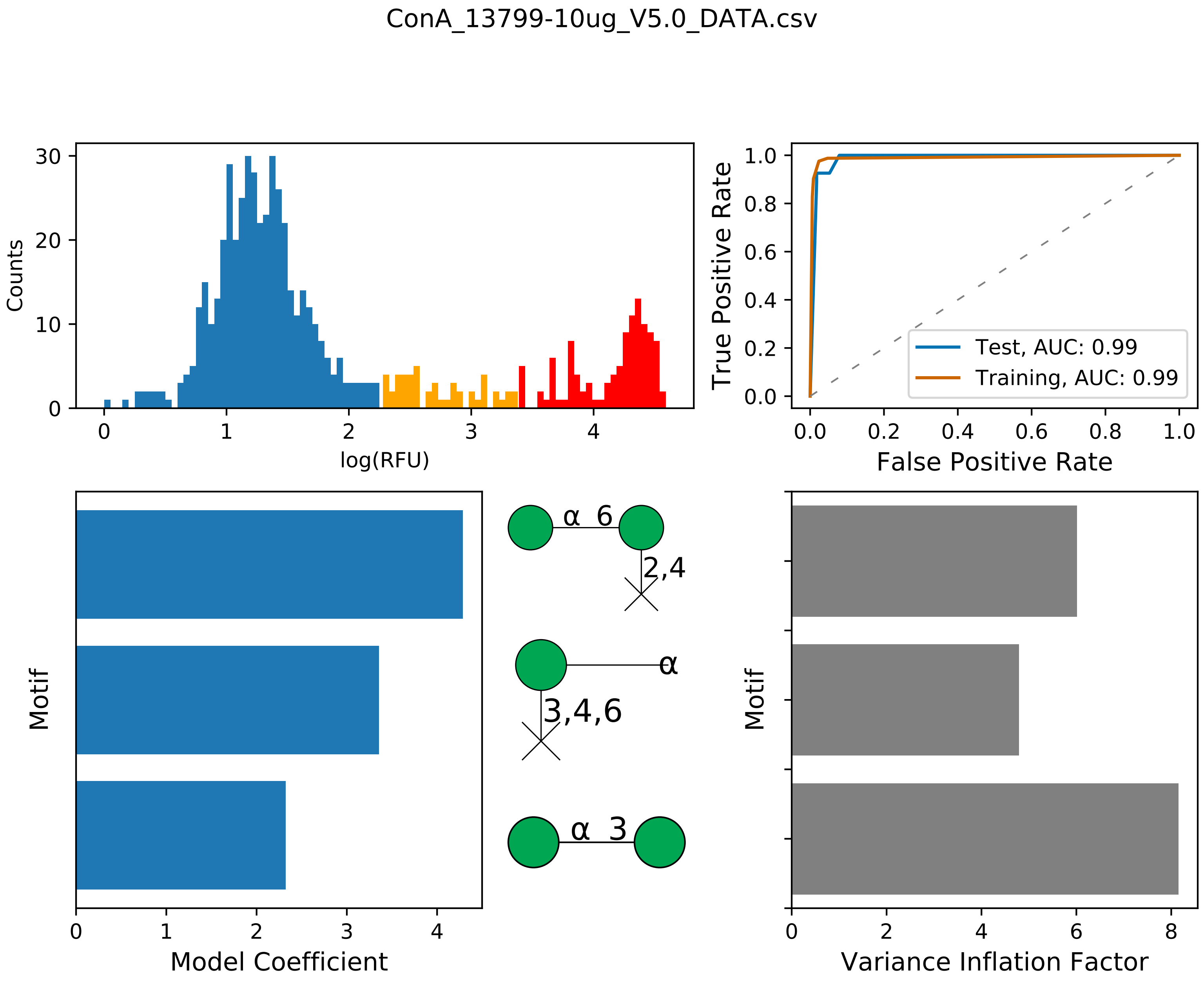

ConA_13799-10ug_V5.0_DATA.csv
Counts
log(RFU)
True Positive Rate
False Positive Rate
Test, AUC: 0.99
Training, AUC: 0.99
Motif
Model Coefficient
α 6
2,4
α
3,4,6
α 3
Motif
Variance Inflation Factor

PNA_14030_10ug_v5.0_DATA.csv

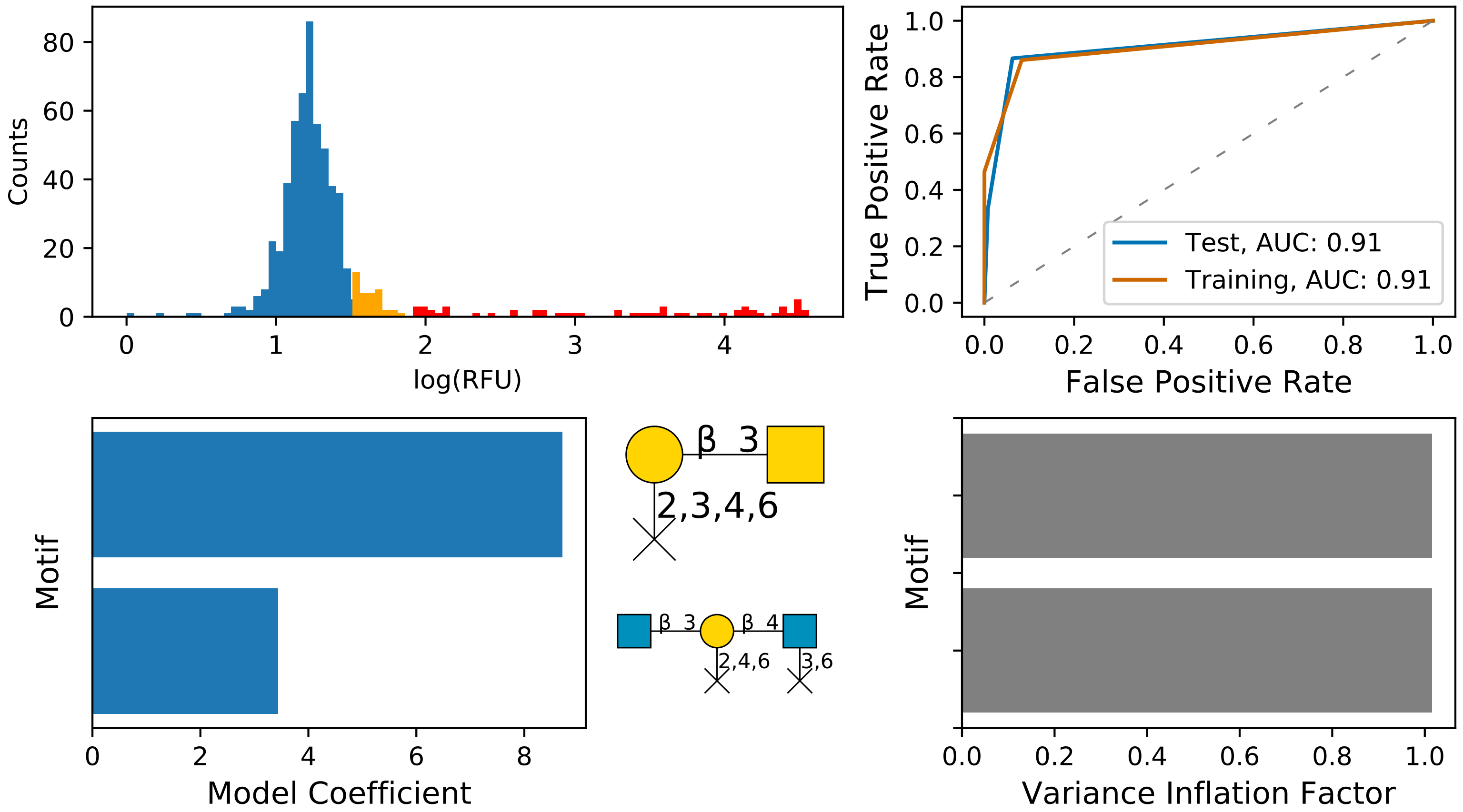

RCAI_10ug_14110_v5.0_DATA.csv

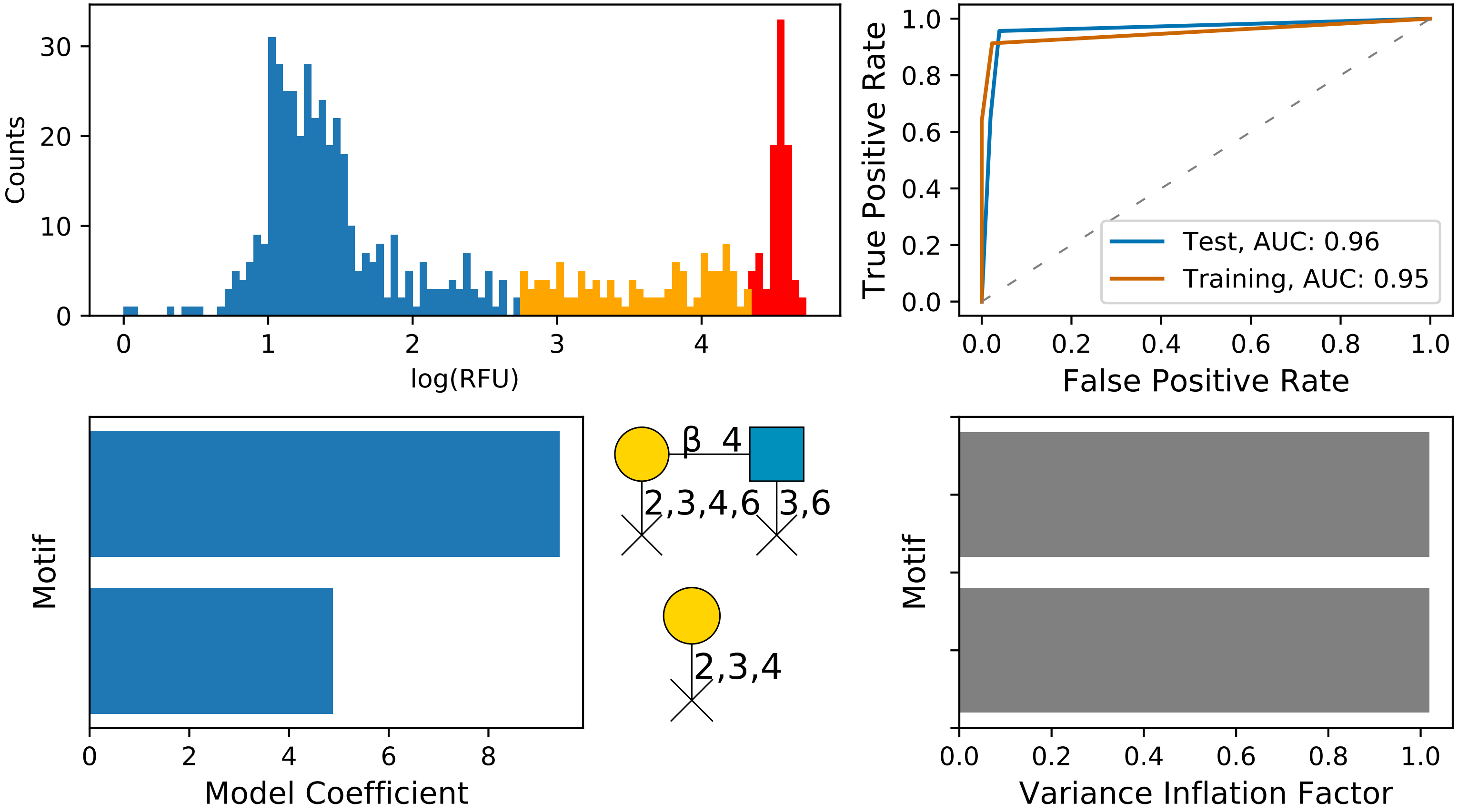

PHA-E-10ug_13853_V5.0_DATA.csv

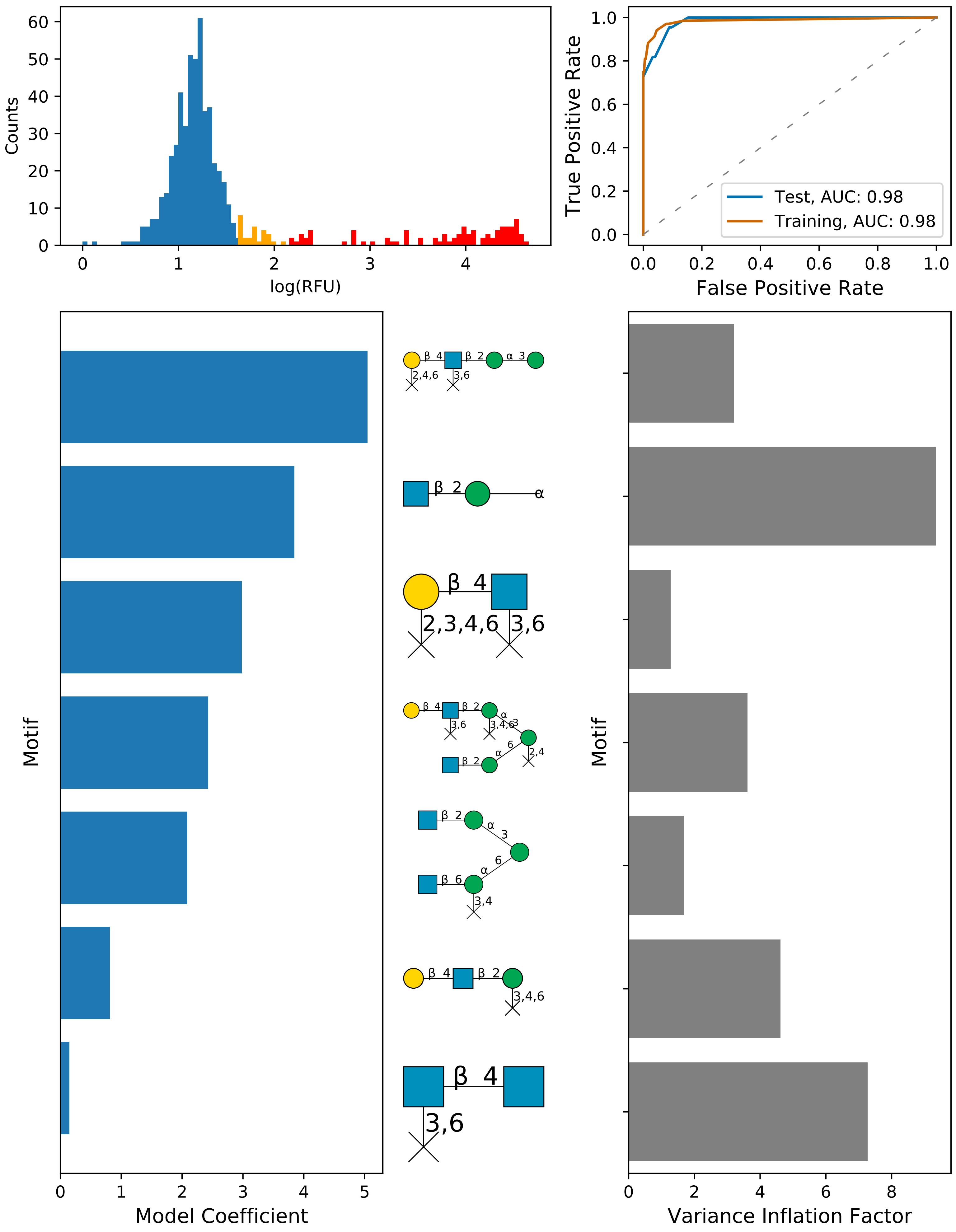

PHA-L-10ug_13856_V5.0_DATA.csv

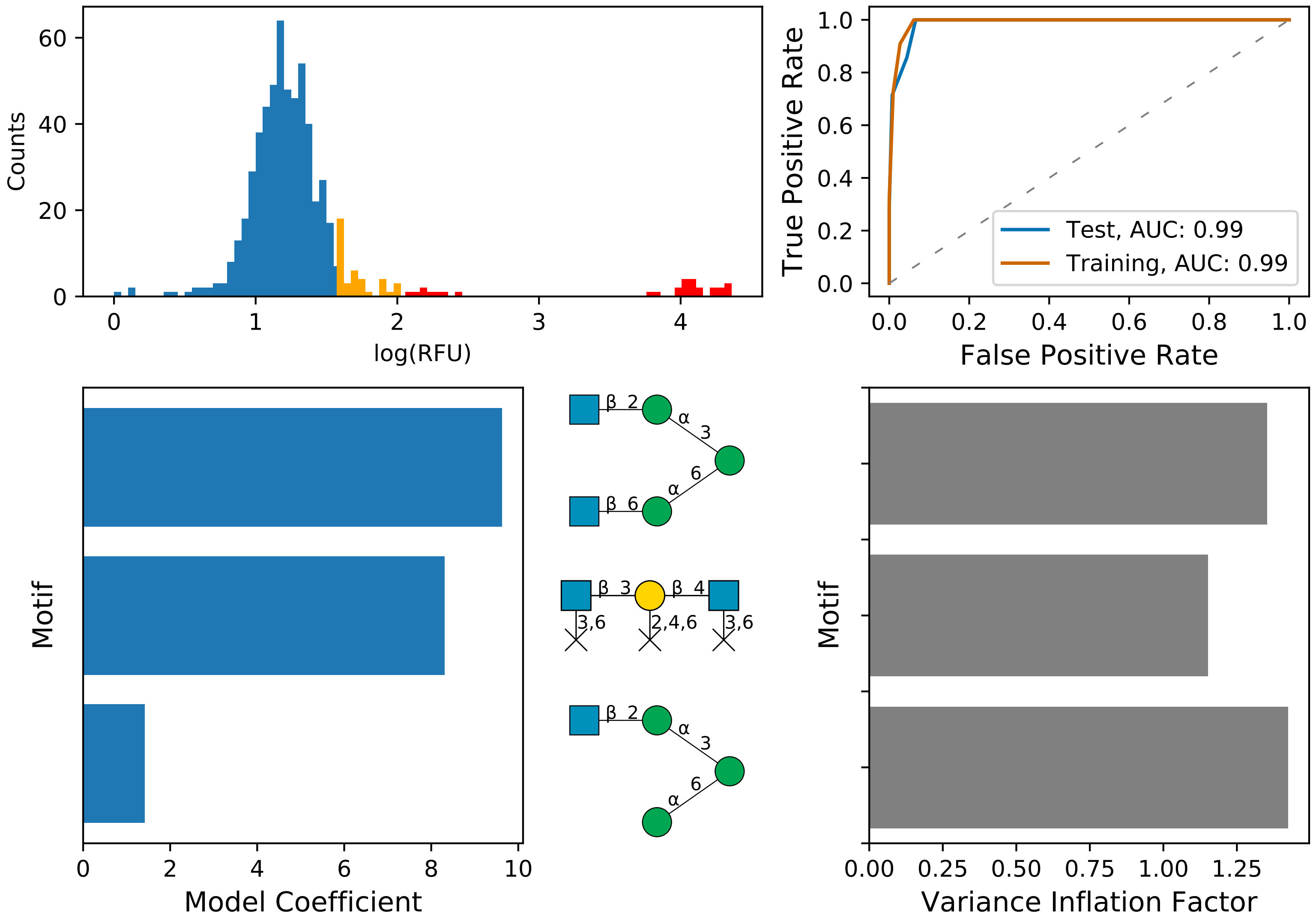

LCA_10ug_13934_v5.0_DATA.csv

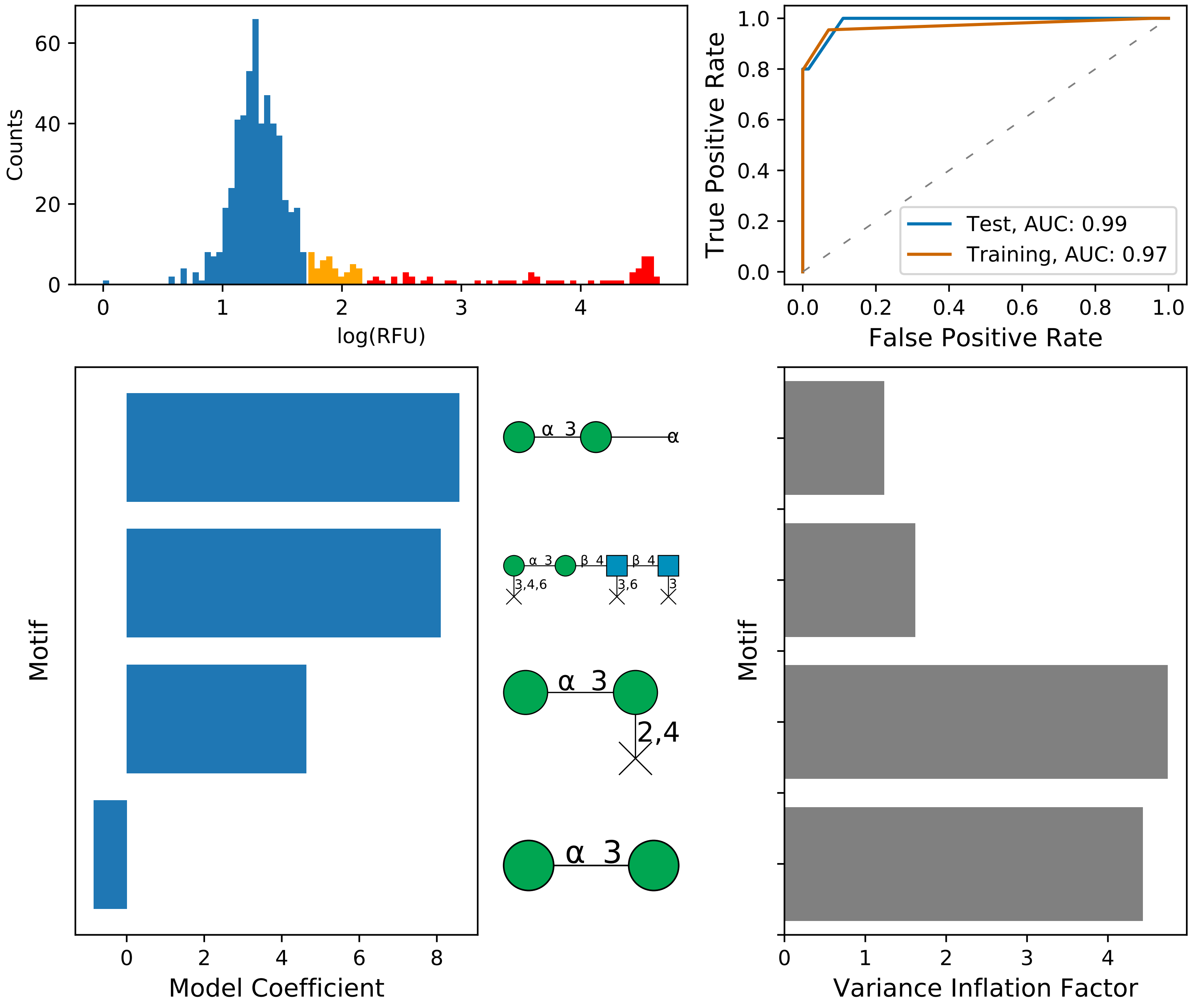

SNA_10ug_13631_v5.0_DATA.csv

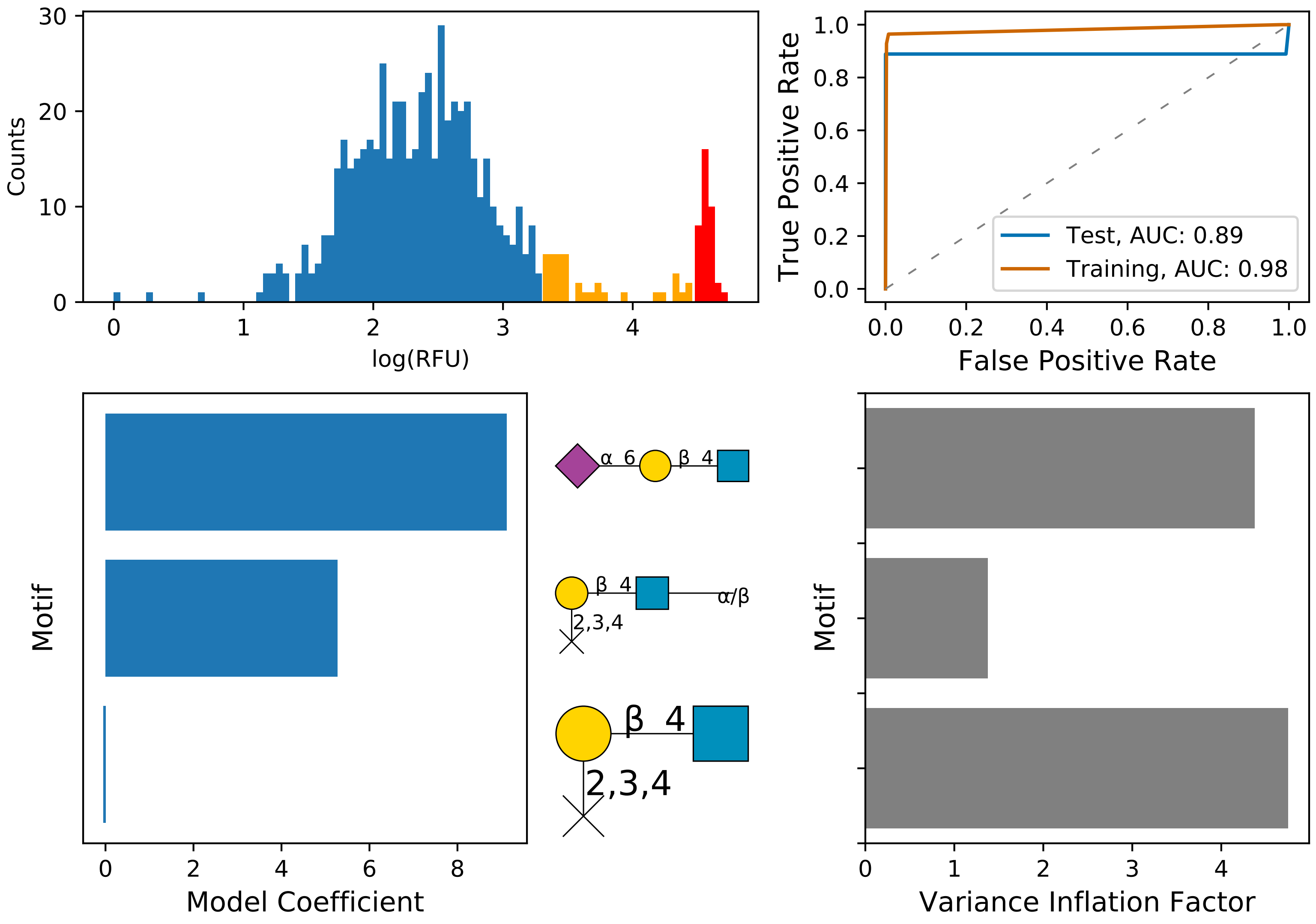

MAL-I_10ug_13883_v5.0_DATA.csv

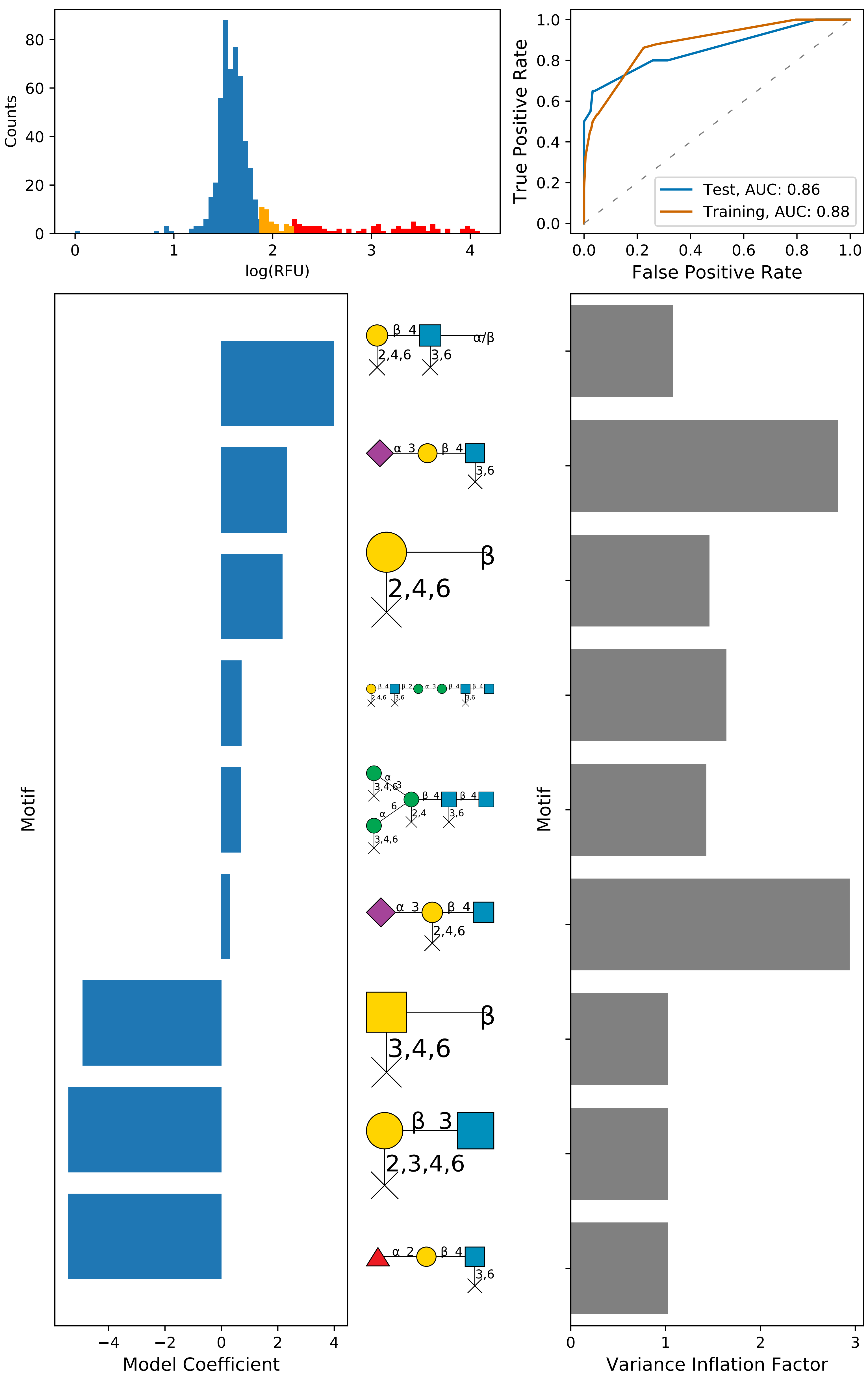

MAL_II_10ug_13886_v5.0_DATA.csv

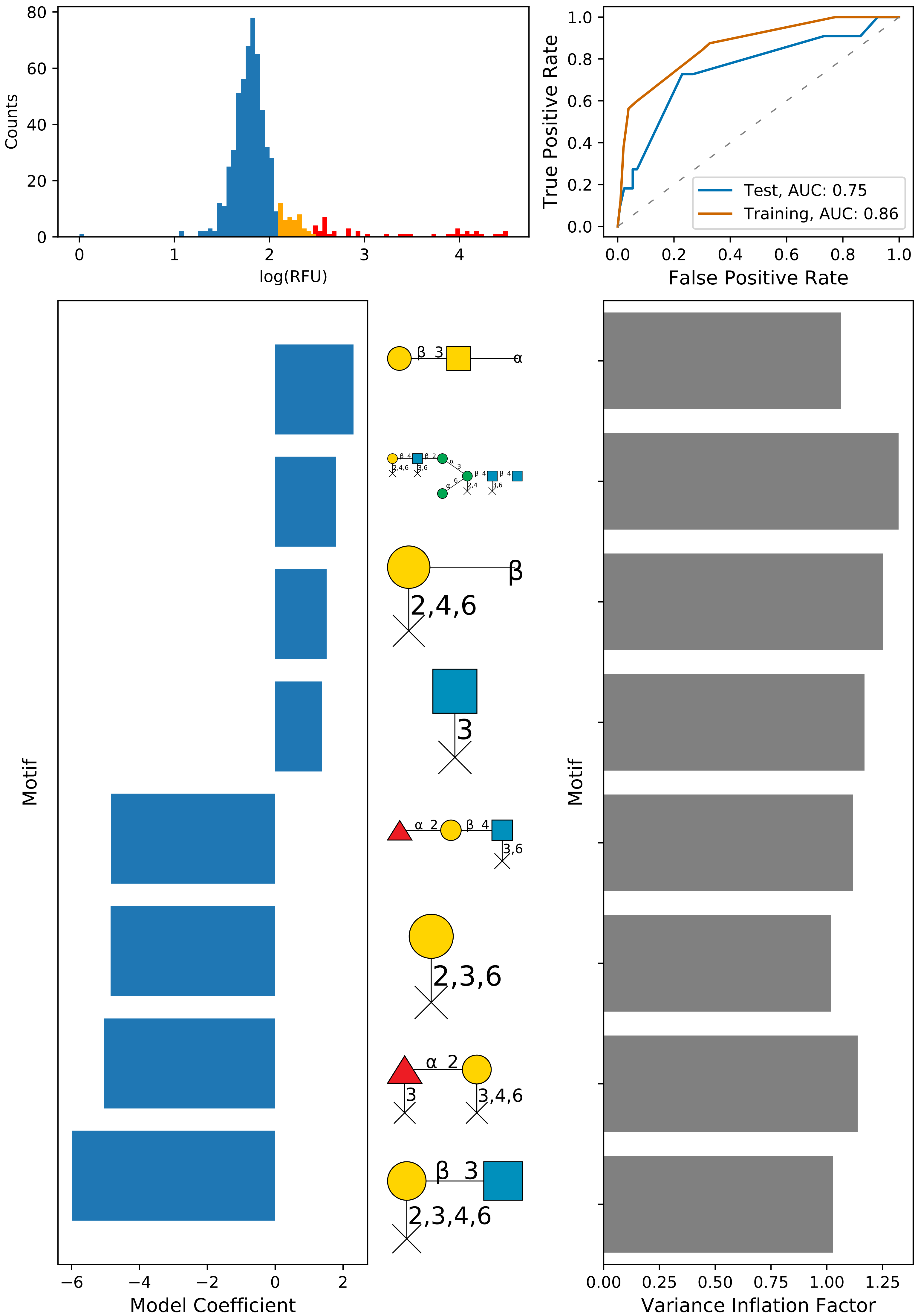

GSL-I-B4_10ug_13920_v5.0_DATA.csv

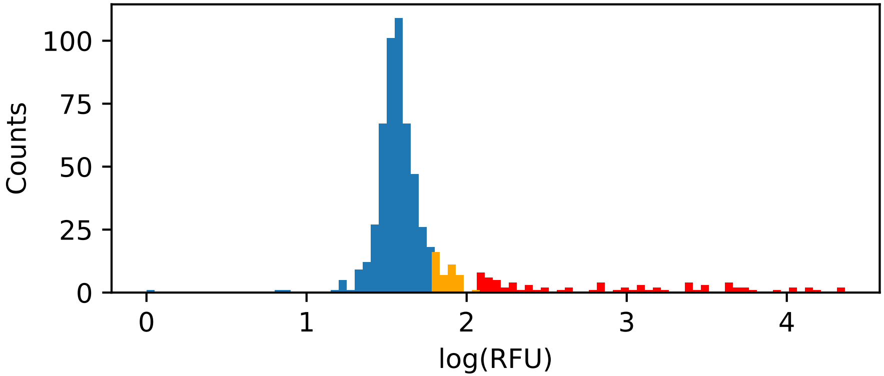

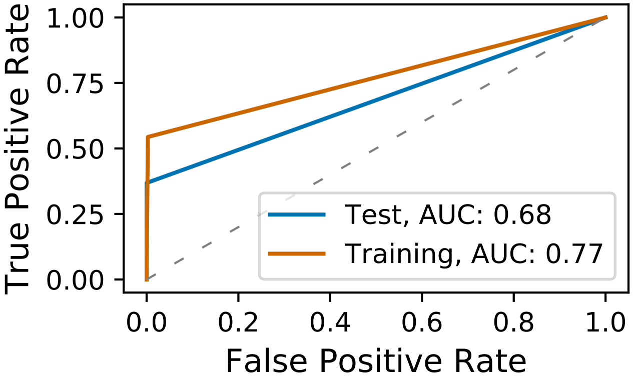

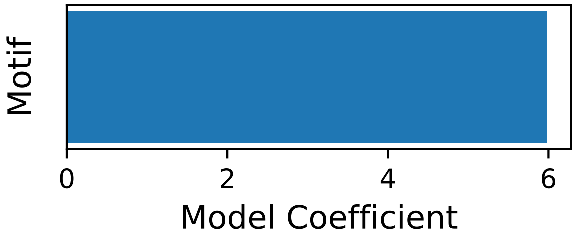

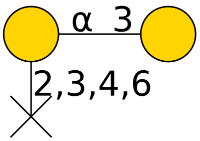

jacalin-1ug_14301_v5.0_DATA.csv

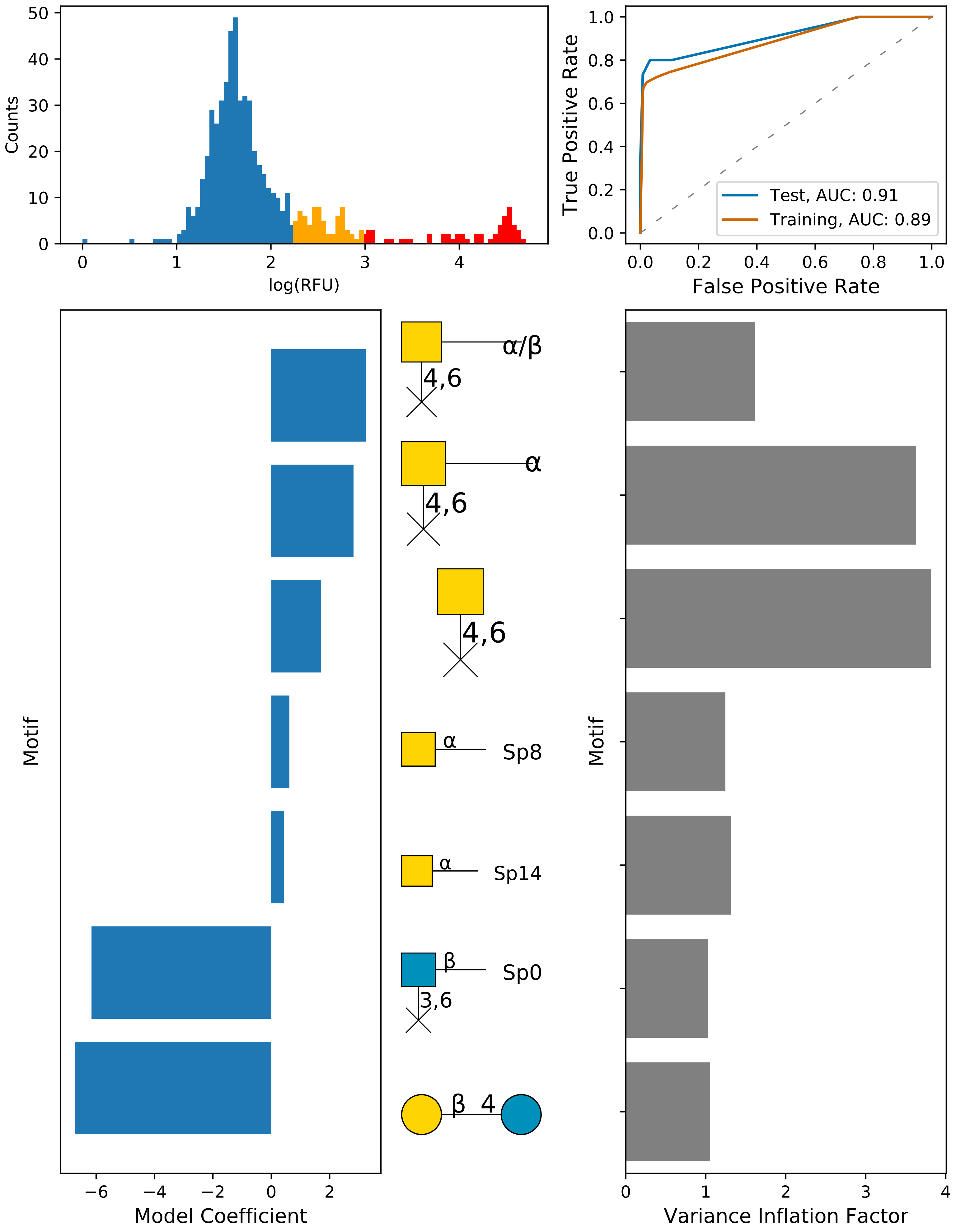

WGA_14057_1ug_v5.0_DATA.csv

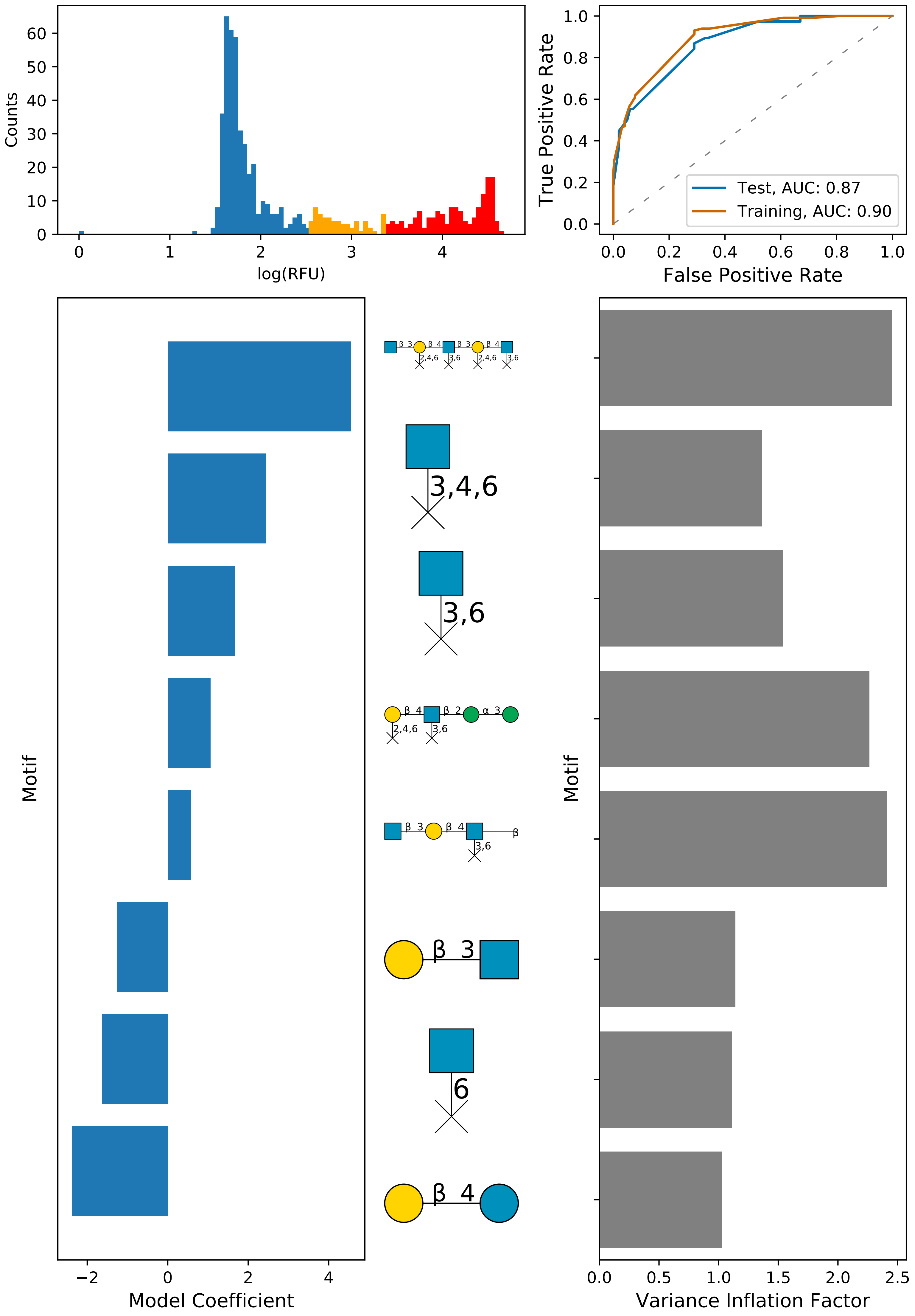

UEAI_100ug_13806_v5.0_DATA.csv

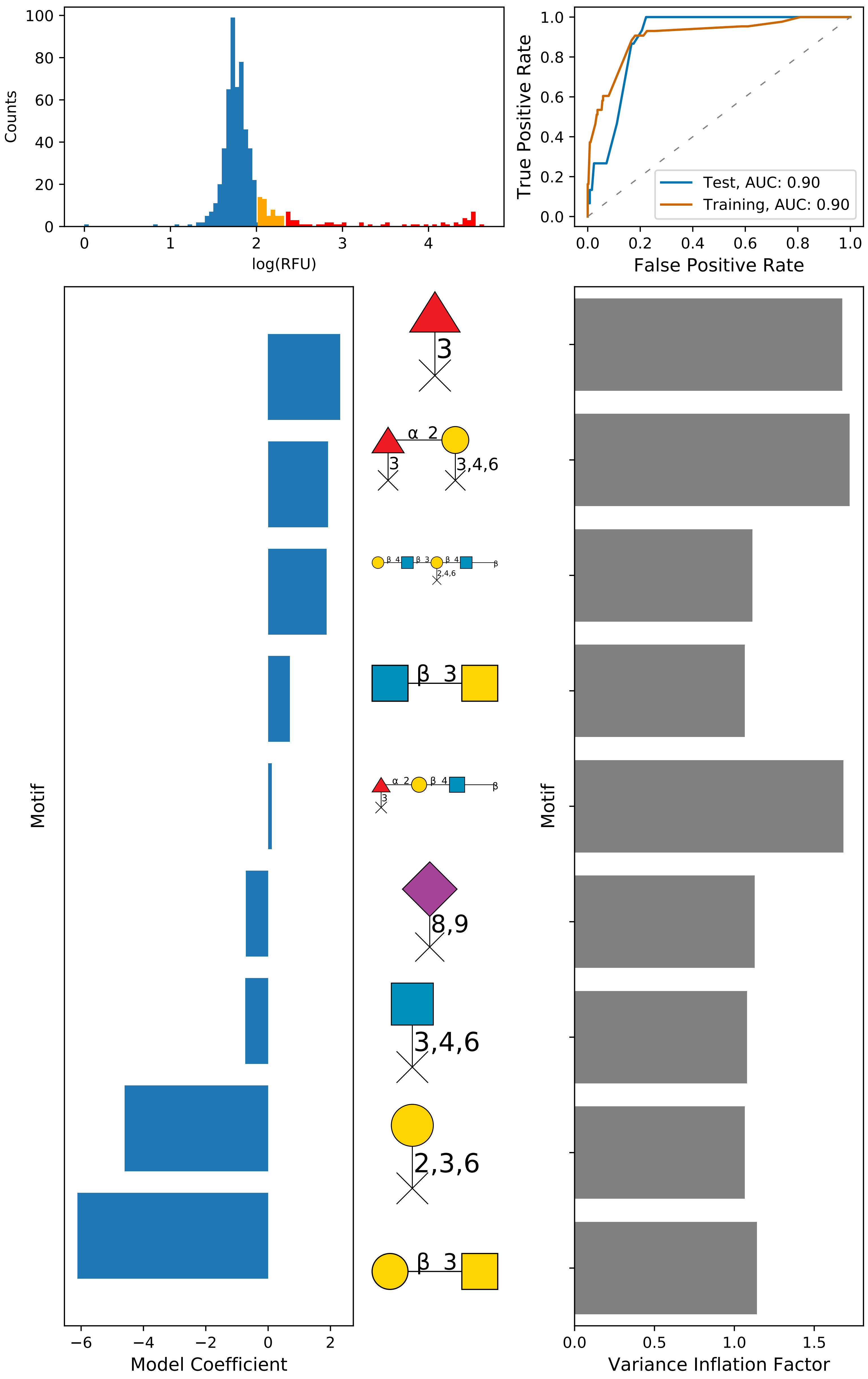

SBA_14042_10ug_v5.0_DATA.csv

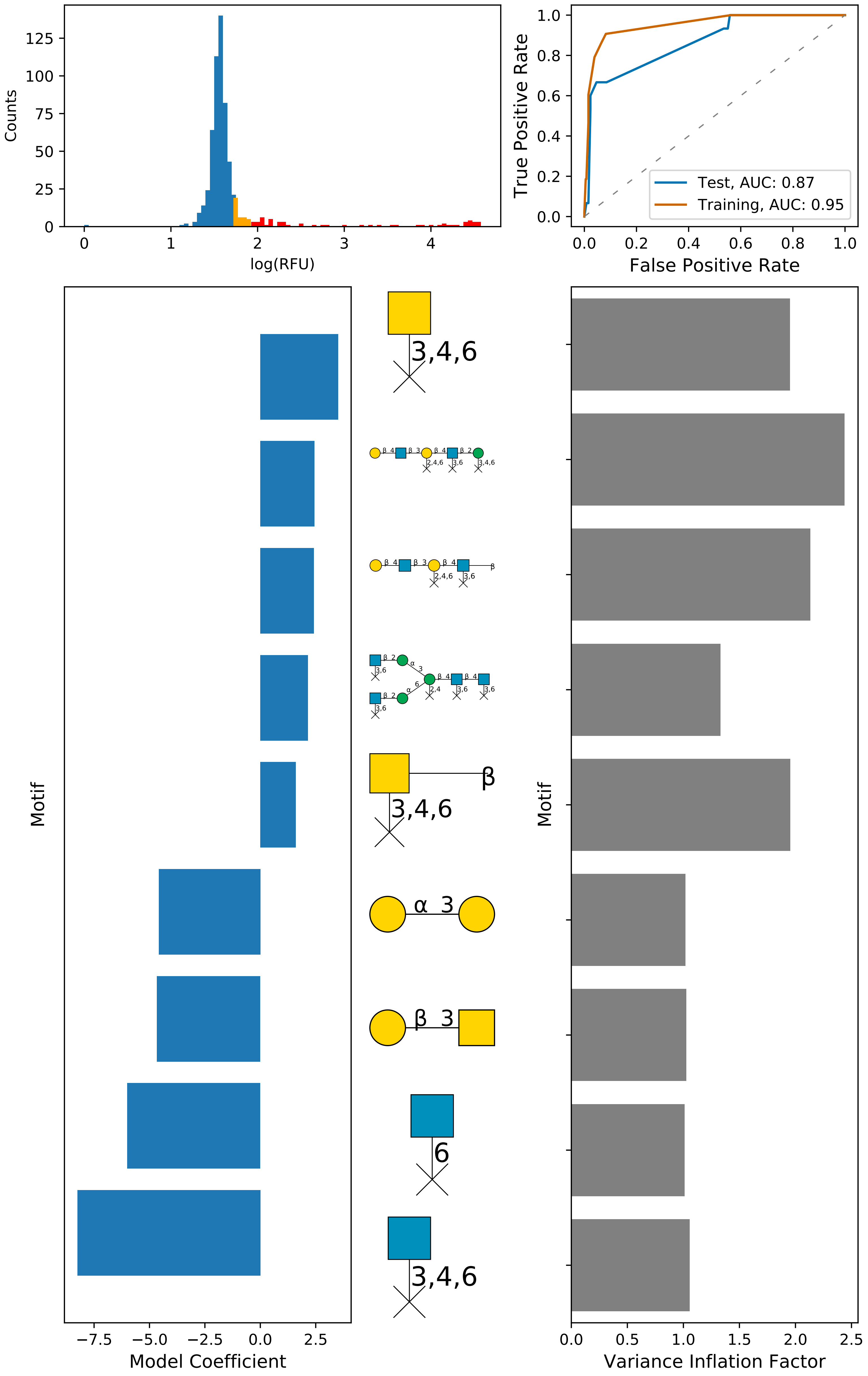

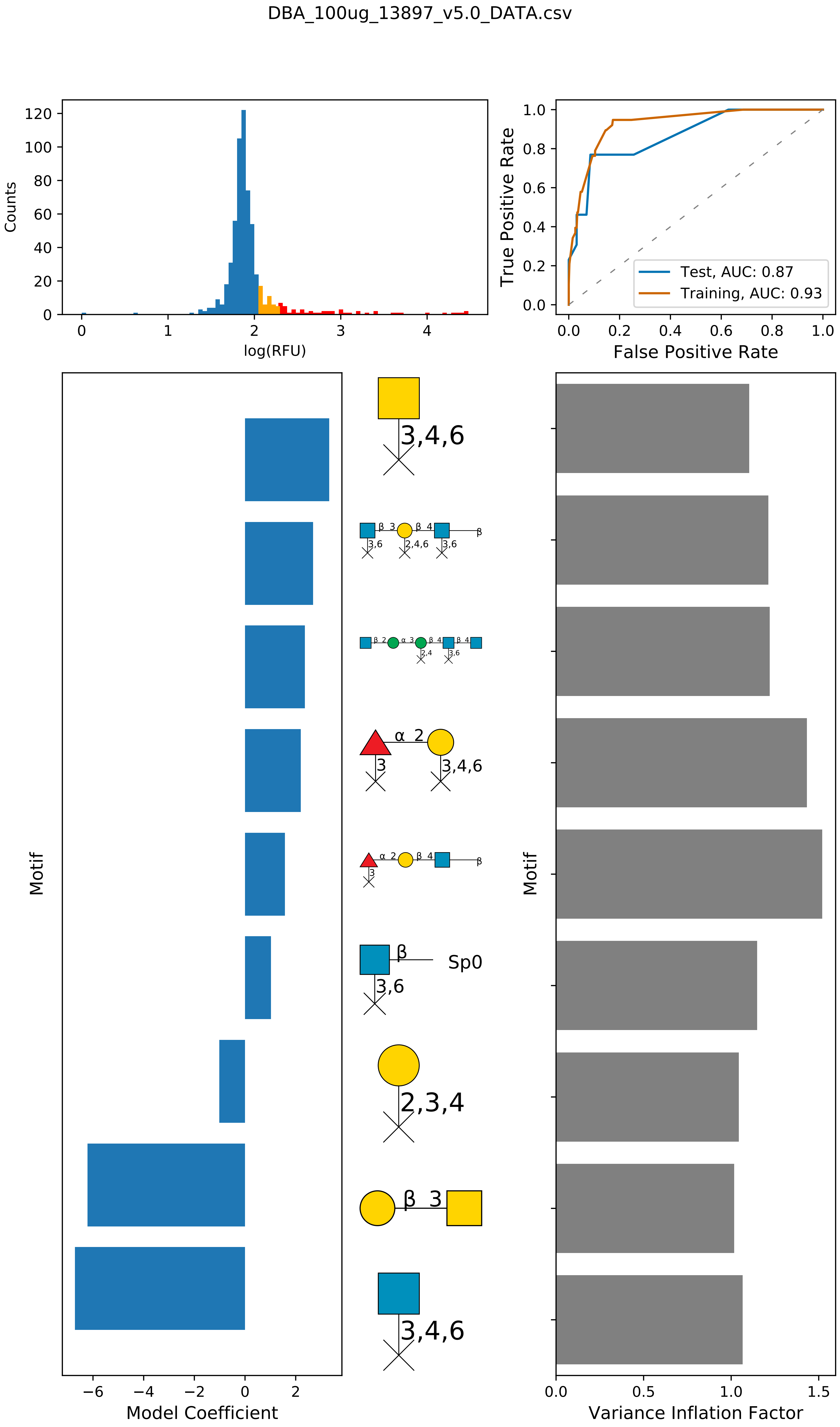

DBA_100ug_13897_v5.0_DATA.csv
Counts
log(RFU)
True Positive Rate
False Positive Rate
Test, AUC: 0.87
Training, AUC: 0.93
Motif
Model Coefficient
Variance Inflation Factor
3,4,6
Sp0
2,3,4

PSA_14040_10ug_v5.0_DATA.csv

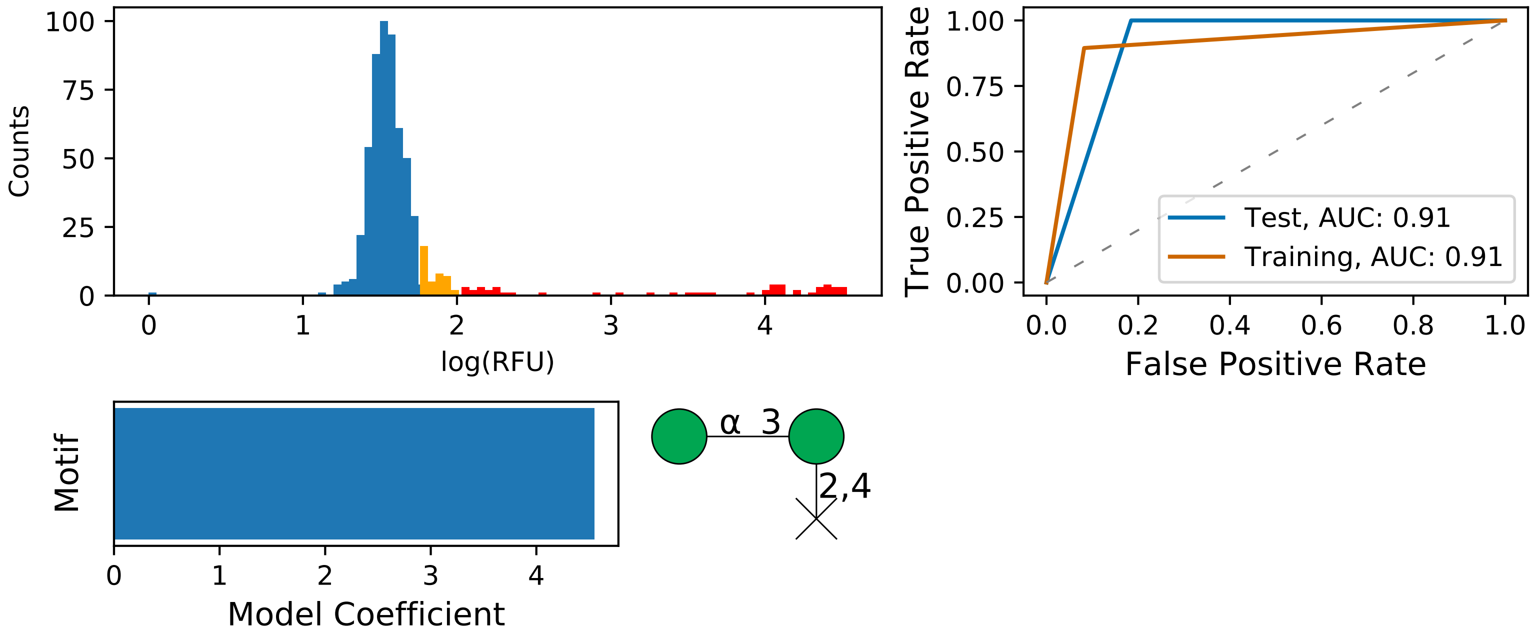

HA_PuertoRico_8_34_13829_v5_DATA.csv

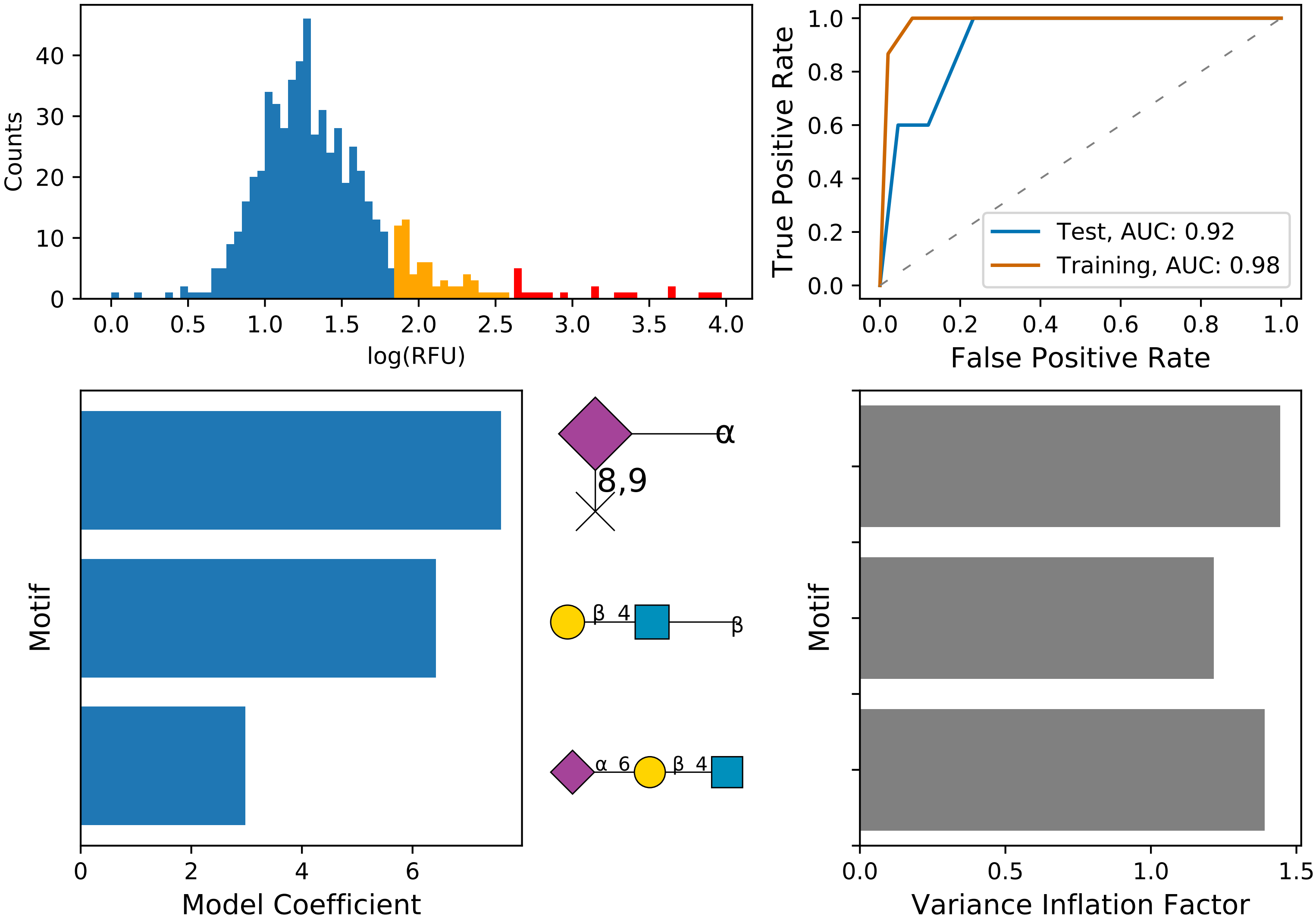

H3N8-HA_16686_v5.1_DATA.csv

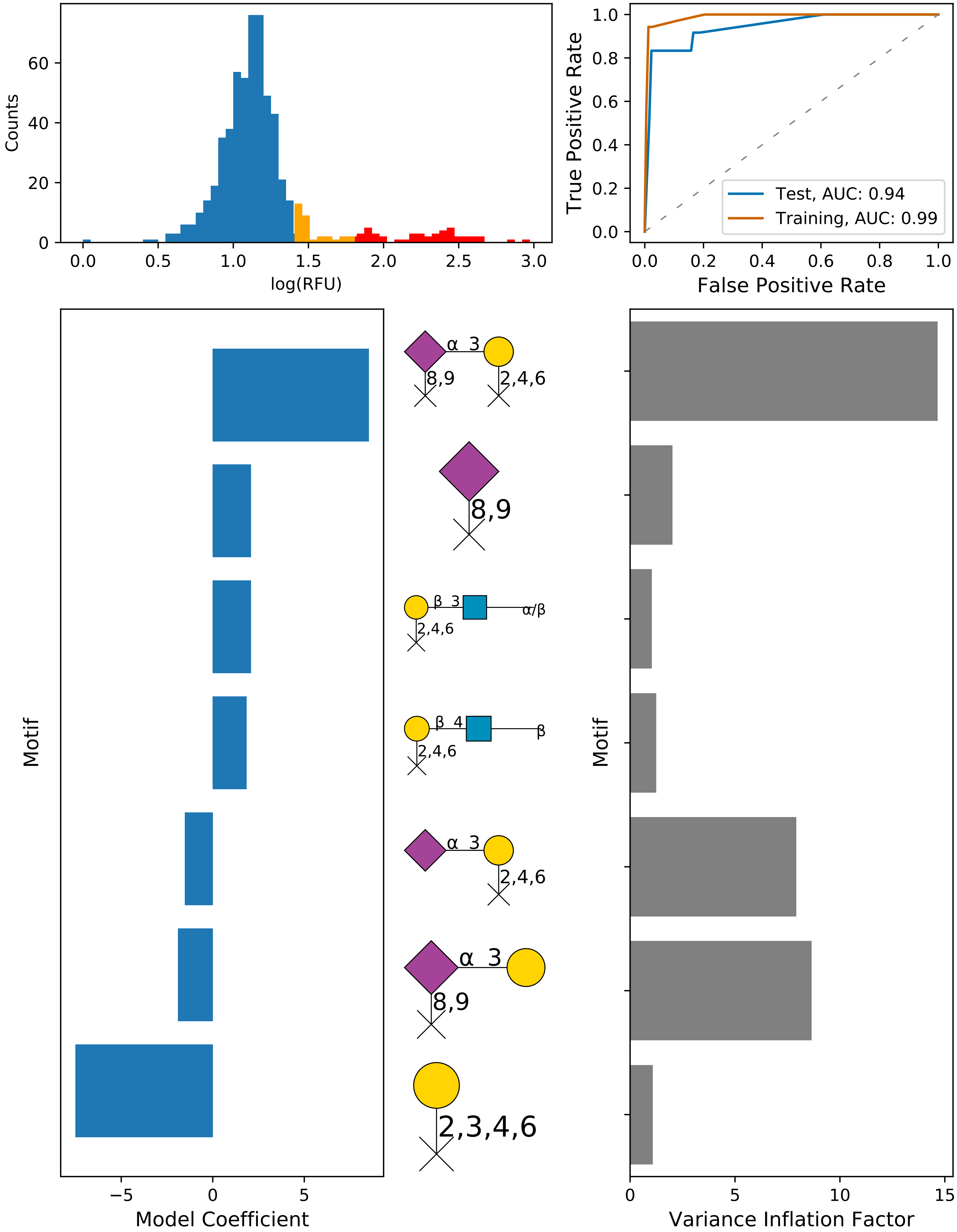

Human-DC-Sign-tetramer_15320_v5.0_DATA.csv

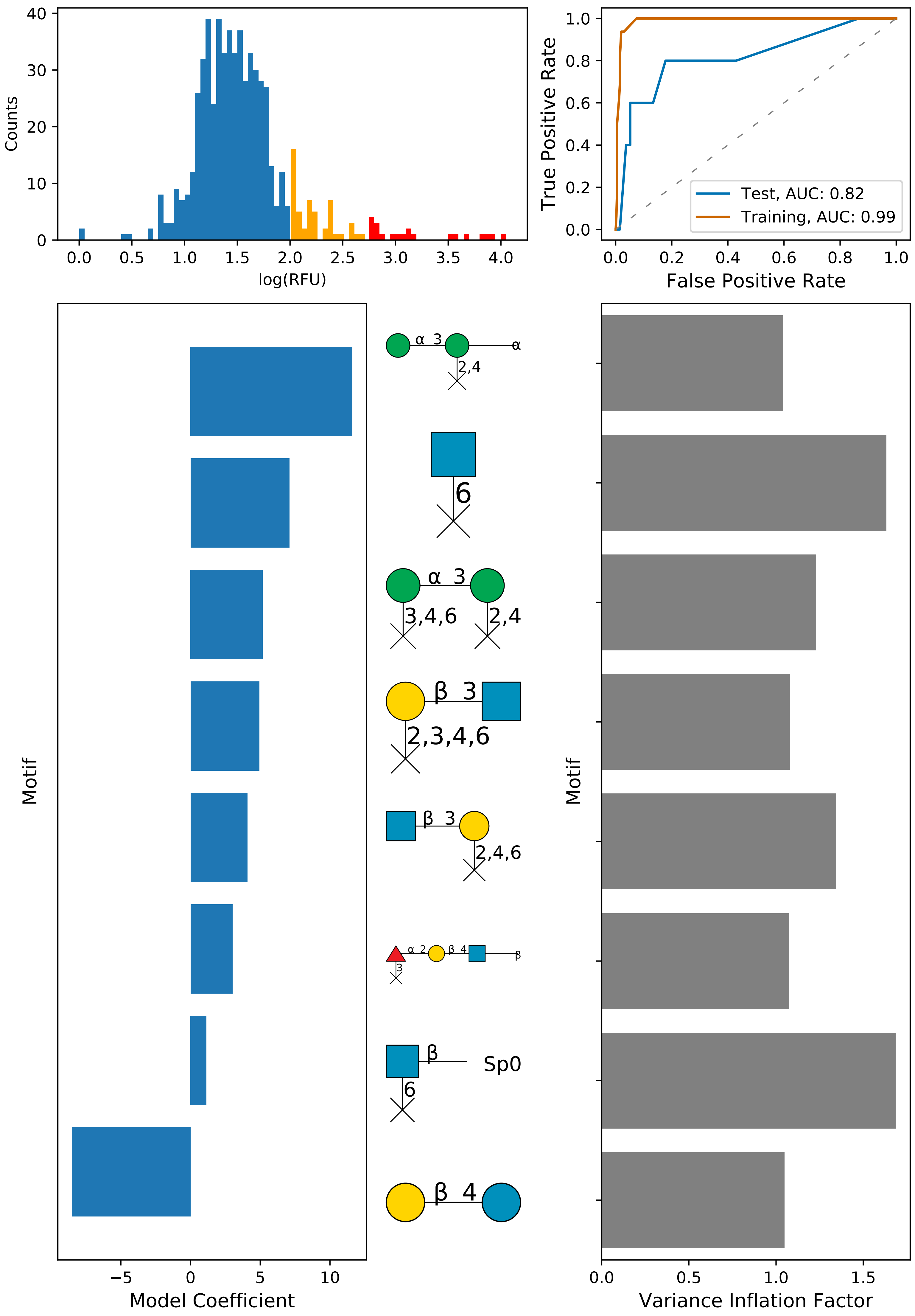

Supplement: Supplementary file 3 — Additional file 3 Motif identification for a range of glycan microarrays. Detailed motifs and classifier performance for the range of glycan microarrays presented in Table 1. [file 12859_2020_3374_MOESM3_ESM.pdf]
