# Supplementary material for: Identifying glycan motifs using a novel subtree mining approach
Source: BMC Bioinformatics. 2020 Feb 4;21:42. doi: 10.1186/s12859-020-3374-4 (PMC7001330; doi:10.1186/s12859-020-3374-4)
Supplement: Supplementary file 4 — Additional file 4 Comparison of MAD-based detection of positive binders to other methods for detecting positive binding glycans. Detection of positive binding glycans by median absolute deviation (MAD) compared to the z-score threshold employed by Cholleti et al. [14] and the ‘Universal Thresholding’ approach employed by Wang et al. [25]. For the z-score threshold, z-scores were calculated for the average RFU values across all concentrations (as outlined by Cholleti et al. [14]). For the Universal Threshold approach, only microarrays within a determined linear range (see Wang et al. [25] for further details) were used to determine positive binders. As the MAD-based thresholding approach is applied to each microarray separately, individual plots for each concentration are shown for MAD-based thresholding. [file 12859_2020_3374_MOESM4_ESM.pdf]

ABA

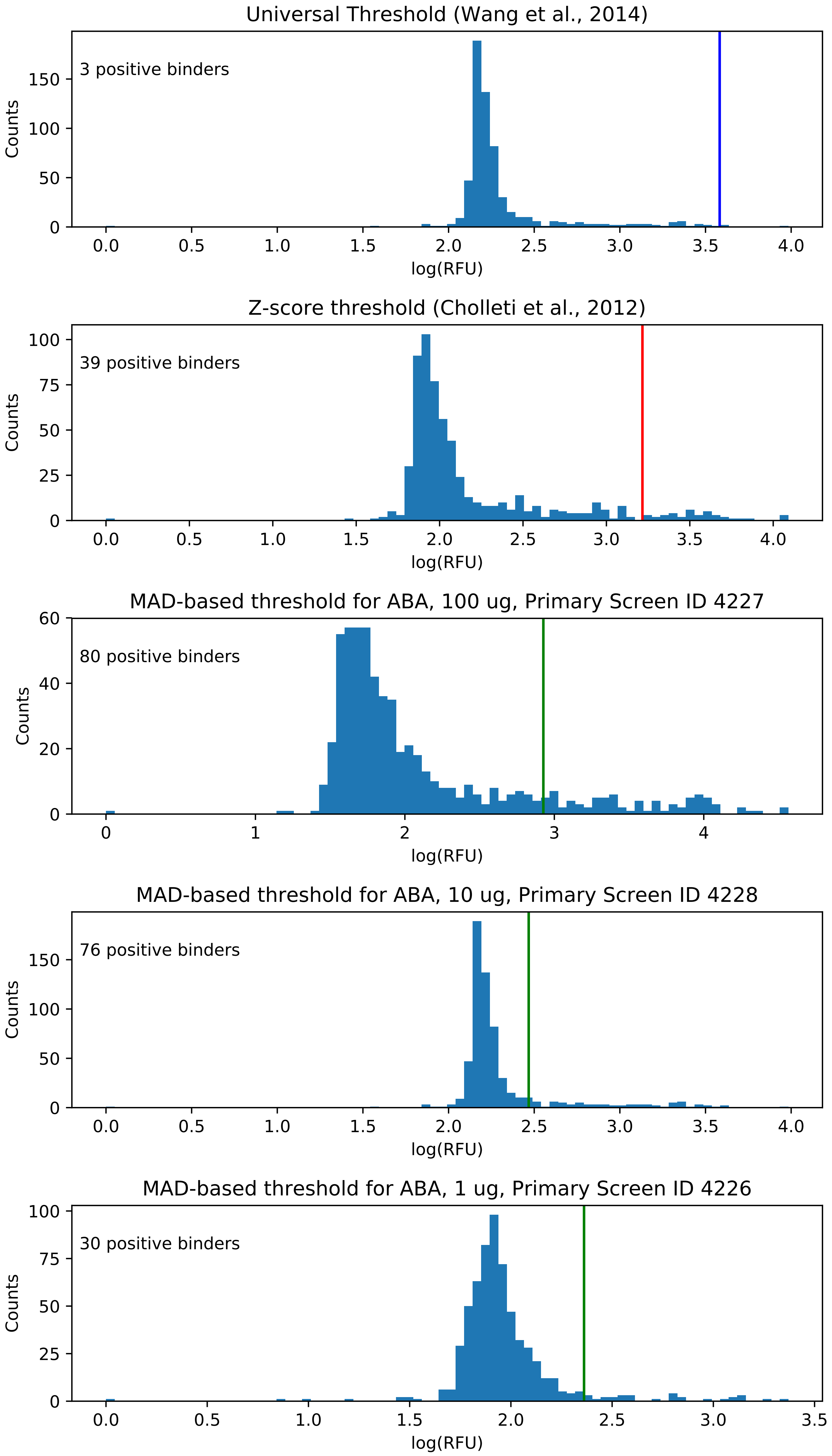
Universal Threshold (Wang et al., 2014)
3 positive binders
Counts
log(RFU)
Z-score threshold (Cholleti et al., 2012)
39 positive binders
Counts
log(RFU)
MAD-based threshold for ABA, 100 ug, Primary Screen ID 4227
80 positive binders
Counts
log(RFU)
MAD-based threshold for ABA, 10 ug, Primary Screen ID 4228
76 positive binders
Counts
log(RFU)
MAD-based threshold for ABA, 1 ug, Primary Screen ID 4226
30 positive binders
Counts
log(RFU)

Con A

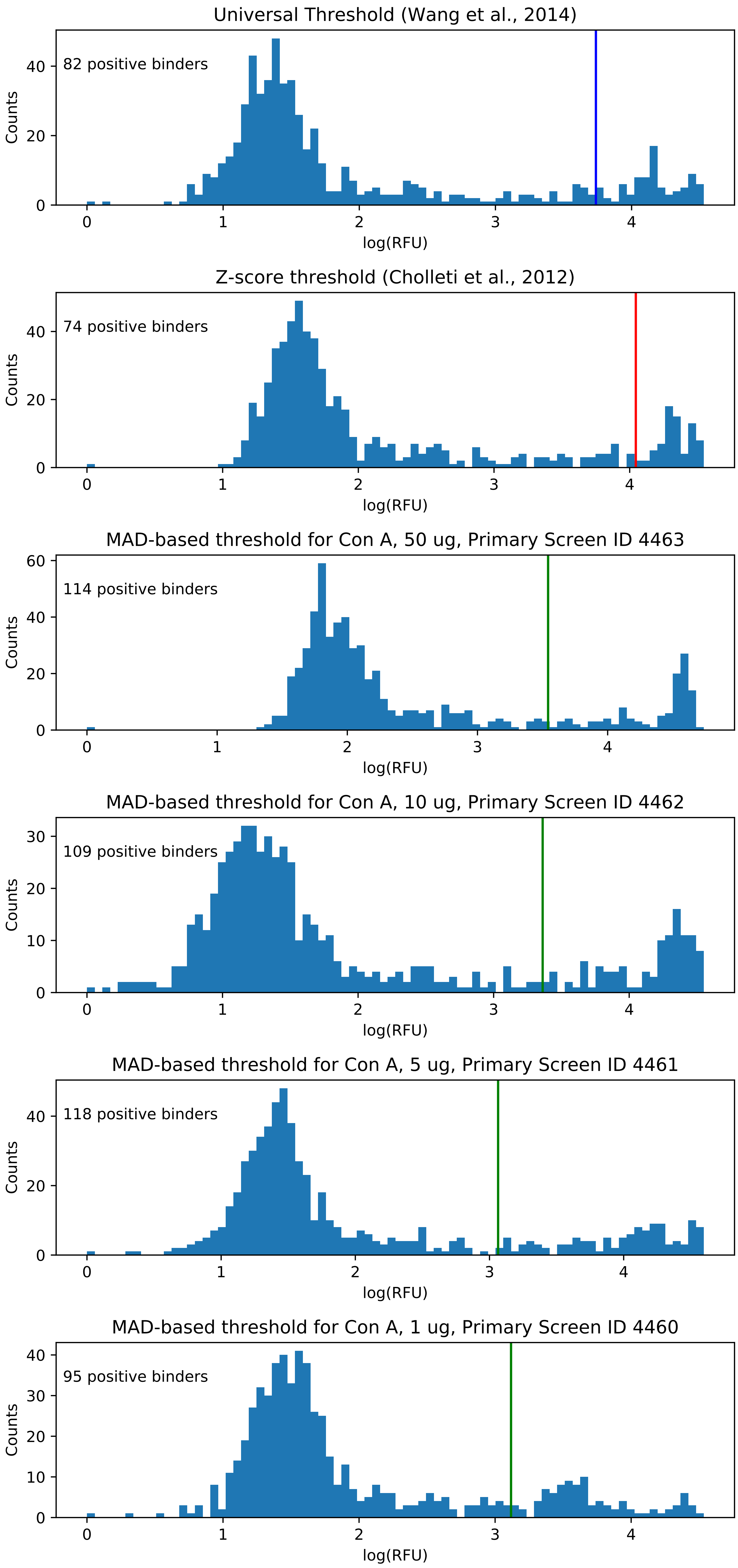
Universal Threshold (Wang et al., 2014)
82 positive binders
Counts
log(RFU)
Z-score threshold (Cholleti et al., 2012)
74 positive binders
Counts
log(RFU)
MAD-based threshold for Con A, 50 ug, Primary Screen ID 4463
114 positive binders
Counts
log(RFU)
MAD-based threshold for Con A, 10 ug, Primary Screen ID 4462
109 positive binders
Counts
log(RFU)
MAD-based threshold for Con A, 5 ug, Primary Screen ID 4461
118 positive binders
Counts
log(RFU)
MAD-based threshold for Con A, 1 ug, Primary Screen ID 4460
95 positive binders
Counts
log(RFU)

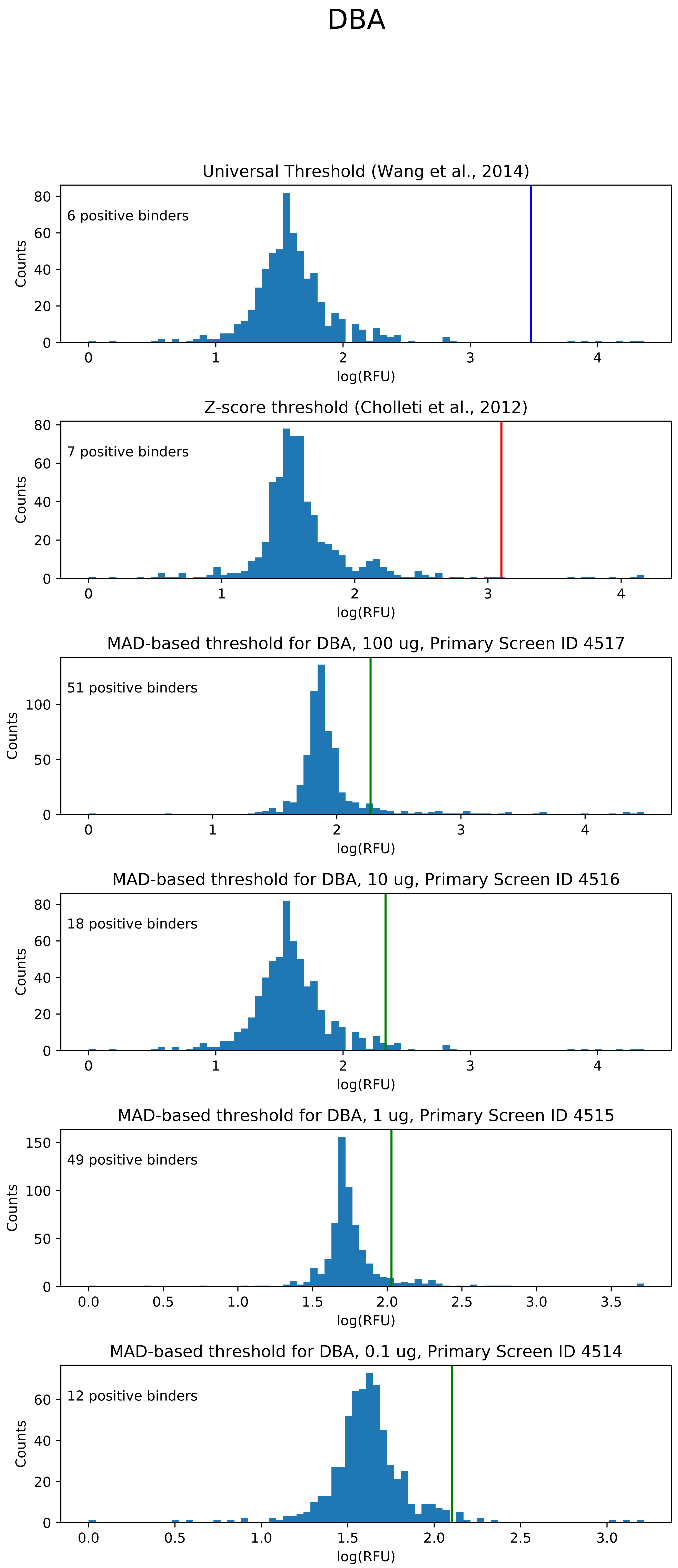
DBA
Universal Threshold (Wang et al., 2014)
6 positive binders
Counts
log(RFU)
Z-score threshold (Cholleti et al., 2012)
7 positive binders
Counts
log(RFU)
MAD-based threshold for DBA, 100 ug, Primary Screen ID 4517
51 positive binders
Counts
log(RFU)
MAD-based threshold for DBA, 10 ug, Primary Screen ID 4516
18 positive binders
Counts
log(RFU)
MAD-based threshold for DBA, 1 ug, Primary Screen ID 4515
49 positive binders
Counts
log(RFU)
MAD-based threshold for DBA, 0.1 ug, Primary Screen ID 4514
12 positive binders
Counts
log(RFU)

GSL I B4

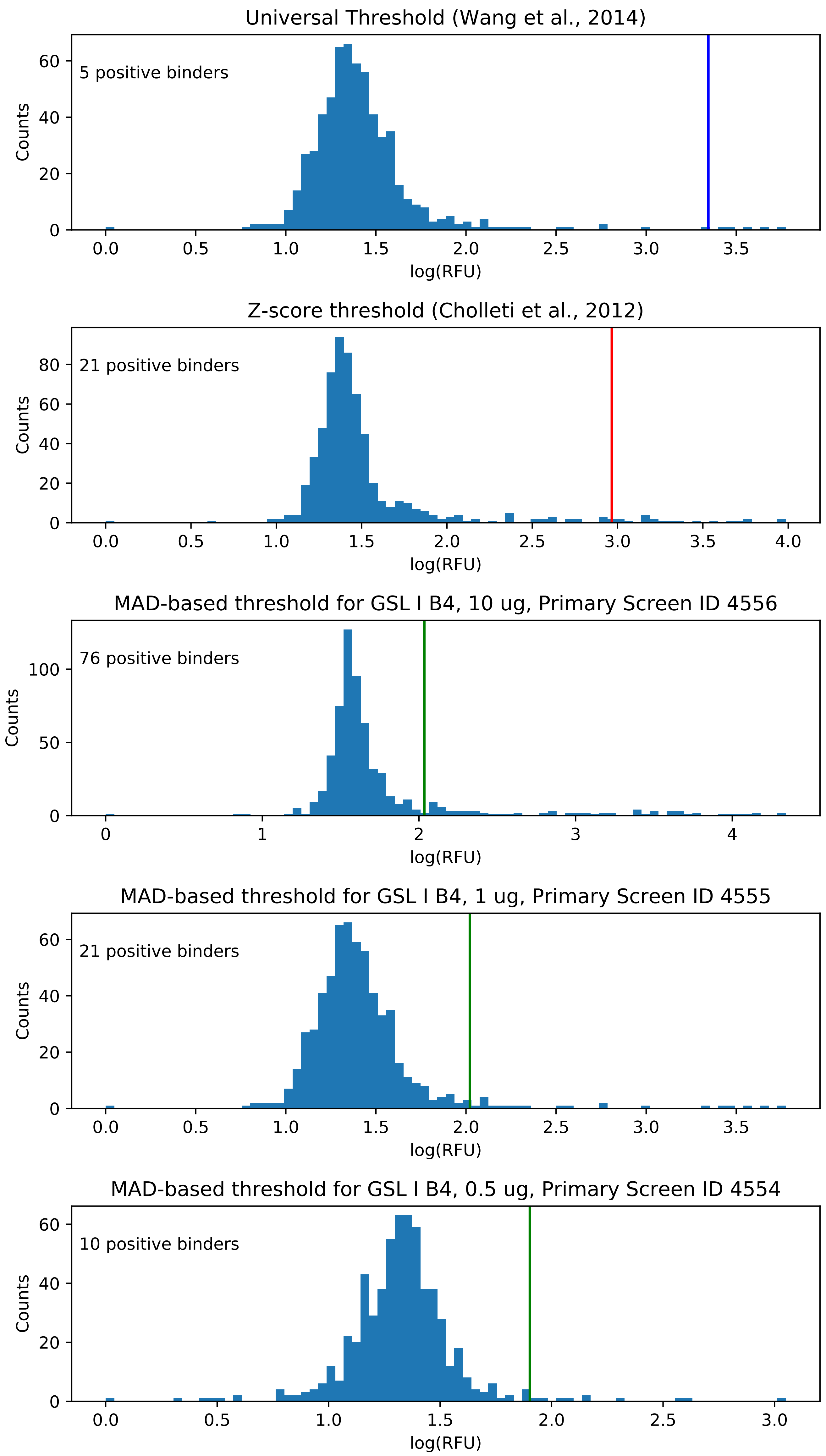
Universal Threshold (Wang et al., 2014)
5 positive binders
Counts
log(RFU)
Z-score threshold (Cholleti et al., 2012)
21 positive binders
Counts
log(RFU)
MAD-based threshold for GSL I B4, 10 ug, Primary Screen ID 4556
76 positive binders
Counts
log(RFU)
MAD-based threshold for GSL I B4, 1 ug, Primary Screen ID 4555
21 positive binders
Counts
log(RFU)
MAD-based threshold for GSL I B4, 0.5 ug, Primary Screen ID 4554
10 positive binders
Counts
log(RFU)

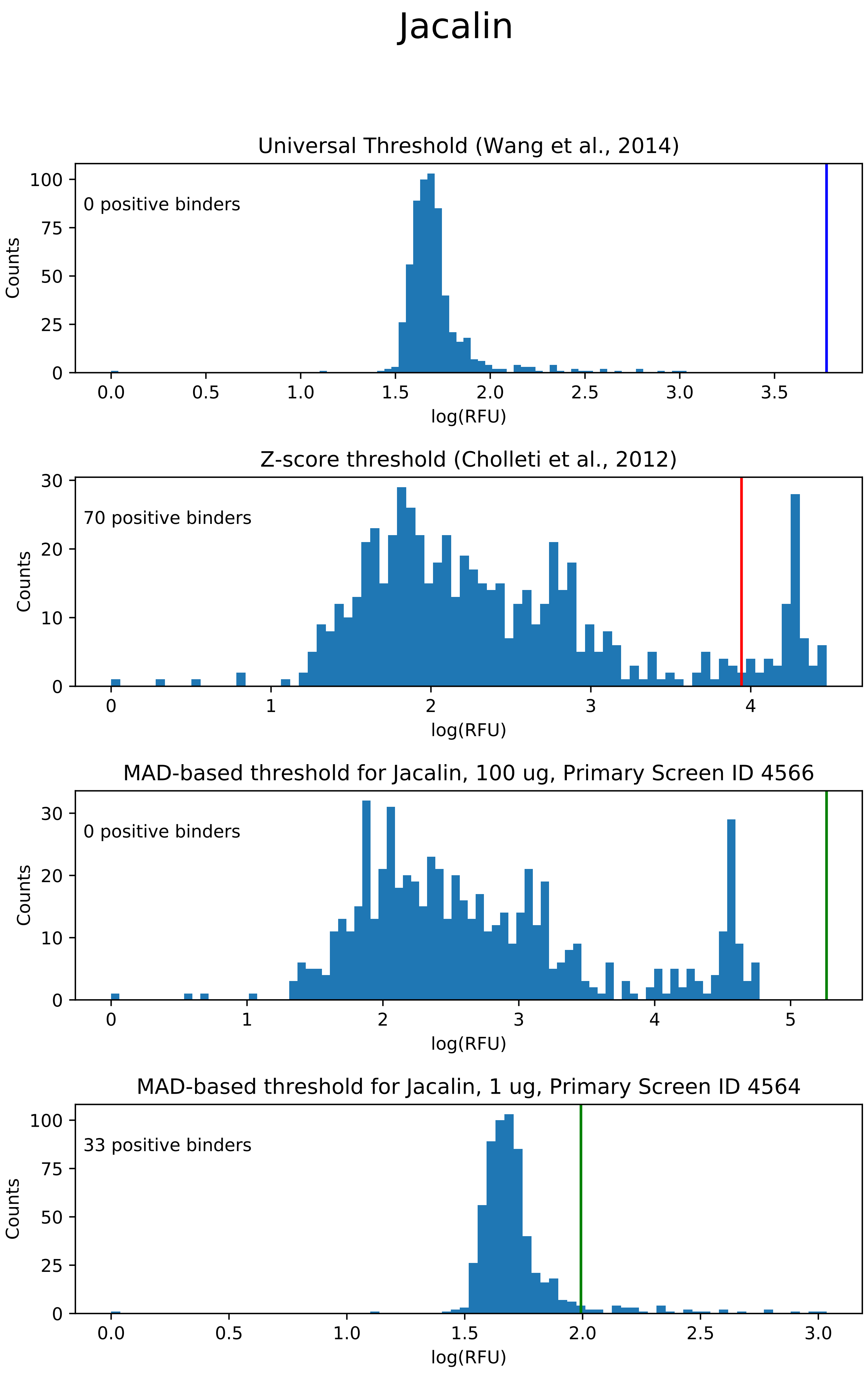

Jacalin
Universal Threshold (Wang et al., 2014)
0 positive binders
Counts
log(RFU)
0.0
0.5
1.0
1.5
2.0
2.5
3.0
3.5
0
25
50
75
100
Z-score threshold (Cholleti et al., 2012)
70 positive binders
Counts
log(RFU)
0
1
2
3
4
0
10
20
30
MAD-based threshold for Jacalin, 100 ug, Primary Screen ID 4566
0 positive binders
Counts
log(RFU)
0
1
2
3
4
5
0
10
20
30
MAD-based threshold for Jacalin, 1 ug, Primary Screen ID 4564
33 positive binders
Counts
log(RFU)
0.0
0.5
1.0
1.5
2.0
2.5
3.0
0
25
50
75
100

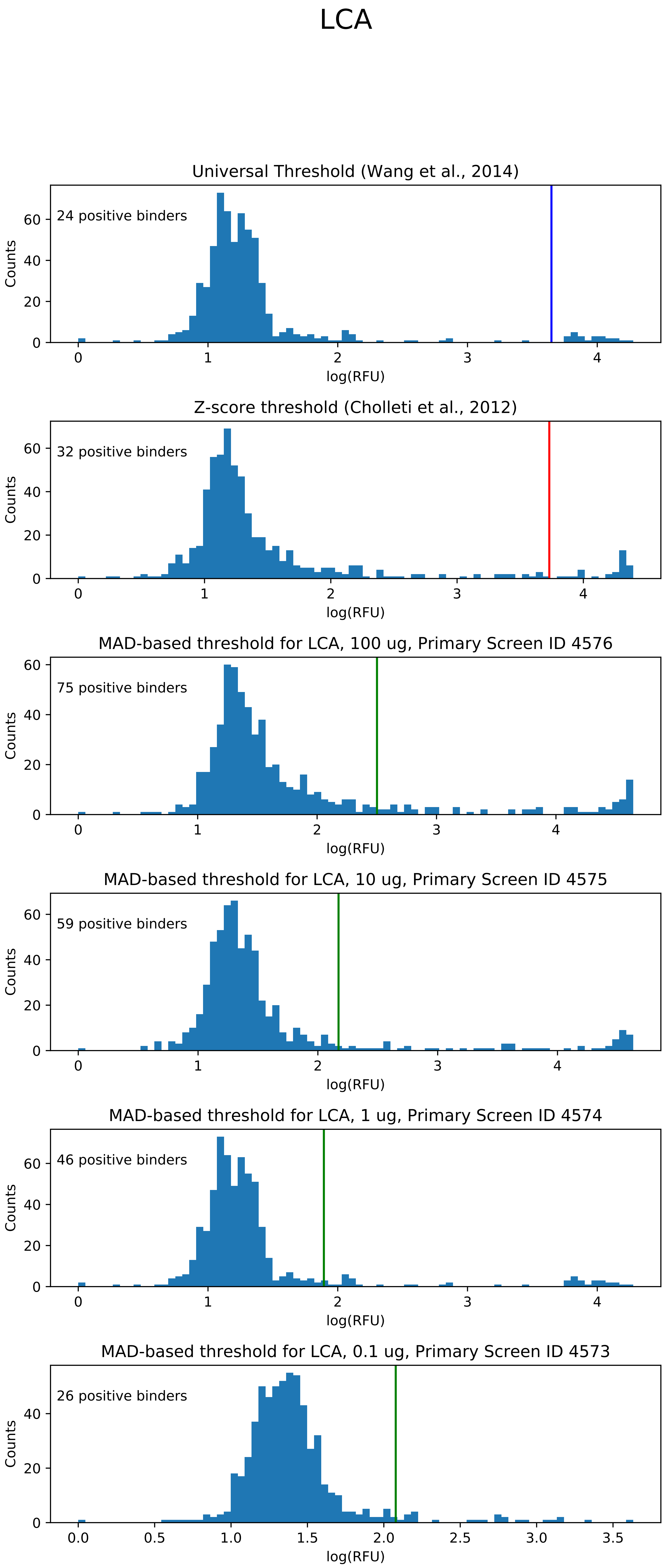
LCA
Universal Threshold (Wang et al., 2014)
24 positive binders
Z-score threshold (Cholleti et al., 2012)
32 positive binders
MAD-based threshold for LCA, 100 ug, Primary Screen ID 4576
75 positive binders
MAD-based threshold for LCA, 10 ug, Primary Screen ID 4575
59 positive binders
MAD-based threshold for LCA, 1 ug, Primary Screen ID 4574
46 positive binders
MAD-based threshold for LCA, 0.1 ug, Primary Screen ID 4573
26 positive binders
Counts
log(RFU)

MAL I

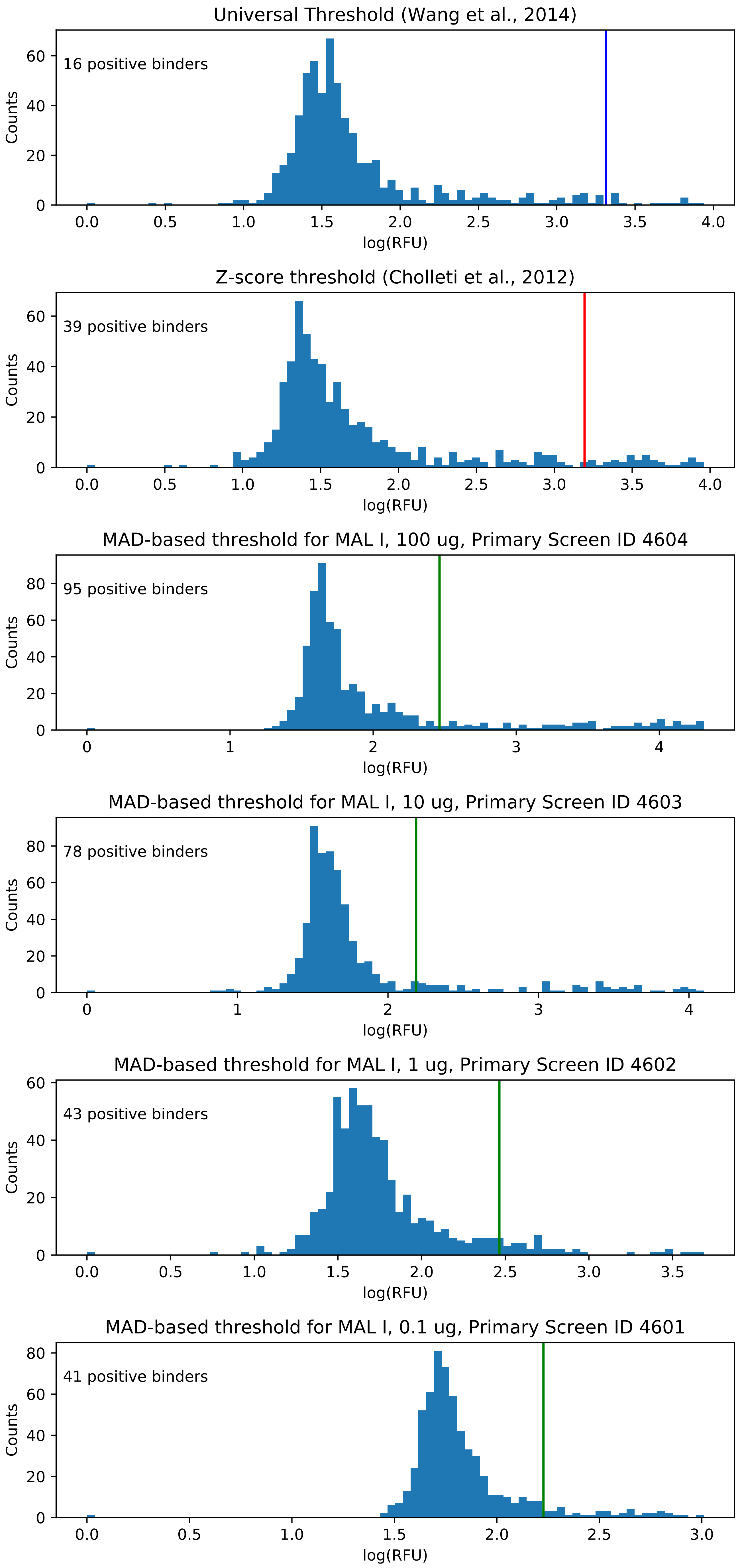

Universal Threshold (Wang et al., 2014)
16 positive binders
Z-score threshold (Cholleti et al., 2012)
39 positive binders
MAD-based threshold for MAL I, 100 ug, Primary Screen ID 4604
95 positive binders
MAD-based threshold for MAL I, 10 ug, Primary Screen ID 4603
78 positive binders
MAD-based threshold for MAL I, 1 ug, Primary Screen ID 4602
43 positive binders
MAD-based threshold for MAL I, 0.1 ug, Primary Screen ID 4601
41 positive binders
Counts
log(RFU)

MAL II

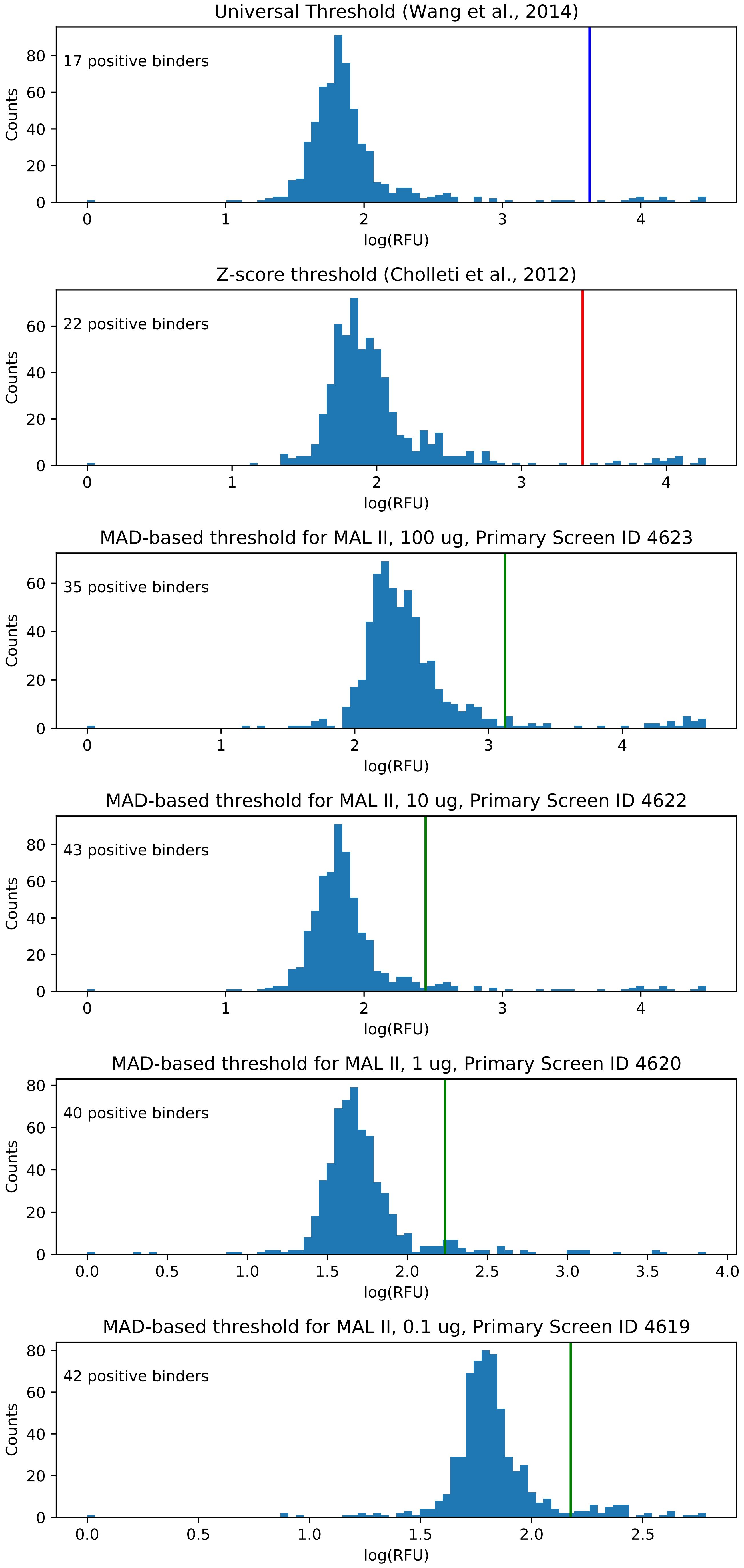
Universal Threshold (Wang et al., 2014)
17 positive binders
Z-score threshold (Cholleti et al., 2012)
22 positive binders
MAD-based threshold for MAL II, 100 ug, Primary Screen ID 4623
35 positive binders
MAD-based threshold for MAL II, 10 ug, Primary Screen ID 4622
43 positive binders
MAD-based threshold for MAL II, 1 ug, Primary Screen ID 4620
40 positive binders
MAD-based threshold for MAL II, 0.1 ug, Primary Screen ID 4619
42 positive binders
Counts
log(RFU)

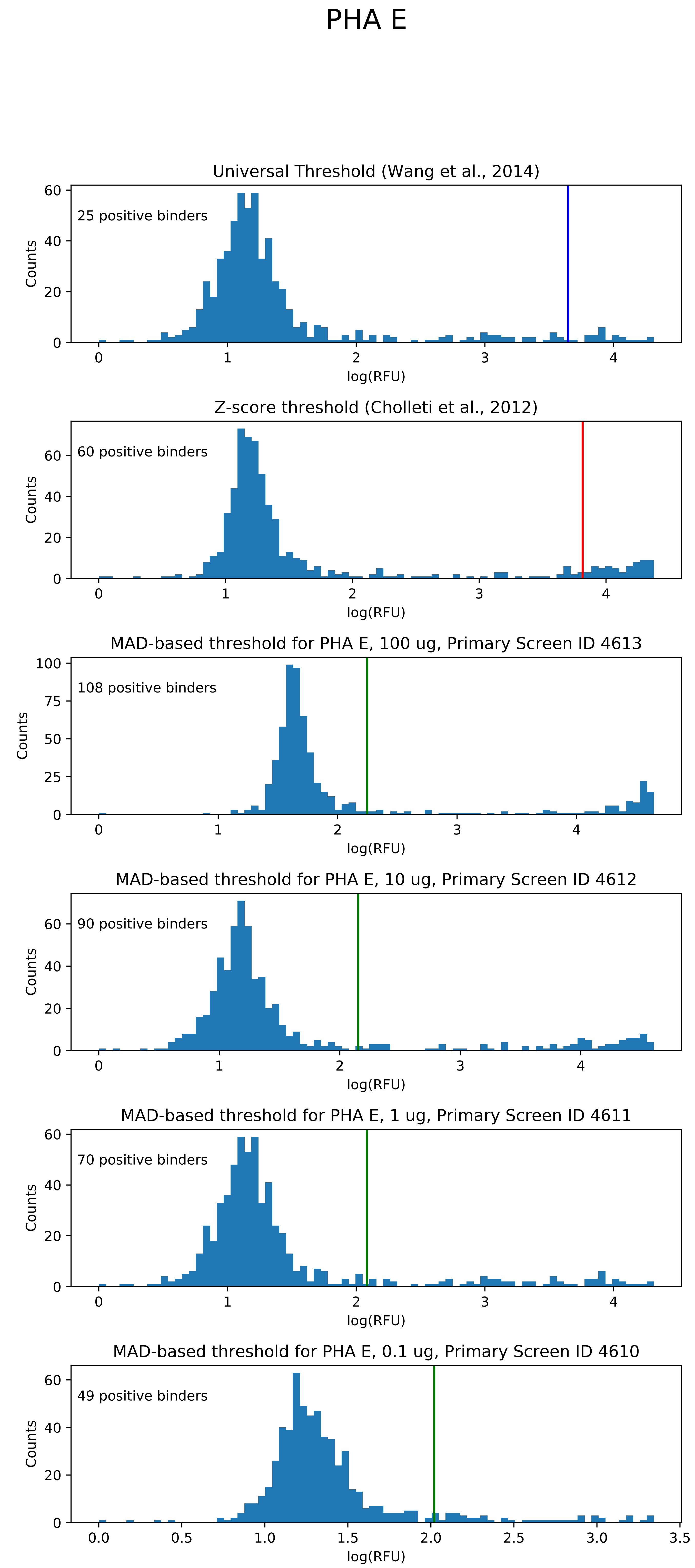
PHA E
Universal Threshold (Wang et al., 2014)
25 positive binders
Z-score threshold (Cholleti et al., 2012)
60 positive binders
MAD-based threshold for PHA E, 100 ug, Primary Screen ID 4613
108 positive binders
MAD-based threshold for PHA E, 10 ug, Primary Screen ID 4612
90 positive binders
MAD-based threshold for PHA E, 1 ug, Primary Screen ID 4611
70 positive binders
MAD-based threshold for PHA E, 0.1 ug, Primary Screen ID 4610
49 positive binders
Counts
log(RFU)

PHA L

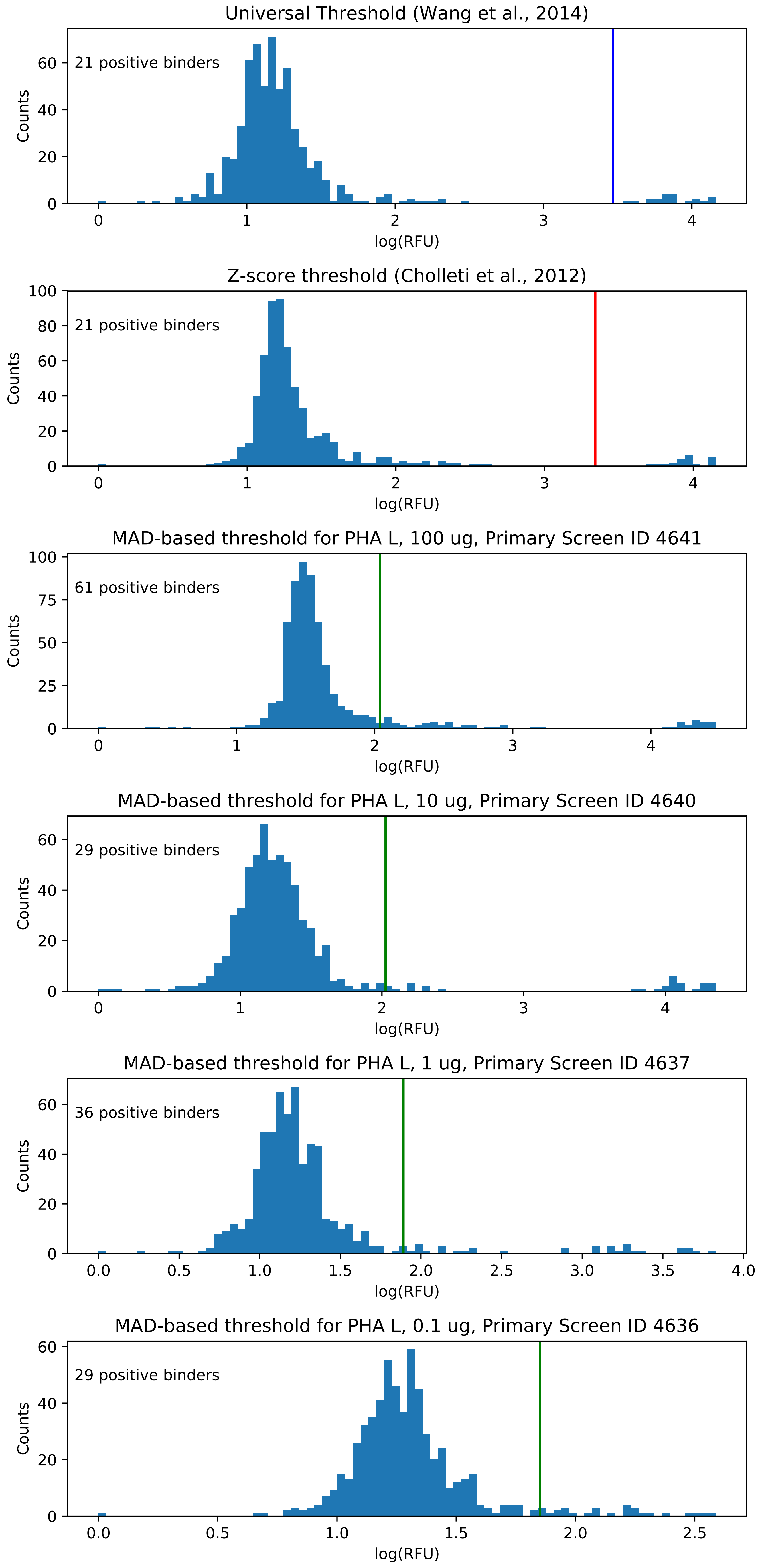
Universal Threshold (Wang et al., 2014)
21 positive binders
Counts
log(RFU)
Z-score threshold (Cholleti et al., 2012)
21 positive binders
Counts
log(RFU)
MAD-based threshold for PHA L, 100 ug, Primary Screen ID 4641
61 positive binders
Counts
log(RFU)
MAD-based threshold for PHA L, 10 ug, Primary Screen ID 4640
29 positive binders
Counts
log(RFU)
MAD-based threshold for PHA L, 1 ug, Primary Screen ID 4637
36 positive binders
Counts
log(RFU)
MAD-based threshold for PHA L, 0.1 ug, Primary Screen ID 4636
29 positive binders
Counts
log(RFU)

PNA

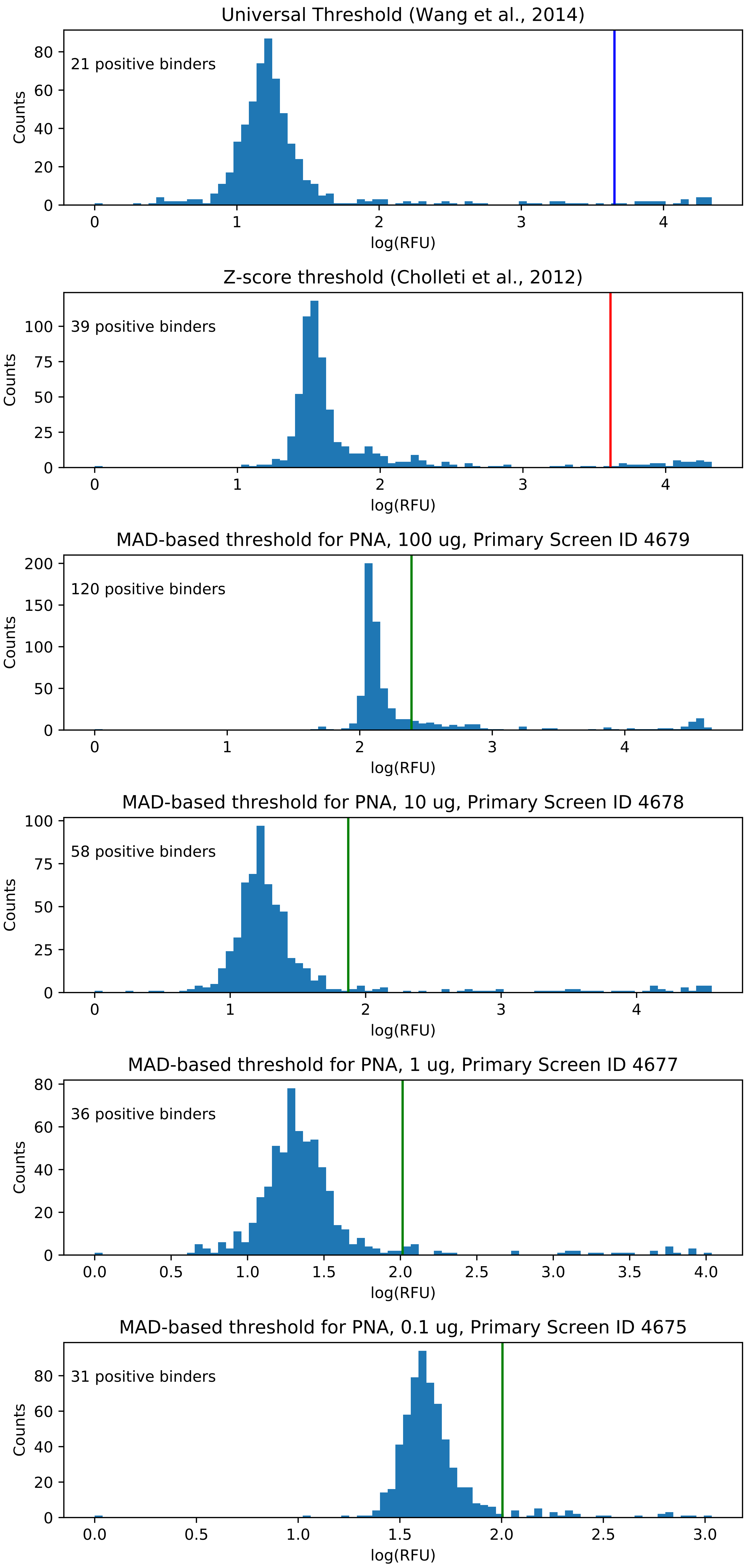
Universal Threshold (Wang et al., 2014)
21 positive binders
Counts
log(RFU)
Z-score threshold (Cholleti et al., 2012)
39 positive binders
Counts
log(RFU)
MAD-based threshold for PNA, 100 ug, Primary Screen ID 4679
120 positive binders
Counts
log(RFU)
MAD-based threshold for PNA, 10 ug, Primary Screen ID 4678
58 positive binders
Counts
log(RFU)
MAD-based threshold for PNA, 1 ug, Primary Screen ID 4677
36 positive binders
Counts
log(RFU)
MAD-based threshold for PNA, 0.1 ug, Primary Screen ID 4675
31 positive binders
Counts
log(RFU)

PSA

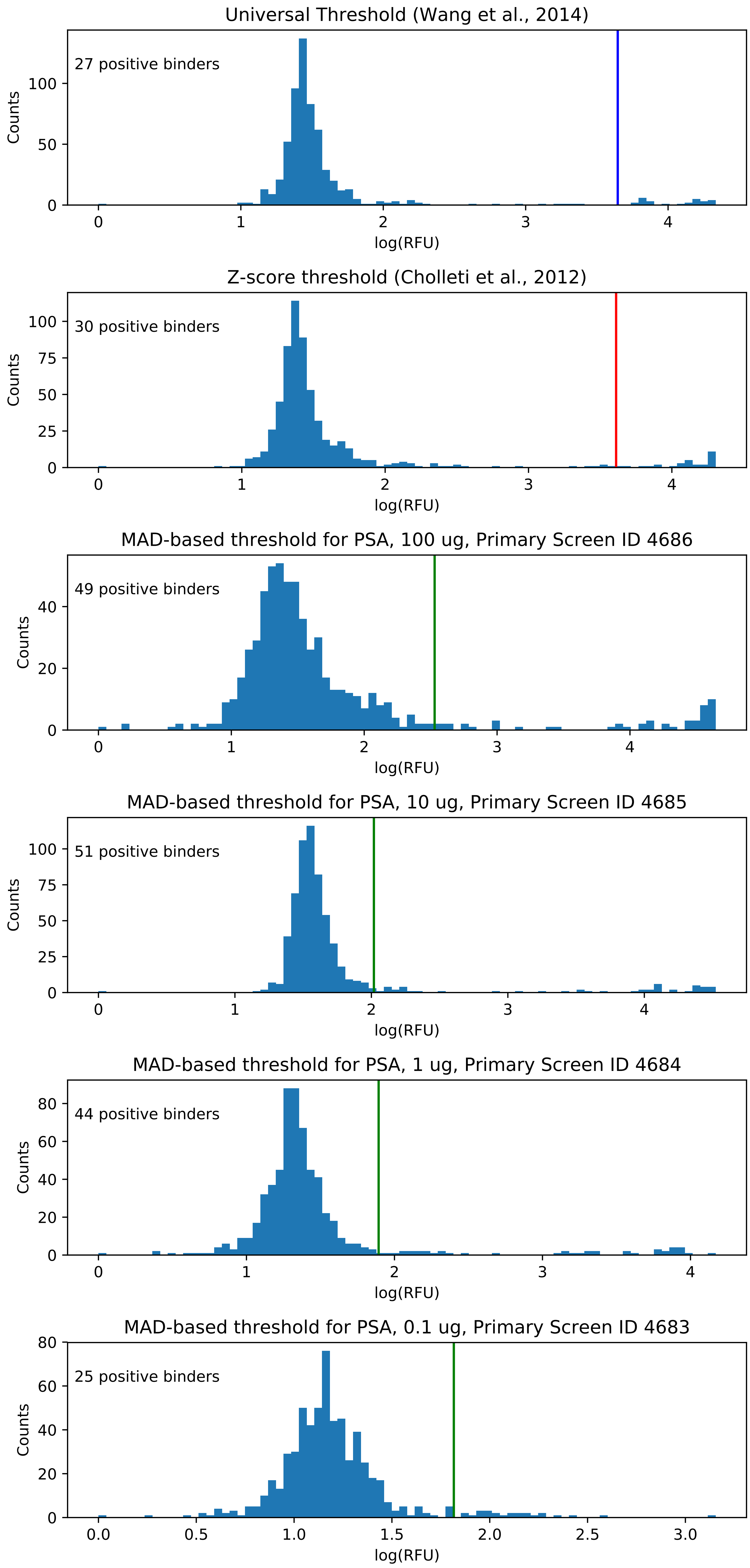
Universal Threshold (Wang et al., 2014)
27 positive binders
Z-score threshold (Cholleti et al., 2012)
30 positive binders
MAD-based threshold for PSA, 100 ug, Primary Screen ID 4686
49 positive binders
MAD-based threshold for PSA, 10 ug, Primary Screen ID 4685
51 positive binders
MAD-based threshold for PSA, 1 ug, Primary Screen ID 4684
44 positive binders
MAD-based threshold for PSA, 0.1 ug, Primary Screen ID 4683
25 positive binders
Counts
log(RFU)

RCA I

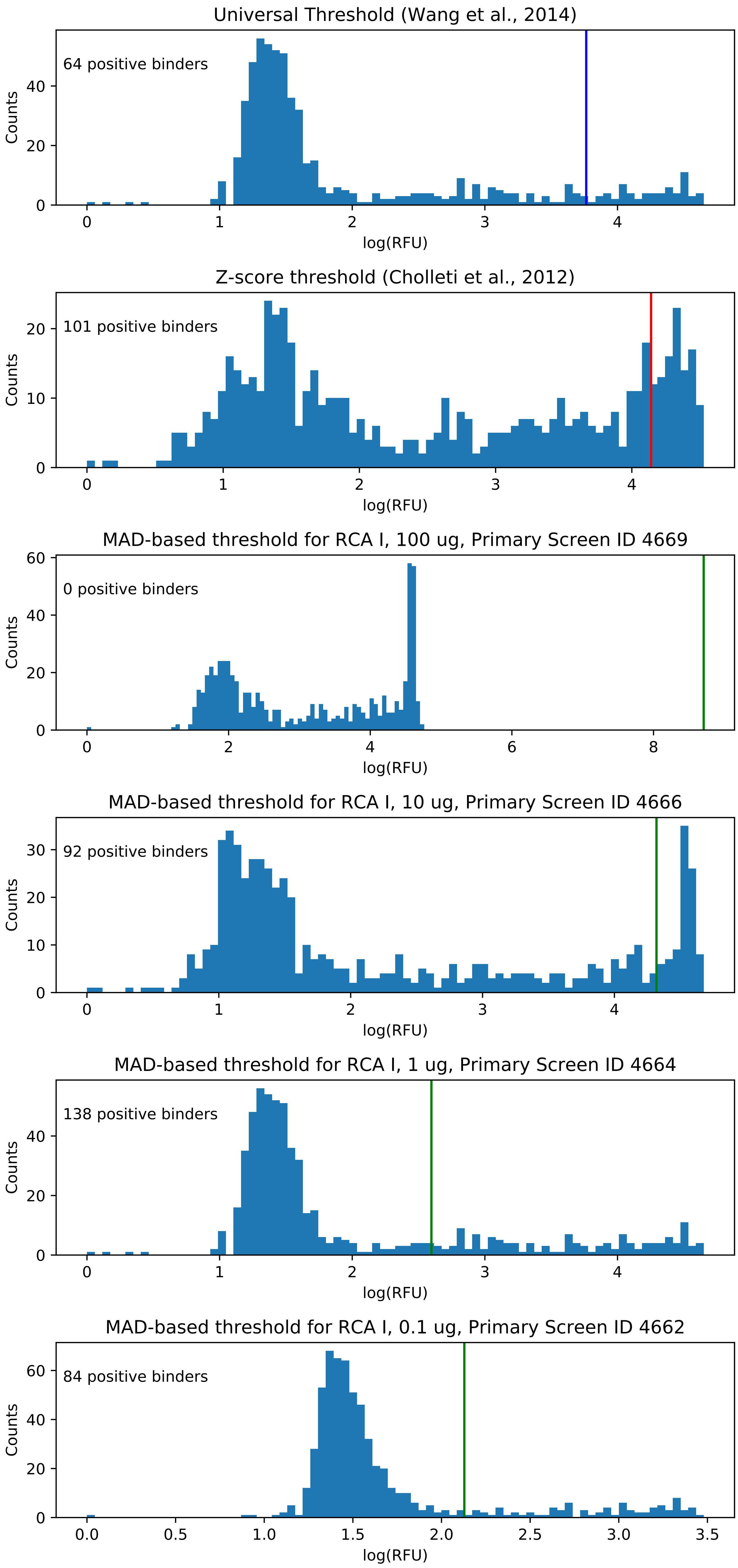
Universal Threshold (Wang et al., 2014)
64 positive binders
Counts
log(RFU)
Z-score threshold (Cholleti et al., 2012)
101 positive binders
Counts
log(RFU)
MAD-based threshold for RCA I, 100 ug, Primary Screen ID 4669
0 positive binders
Counts
log(RFU)
MAD-based threshold for RCA I, 10 ug, Primary Screen ID 4666
92 positive binders
Counts
log(RFU)
MAD-based threshold for RCA I, 1 ug, Primary Screen ID 4664
138 positive binders
Counts
log(RFU)
MAD-based threshold for RCA I, 0.1 ug, Primary Screen ID 4662
84 positive binders
Counts
log(RFU)

SBA

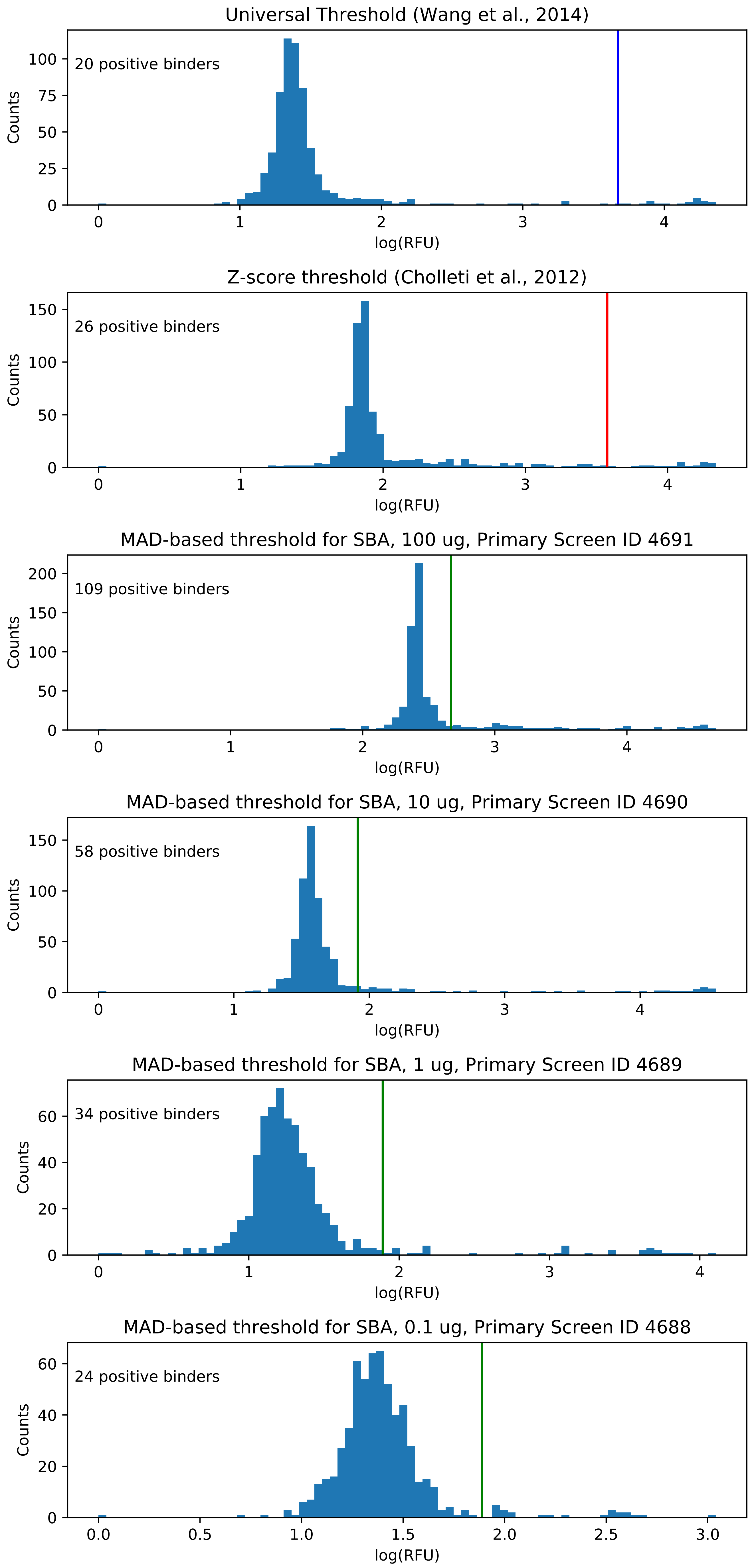
Universal Threshold (Wang et al., 2014)
20 positive binders
Counts
log(RFU)
Z-score threshold (Cholleti et al., 2012)
26 positive binders
Counts
log(RFU)
MAD-based threshold for SBA, 100 ug, Primary Screen ID 4691
109 positive binders
Counts
log(RFU)
MAD-based threshold for SBA, 10 ug, Primary Screen ID 4690
58 positive binders
Counts
log(RFU)
MAD-based threshold for SBA, 1 ug, Primary Screen ID 4689
34 positive binders
Counts
log(RFU)
MAD-based threshold for SBA, 0.1 ug, Primary Screen ID 4688
24 positive binders
Counts
log(RFU)

SNA

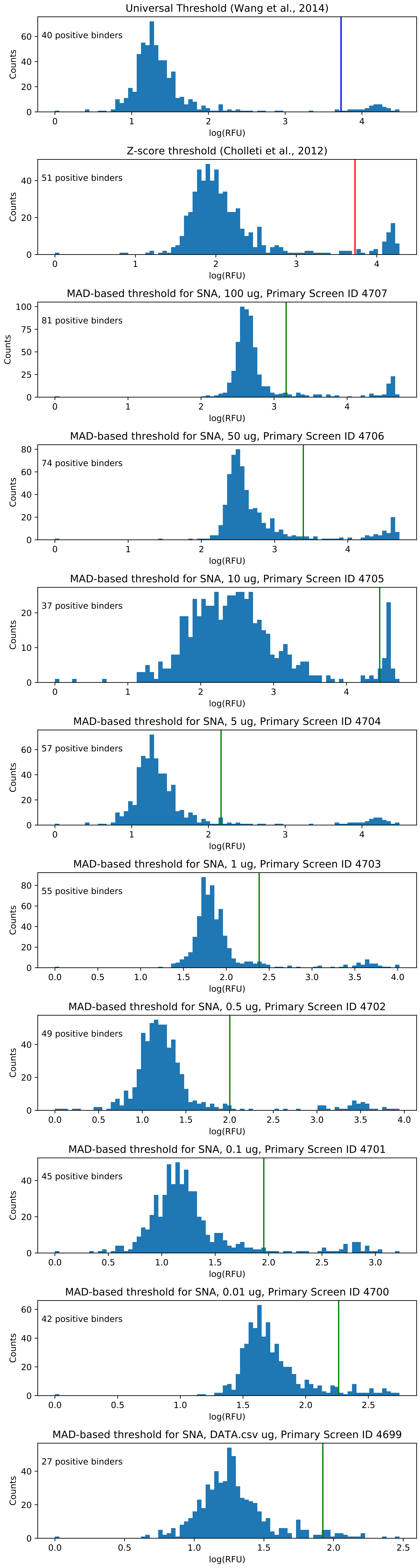
Universal Threshold (Wang et al., 2014)
40 positive binders
Counts
log(RFU)
Z-score threshold (Cholleti et al., 2012)
51 positive binders
Counts
log(RFU)
MAD-based threshold for SNA, 100 ug, Primary Screen ID 4707
81 positive binders
Counts
log(RFU)
MAD-based threshold for SNA, 50 ug, Primary Screen ID 4706
74 positive binders
Counts
log(RFU)
MAD-based threshold for SNA, 10 ug, Primary Screen ID 4705
37 positive binders
Counts
log(RFU)
MAD-based threshold for SNA, 5 ug, Primary Screen ID 4704
57 positive binders
Counts
log(RFU)
MAD-based threshold for SNA, 1 ug, Primary Screen ID 4703
55 positive binders
Counts
log(RFU)
MAD-based threshold for SNA, 0.5 ug, Primary Screen ID 4702
49 positive binders
Counts
log(RFU)
MAD-based threshold for SNA, 0.1 ug, Primary Screen ID 4701
45 positive binders
Counts
log(RFU)
MAD-based threshold for SNA, 0.01 ug, Primary Screen ID 4700
42 positive binders
Counts
log(RFU)
MAD-based threshold for SNA, DATA.csv ug, Primary Screen ID 4699
27 positive binders
Counts
log(RFU)

UEA I

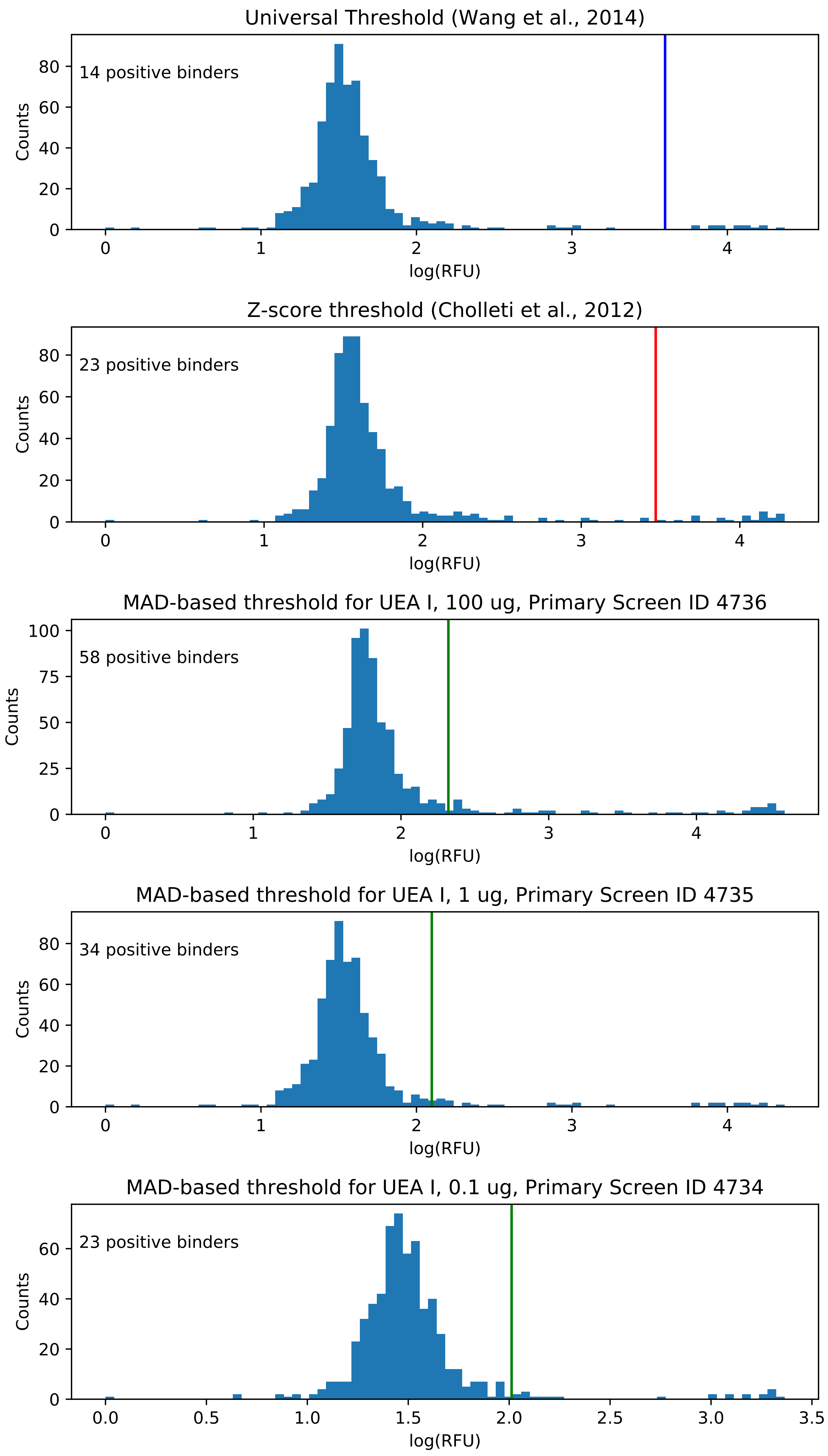
Universal Threshold (Wang et al., 2014)
14 positive binders
Z-score threshold (Cholleti et al., 2012)
23 positive binders
MAD-based threshold for UEA I, 100 ug, Primary Screen ID 4736
58 positive binders
MAD-based threshold for UEA I, 1 ug, Primary Screen ID 4735
34 positive binders
MAD-based threshold for UEA I, 0.1 ug, Primary Screen ID 4734
23 positive binders
Counts
log(RFU)

WGA

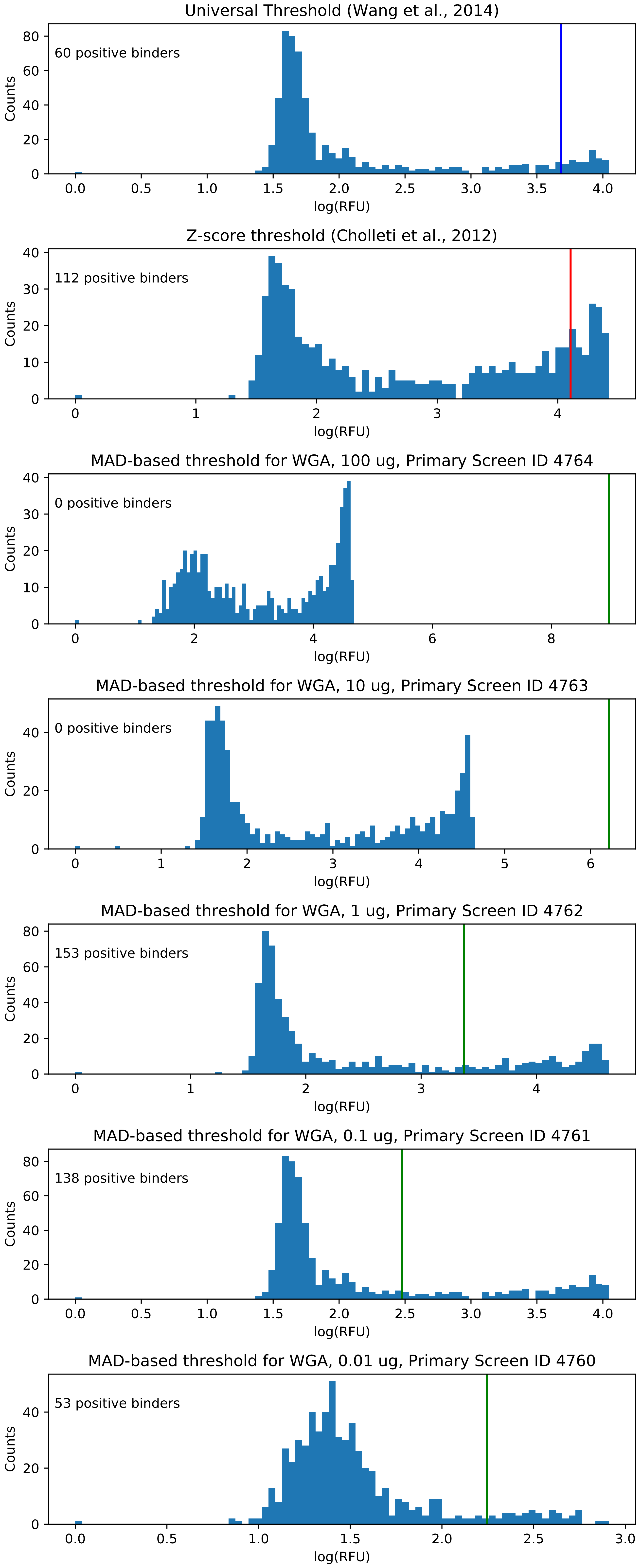
Universal Threshold (Wang et al., 2014)
60 positive binders
Z-score threshold (Cholleti et al., 2012)
112 positive binders
MAD-based threshold for WGA, 100 ug, Primary Screen ID 4764
0 positive binders
MAD-based threshold for WGA, 10 ug, Primary Screen ID 4763
0 positive binders
MAD-based threshold for WGA, 1 ug, Primary Screen ID 4762
153 positive binders
MAD-based threshold for WGA, 0.1 ug, Primary Screen ID 4761
138 positive binders
MAD-based threshold for WGA, 0.01 ug, Primary Screen ID 4760
53 positive binders
Counts
log(RFU)
