# Supplementary material for: Identifying glycan motifs using a novel subtree mining approach
Source: BMC Bioinformatics. 2020 Feb 4;21:42. doi: 10.1186/s12859-020-3374-4 (PMC7001330; doi:10.1186/s12859-020-3374-4)
Supplement: Supplementary file 7 — Additional file 7 Lectin specificities. Experimentally characterised lectin specificities for all lectins examined in this study. [file 12859_2020_3374_MOESM7_ESM.pdf]

**Lectin**	**Selectivity**	**Reference**
ABA	T antigen and terminal GlcNAc	[1]
ConA	N-glycans, particularly oligomannose	[2]
DBA	Terminal GalNAc	[3]
GSL I B_4	α-linked Gal	[4]
Influenza HA (A/Puerto Rico/8/34) (H1N1)	α2-6-linked sialic acid	[5]
Influenza HA (A/harbor seal/Massachusetts/1/2011) (H3N8)	α2-3-linked sialic acid	[6]
Human DC-SIGN tetramer	Oligomannose N-glycans and Lewis X	[7]
Jacalin	Core 1, Core 2 O-glycans and (*6)GalNAcα	[8]
LCA	Mannose oligosaccharides, enhanced by core-fucosylation	[9]
MAL I	α2-3-linked sialic acid	[10]
MAL II	α2-3-linked sialic acid and sulfated Gal	[11]
PHA-E	Bi-antennary N-gycans with terminal Gal, enhanced by bisecting GlcNAc	[12]
PHA-L	Tri- and tetra- antennary N-glycans	[13]
PNA	T antigen	[14]
PSA	α-linked Gal and core fucoses	[15,16]
RCA I	Terminal β-linked Gal and terminal Neu5Acα2-6Galβ	[17,18]
SBA	Terminal GalNAc and Gal	[19]
SNA	α2-6-linked sialic acid	[20]
UEA I	Blood Group O antigen	[21]
WGA	Terminal GlcNAc	[22]

References:

1. Nakamura-Tsuruta S, Kominami J, Kuno A, Hirabayashi J. Evidence that Agaricus bisporus agglutinin (ABA) has dual sugar-binding specificity. Biochem Biophys Res Commun. 2006;347: 215–220.
2. Brewer CF, Bhattacharyya L. Specificity of concanavalin A binding to asparagine-linked glycopeptides. A nuclear magnetic relaxation dispersion study. J Biol Chem. 1986;261: 7306–7310.
3. Hamelryck TW, Loris R, Bouckaert J, Dao-Thi MH, Strecker G, Imberty A, et al. Carbohydrate binding, quaternary structure and a novel hydrophobic binding site in two legume lectin oligomers from Dolichos biflorus. J Mol Biol. 1999;286: 1161–1177.
4. Kirkeby S, Moe D. Binding of Griffonia simplicifolia 1 isolectin B4 (GS1 B4) to alpha-galactose antigens. Immunol Cell Biol. 2001;79: 121–127.
5. Couceiro JN, Paulson JC, Baum LG. Influenza virus strains selectively recognize sialyloligosaccharides on human respiratory epithelium; the role of the host cell in selection of hemagglutinin receptor specificity. Virus Res. 1993;29: 155–165.
6. Hussein ITM, Krammer F, Ma E, Estrin M, Viswanathan K, Stebbins NW, et al. New England harbor seal H3N8 influenza virus retains avian-like receptor specificity. Sci Rep. 2016;6: 21428.
7. van Liempt E, Bank CMC, Mehta P, Garciá-Vallejo JJ, Kawar ZS, Geyer R, et al. Specificity of DC-SIGN for mannose- and fucose-containing glycans. FEBS Lett. 2006;580: 6123–6131.
8. Tachibana K, Nakamura S, Wang H, Iwasaki H, Tachibana K, Maebara K, et al. Elucidation of binding specificity of Jacalin toward O-glycosylated peptides: quantitative analysis by frontal affinity chromatography. Glycobiology. 2006;16: 46–53.
9. Maupin KA, Liden D, Haab BB. The fine specificity of mannose-binding and galactose-binding lectins revealed using outlier motif analysis of glycan array data. Glycobiology. 2012;22: 160–169.
10. Knibbs RN, Goldstein IJ, Ratcliffe RM, Shibuya N. Characterization of the carbohydrate binding specificity of the leukoagglutinating lectin from Maackia amurensis. Comparison with other sialic acid-specific lectins. J Biol Chem. 1991;266: 83–88.
11. Geisler C, Jarvis DL. Effective glycoanalysis with Maackia amurensis lectins requires a clear understanding of their binding specificities. Glycobiology. 2011;21: 988–993.
12. Yamashita K, Hitoi A, Kobata A. Structural determinants of Phaseolus vulgaris erythroagglutinating lectin for oligosaccharides. J Biol Chem. 1983;258: 14753–14755.
13. Kaneda Y, Whittier RF, Yamanaka H, Carredano E, Gotoh M, Sota H, et al. The High Specificities of Phaseolus vulgaris Erythro- and Leukoagglutinating Lectins for Bisecting GlcNAc or β1–6-Linked Branch Structures, Respectively, Are Attributable to Loop B. J Biol Chem. 2002;277: 16928–16935.
14. Chacko BK, Appukuttan PS. Peanut (Arachis hypogaea) lectin recognizes alpha-linked galactose, but not N-acetyl lactosamine in N-linked oligosaccharide terminals. Int J Biol Macromol. 2001;28: 365–371.
15. Suzuki T, Sugiyama K, Hirai H, Ito H, Morita T, Dohra H, et al. Mannose-specific lectin from the mushroom Hygrophorus russula. Glycobiology. 2012;22: 616–629.
16. Tateno H, Nakamura-Tsuruta S, Hirabayashi J. Comparative analysis of core-fucose-binding lectins from Lens culinaris and Pisum sativum using frontal affinity chromatography. Glycobiology. 2009;19: 527–536.
17. Itakura Y, Nakamura-Tsuruta S, Kominami J, Sharon N, Kasai K-I, Hirabayashi J. Systematic comparison of oligosaccharide specificity of Ricinus communis agglutinin I and Erythrina lectins: a search by frontal affinity chromatography. J Biochem. 2007;142: 459–469.
18. Song X, Yu H, Chen X, Lasanajak Y, Tappert MM, Air GM, et al. A sialylated glycan microarray reveals novel interactions of modified sialic acids with proteins and viruses. J Biol Chem. 2011;286: 31610–31622.
19. Lotan R, Skutelsky E, Danon D, Sharon N. The purification, composition, and specificity of the anti-T lectin from peanut (Arachis hypogaea). J Biol Chem. 1975;250: 8518–8523.
20. Shibuya N, Goldstein IJ, Broekaert WF, Nsimba-Lubaki M, Peeters B, Peumans WJ. The elderberry (Sambucus nigra L.) bark lectin recognizes the Neu5Ac(alpha 2-6)Gal/GalNAc sequence. J Biol Chem. 1987;262: 1596–1601.
21. Matsumoto I, Osawa T. Purification and characterization of an anti-H(O) phytohemagglutinin of Ulex europeus. Biochim Biophys Acta. 1969;194: 180–189.
22. Yamamoto K, Tsuji T, Matsumoto I, Osawa T. Structural requirements for the binding of oligosaccharides and glycopeptides to immobilized wheat germ agglutinin. Biochemistry. 1981;20: 5894–5899.
