# Supplementary material for: Identifying glycan motifs using a novel subtree mining approach
Source: BMC Bioinformatics. 2020 Feb 4;21:42. doi: 10.1186/s12859-020-3374-4 (PMC7001330; doi:10.1186/s12859-020-3374-4)
Supplement: Supplementary file 8 — Additional file 8 Motifs from GLYMMR and glycan motif miner. Motifs extracted using GLYMMR and Glycan Miner Tool for a range of glycan microarray datasets. [file 12859_2020_3374_MOESM8_ESM.zip › DBA.pdf]

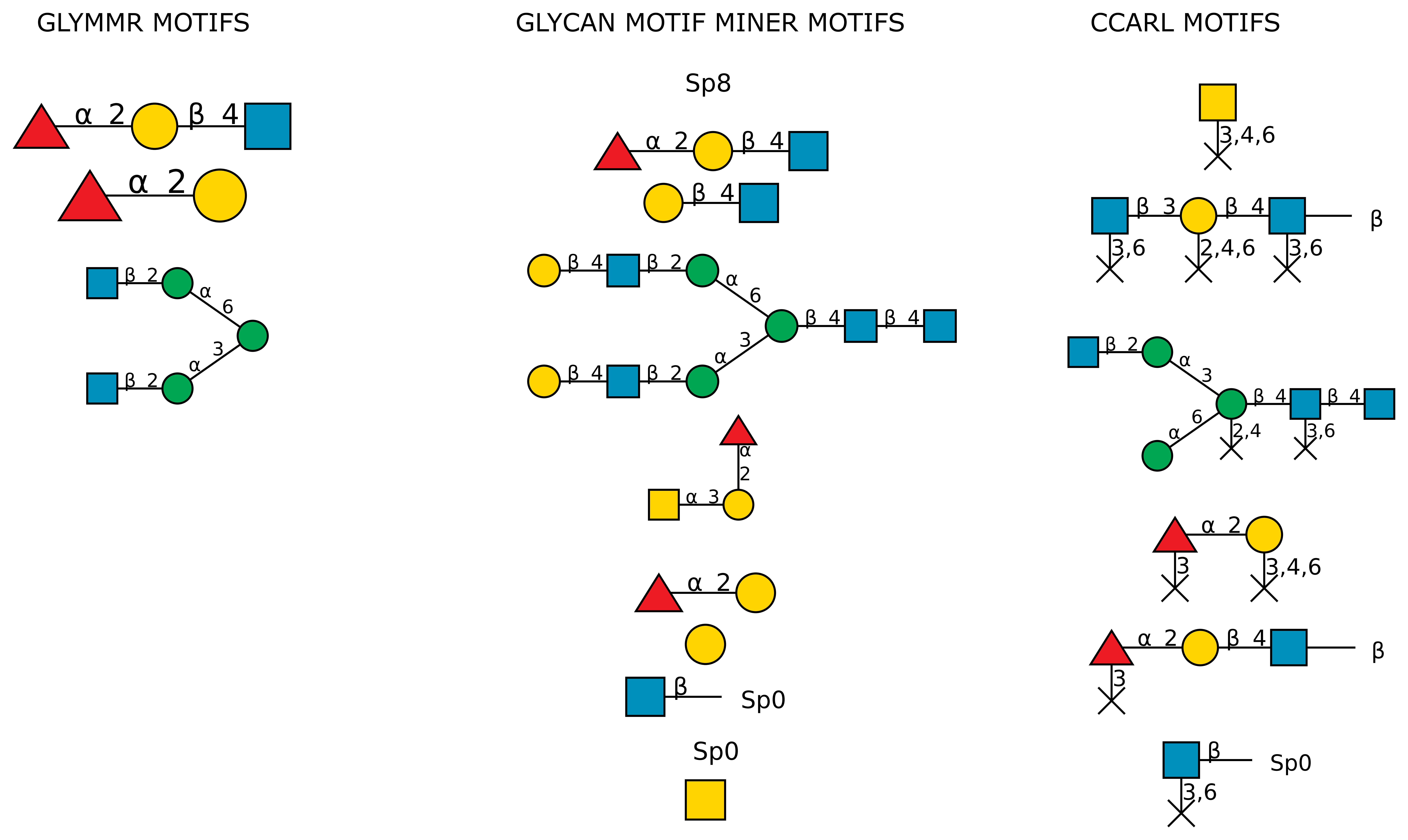
GLYMMR MOTIFS
GLYCAN MOTIF MINER MOTIFS
CCARL MOTIFS
Sp8
Sp0
Sp0
Sp0
